# Reporting quality of qualitative health studies published by Peruvian authors: A scoping review

**DOI:** 10.1371/journal.pone.0351494

**Published:** 2026-06-23

**Authors:** Cristhian Rojas-Miliano, Andrea Dámaso-Román, Guillermo Almeida-Huanca, Nikol Mayo-Puchoc, Yolanda Viguria-Chavez, Andy Rick Sánchez-Villena, Alvaro Taype-Rondan, Rubí Paredes-Angeles

**Affiliations:** 1 Universidad Nacional del Centro del Perú, Junín, Perú; 2 Centro de Salud Mental Comunitario Pangoa, Junín, Perú; 3 Facultad de Psicología, Universidad Nacional Mayor de San Marcos, Lima, Perú; 4 Facultad de Psicología, Grupo de Investigación en Psicología Social y Desarrollo Humano, Universidad Nacional Mayor de San Marcos, Lima, Perú; 5 Instituto Peruano de Orientación Psicológica, Lima, Perú; 6 Universidad Peruana Cayetano Heredia, School of Public Health, Lima, Perú; 7 Universidad Señor de Sipán, Vicerrectorado de Investigación, Chiclayo, Perú; 8 Unidad de Investigación para la Generación y Síntesis de Evidencias en Salud, Vicerrectorado de Investigación, Universidad San Ignacio de Loyola, Lima, Perú; 9 EviSalud – Evidencias en Salud, Lima, Perú; 10 Universidad Científica del Sur, Lima, Peru; Universidad Peruana Cayetano Heredia Facultad de Medicina, PERU

## Abstract

Qualitative research offers valuable insights into complex social and behavioral processes in the health sciences; however, its contribution depends on the quality and transparency of reporting. This scoping review aimed to describe the methodological characteristics and reporting quality of qualitative studies published by Peruvian authors between 2022 and 2025. Following Prisma-scr guidelines, a systematic search was conducted in SCOPUS, and reporting quality was assessed using the Standards for Reporting Qualitative Research (SRQR) tool. A total of 147 qualitative studies were included. Most were published in English (49.0%), had a median of five authors; 71.4% had a first author affiliated with a Peruvian institution, and 58.5% involved exclusively Peruvian authors. Studies were mainly conducted in Lima (50.3%). Ethics committee approval was not reported in 24.5% of studies, and 40.8% did not provide an ethics approval code. Adherence to SRQR items was highest for abstracts (95.9%), study purpose (95.2%), and presentation of findings (93.9%), whereas reporting of title elements (31.3%), qualitative approach (49.0%), and researcher reflexivity (37.4%) was limited. Publication in Spanish and Peruvian first authorship were associated with lower SRQR scores, whereas a higher number of authors and international collaboration were associated with higher scores. Overall, qualitative studies published by Peruvian authors showed variable reporting quality, with important methodological and ethical aspects frequently under reported, highlighting the need to strengthen reporting practices, particularly for core qualitative components.

## Introduction

Qualitative research provides a deeper understanding of complex social and behavioral processes in health sciences [1]. Unlike the quantitative methods, qualitative studies seek to explore, narrate and explain experiences, perceptions, and contexts, offering a transformative perspective that enriches evidence-based decision-making [2,3]. Thus, qualitative research enhances the contextual understanding necessary for developing grounded health interventions [1,2].

However, the usefulness of qualitative findings can be affected by the quality of reporting [4]. Although the inherent heterogeneity of qualitative research reporting reflects its richness and diversity, this variability can also impede systematic evaluation of quality and comparison across studies [5]. Clear, complete, and transparent reporting enables readers to better assess the credibility, transfer ability, and methodological rigor of qualitative studies.

Some international guidelines have been developed to standardize reporting in qualitative research, including the Standards for Reporting Qualitative Research (SRQR) [6], the Consolidated Criteria for Reporting Qualitative Research (COREQ) [7], and the Enhancing Transparency in Reporting the synthesis of Qualitative research (ENTREQ) [8]. Unlike COREQ, which is tailored for interviews and focus groups, and ENTREQ, which is focused on qualitative synthesis studies, SRQR provides a comprehensive framework that authors can use across a wide range of qualitative methodologies [6,9]. Thus, previous research has employed the SRQR to evaluate reporting quality in qualitative studies conducted in diverse health domains, including urology [10], pediatrics [11], infectious diseases [12], and breast cancer [13].

In Peru, scientific research output has increased, with an estimated annual growth rate of 13.6%, with 70.2% corresponds to health sciences [14]. A previous study examined the scientific production of qualitative research in Peru found that the publication trend has increased since 2013; however, it did not assess the reporting quality of these studies [15]. The absence of such information limits understanding of the transparency and reporting standards within this research field in Peru. Assessing these aspects may help identify strengths, weaknesses, and opportunities for improvement among researchers, reviewers, and editors of scientific journals [16]. Therefore, this study aimed to describe the methodological characteristics and assess the reporting quality of qualitative studies conducted in populations residing in Peru and involving at least one author affiliated with a Peruvian institution.

## Methods

This scoping review was carried out and reported following the guidelines of the Preferred Reporting Items for Systematic Reviews and Meta-Analyses Extension for Scoping Reviews (Prisma-scr) [17]. The completed PRISMA-ScR checklist is provided in S1 Table. A detailed study protocol was developed prior to study initiation and made publicly available (https://osf.io/jes6v/files/3rn52).

### Search strategy

A systematic search was performed in Scopus on October 15, 2025, using the following search strategy to identify qualitative health studies authored by researchers affiliated with institutions in Peru and published between 2022 and 2025: AFFILCOUNTRY (peru) AND TITLE-ABS (qualitative) AND ((SUBJAREA (MEDI OR NURS OR DENT OR HEAL) OR SUBJAREA (PSYC))) AND PUBYEAR>2021 AND PUBYEAR < 2026

### Study eligibility

We included original articles authored by researchers affiliated with institutions in Peru, that were published in scientific journals between 2022 and 2025 and presented findings from qualitative studies. This timeframe was chosen to reflect the current state of the field. Only studies in which at least 25% of participants were based in Peru were considered to ensure meaningful representation of the Peruvian population while allowing the inclusion of international studies. Publications related to health disciplines, including medicine, nursing, dentistry, psychology, and other health professions, were included. Mixed-methods studies were excluded due to the need for specific assessment tools. No language restrictions were applied.

### Studies selection

Records were uploaded to the “Rayyan” software (Qatar Computing Research Institute, Doha, Qatar; https://www.rayyan.ai/). Six authors in pairs independently screened the records applying the selection criteria. Disagreements between the reviewers were resolved through discussion among the reviewers or with the collaboration of other authors.

### Data extraction

To ensure consistency, a pilot data extraction was conducted prior to the main process. Subsequently, six authors worked in pairs to independently extract data from each study, recording all information in a predesigned Excel spreadsheet. The research team consisted of professionals in Medicine and Psychology with experience in conducting health research.

For each included study, we collected information on its general and methodological characteristics, including: language of the study (English, Spanish, or both English and Spanish), whether the study was published in a Peruvian journal (yes or no), number of authors (divided in tertiles), country of the first author’s institutional affiliation, international collaboration, defined as the inclusion of at least one co-author with an institutional affiliation in a country other than Peru (yes or no), study population, sample size, study setting (community settings, health facilities or educational centers), Peruvian region where the study was conducted, multicountry study, defined as a study that included research sites in countries other than Peru (only Peru or Peru and others countries), ethics committee approval (whether the article reported approval from at least one ethics committee), and ethics approval code reported (whether the article, supplementary material, or appendix included the ethics approval code).

Additionally, we assessed the quality of reporting using the SRQR checklist. The data extraction matrix included two components for the SRQR score: one for the assigned score and another to document the rationale, including verbatim excerpts from the reviewed articles to justify each rating. Each pair of investigators independently extracted study characteristics and SRQR items, and then met to compare each cell and reach consensus, resulting in a final extraction. Discrepancies that could not be resolved were discussed during weekly team meetings until agreement was achieved.

### Quality of reporting

We used the SRQR 21-item checklist [6], which provides guidance on transparency and completeness in qualitative research reporting. Each of the 21 items was assessed to determine whether it was ‘reported’ (score = 2 points), ‘partially reported’ (score = 1 point), or ‘not reported’ (score = 0 points).

The total SRQR score ranged from 0 to 42 points. An item was considered ‘reported’ when the authors presented the required information in accordance with the SRQR guidelines, ‘partially reported’ when the authors provided some, but not all, of the required information, and ‘not reported’ when the authors failed to provide any of the required information according to the SRQR reporting standard [9]. Also, we created the variable “adequate SRQR reporting,” defined as belonging to the highest tertile (≥ 37 points) of the SRQR score. Given the absence of established or validated cut-off points for SRQR scores, this data-driven approach was used to identify studies with comparatively better reporting quality.

This SRQR checklist assessed the following items:

S1. Title. Concise description of the nature and topic of the study. Identifying the study as qualitative/mixed or indicating the approach (e.g., ethnography, grounded theory) or data collection methods (e.g., interview, focus group) is recommended.

S2. Abstract. Summary of key elements of the study using the abstract format of the intended publication; typically includes background, purpose, methods, results, and conclusions.

S3. Introduction 1. Description and significance of the problem/phenomenon studied; review of relevant theory and empirical work problem statement.

S4. Introduction 2. Purpose of the study and specific objectives or questions.

S5. Methods 1. Qualitative approach (e.g., ethnography, grounded theory, case study, phenomenology, narrative research) and guiding theory if appropriate; identifying the research paradigm (e.g., postpositivist, constructivist/ interpretivist) is also recommended; rationale.

S6. Methods 2. Researchers’ characteristics that may influence the research, including personal attributes, qualifications/experience, relationship with participants, assumptions, and/or presuppositions; potential or actual interaction between researchers’ characteristics and the research questions, approach, methods, results, and/or transferability.

S7. Methods 3. Context. Setting/site and salient contextual factors; rationale.

S8. Methods 4. Sampling strategy. How and why research participants, documents, or events were selected; criteria for deciding when no further sampling was necessary (e.g., sampling saturation); rationale.

S9. Methods 5. Documentation of approval by an appropriate ethics review board and participant consent, or explanation for lack thereof; other confidentiality and data security issues.

S10. Methods 6. Types of data collected; details of data collection procedures including (as appropriate) start and stop dates of data collection and analysis, iterative process, triangulation of sources/methods, and modification of procedures in response to evolving study findings; rationale.

S11. Methods 7. Description of instruments (e.g., interview guides, questionnaires) and devices (e.g., audio recorders) used for data collection; if/how the instrument(s) changed over the course of the study.

S12. Methods (or results) 8. Number and relevant characteristics of participants, documents, or events included in the study; level of participation (could be reported in results).

S13. Methods 9. Methods for processing data prior to and during analysis, including transcription, data entry, data management and security, verification of data integrity, data coding, and anonymization/deidentification of excerpts.

S14. Methods 10. Process by which inferences, themes, etc., were identified and developed, including the researchers involved in data analysis; usually references a specific paradigm or approach; rationale.

S15. Methods 11. Techniques to enhance trustworthiness and credibility of data analysis (e.g., member checking, audit trail, triangulation); rationale.

S16. Results 1. Main findings (e.g., interpretations, inferences, and themes); might include development of a theory or model, or integration with prior research or theory.

S17. Results 2. Evidence (e.g., quotes, field notes, text excerpts, photographs) to substantiate analytic findings.

S18. Discussion 1. Short summary of main findings; explanation of how findings and conclusions connect to, support, elaborate on, or challenge conclusions of earlier research; discussion of scope of application/generalizability; identification of unique contribution(s) to the field.

S19. Discussion 2. Trustworthiness and limitations of findings.

S20. Conflicts of interest. Potential sources of influence or perceived influence on study conduct and conclusions; how these were managed.

S21. Funding. Sources of funding and other support; role of funders in data collection, interpretation, and reporting.

### Data analysis

Data were entered into Microsoft Excel, and all statistical analyses were performed using Stata software, version 19.0 (StataCorp, College Station, TX, USA; https://www.stata.com/). Descriptive analyses included absolute and relative frequencies for categorical variables and medians with interquartile ranges for quantitative variables. Additionally, an exploratory analysis of factors associated with adequate SRQR reporting was conducted. For this purpose, crude prevalence ratios (PR) with 95% confidence intervals (CI) were estimated using Poisson regression with robust variance.

Spatial data processing and visualization were performed in R (version 4.2.2; R Foundation for Statistical Computing, Vienna, Austria). Administrative boundary data for Peru were obtained from the “mapsPERU” package (version 2.0.0), which provides publicly available vector-based geospatial data. Spatial data were managed using the “sf” package (version 1.0.16). Data manipulation was conducted using “dplyr” package (version 1.1.4) and “ggplot2” package (version 3.5.1). No proprietary basemaps were used.

### Ethical statement

Ethics committee approval was not required because this study involved only a review of previously published literature and did not include human participants.

## Results

**Fig 1:**
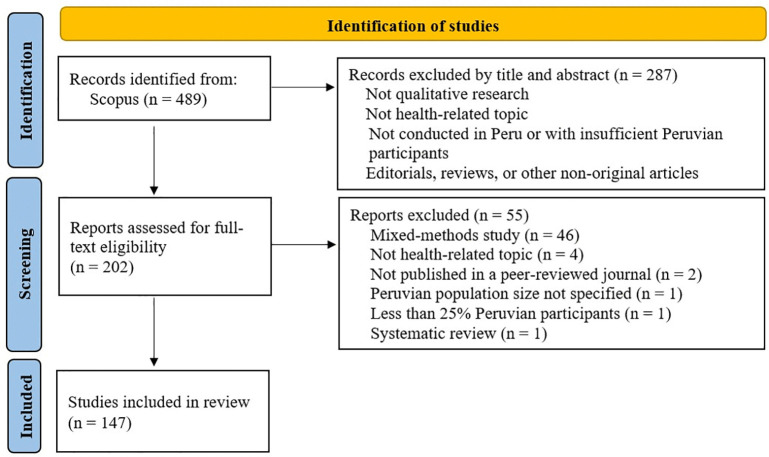
Flowchart of the article selection process.

The search yielded 489 records, of which 202 were selected for full-text assessment after title and abstract screening. Records were excluded at this stage because they did not meet the eligibility criteria, including studies that were not qualitative, were not related to health sciences, did not involve populations residing in Peru, or corresponded to non-original publications. Of these, 55 reports were excluded after full-text assessment for the following reasons: mixed-methods design (n = 46), non–health-related topic (n = 4), publication outside a peer-reviewed journal (n = 2), unspecified Peruvian population size (n = 1), inclusion of less than 25% Peruvian participants (n = 1), and systematic review design (n = 1). Consequently, a total of 147 studies were included in the final analysis (Fig 1). The characteristics of the included studies can be found in S2 Table, while the list of excluded reports is presented in S3 Table.

Across the included studies, most were published in English (49.0%) and were not published in a Peruvian journal (88.4%). The median number of authors was five, and 37.4% were studies with four to seven authors. Regarding affiliation, the first author most frequently had their primary institutional affiliation in Peru (71.4%), and most studies involved only Peruvian authors (58.5%). The most frequent main population studied were health professionals (20.4%) and non-clinical stakeholders (20.4%). The majority of studies were conducted in community settings (42.2%). Most studies included only participants from Peru (91.8%). Ethical procedures were not reported in 24.5% of the studies, and 40.8% did not provide an ethics approval code. Adequate SRQR reporting was observed in 32.7% of the included studies (Table 1).

**Table 1:** Characteristics of included studies (n = 147).

Characteristics	n (%)
**Language**	
English	72 (49.0)
Spanish	63 (42.8)
English and Spanish	12 (8.2)
**Published in a Peruvian journal**	
No	130 (88.4)
Yes	17 (11.6)
Number of authors*	5 [4 – 8]
1–4	53 (36.1)
5–7	55 (37.4)
8–36	39 (26.5)
**Country of the first author’s first affiliation**	
Peru	105 (71.4)
United States	27 (18.4)
United Kingdom	4 (2.7)
Switzerland	3 (2.0)
Spain	2 (1.4)
Canada	2 (1.4)
Other countries	4 (2.7)
**International collaboration**	
Only authors from Peru	86 (58.5)
At least one international author	61 (41.5)
**Main population of the study**	
Health professionals (clinicians and care personnel)	30 (20.4)
Policy makers, administrators, program managers, community leaders, stakeholders, and other non-clinical populations	30 (20.4)
Patients, service users, and individuals living with health conditions	25 (17.0)
Mothers, fathers, caregivers, and families	22 (15.0)
Children, adolescents, and young people	21 (14.3)
Indigenous peoples, individuals from rural communities, and culturally specific groups	19 (12.9)
Sample size*	15 [12 - 30]
**Study setting**	
Community settings	62 (42.2)
Health facilities	46 (31.2)
Educational centers	20 (13.6)
Others	18 (12.3)
Not reported	1 (0.7)
**Region where the study was conducted†**	
Lima	75 (50.3)
Lambayeque	29 (19.5)
Loreto	10 (6.8)
Junin	9 (6.1)
La Libertad	6 (4.1)
Others	37 (25.0)
Not reported	13 (8.8)
**Multicountry sample**	
Peru only	135 (91.8)
Peru and other countries	12 (8.2)
**Reported the ethics committee approval**	
Yes	111 (75.5)
No	36 (24.5)
**Reported the ethics approval code**	
Yes	87 (59.2)
No	60 (40.8)
Total SRQR (in points)*	34 [31-38]
**Adequate reporting‡**	
No	99 (67.3)
Yes	48 (32.7)

SRQR: Standards for Reporting Qualitative Research.

*Median [p25 - p75].

†For this variable, the percentages add up to more than 100% because some studies were conducted in more than one Peruvian region.

‡SRQR score (≥ 37 points).

**Fig 2:**
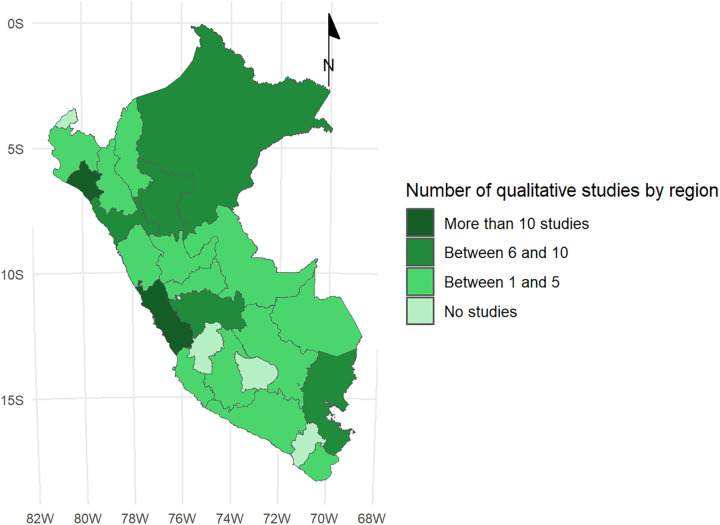
Number of qualitative studies conducted by region.

The highest number of qualitative studies were identified in Lima and Lambayeque, with 75 (50.3%) and 29 (19.5%) studies, respectively, while no studies were identified in Apurímac, Huancavelica, and Tumbes (Fig 2).

**Fig 3:**
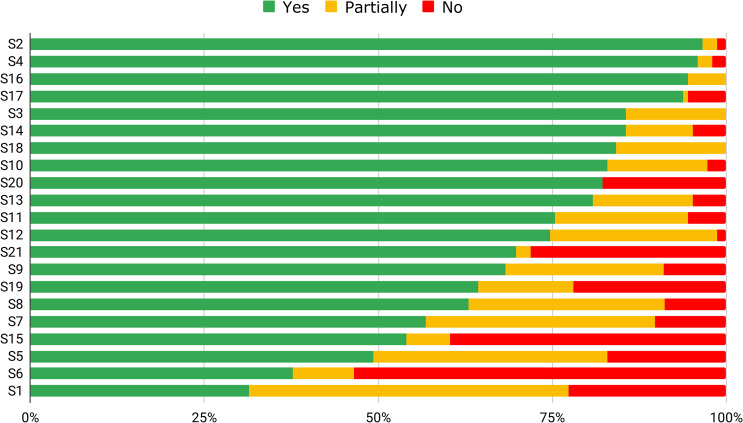
Fulfillment of the SRQR items among Peruvian qualitative studies (n = 147). SRQR: Standards for Reporting Qualitative Research. S1. Quality of title.S2. Quality of abstract.S3. Introduction. Background and problem description.S4. Introduction. Study purpose and objectives.S5. Methods: Qualitative approach and research paradigm.S6. Methods: Researcher characteristics and reflexivity.S7. Methods: Study context and setting.S8. Methods: Sampling strategy.S9. Methods: Ethical approval and consent.S10. Methods: Data collection procedures.S11. Methods: Data collection instruments.S12. Methods: Participant characteristics.S13. Methods: Data processing and management.S14. Methods: Data analysis process.S15. Methods: Trustworthiness and credibility techniques.S16. Results: Main findings.S17. Results: Supporting evidence for findings.S18. Discussion: Interpretation and contribution to literature.S19. Discussion: Limitations and trustworthiness.S20. Conflicts of interest.S21. Funding sources.

The SRQR items with the highest level of adherence were the abstract (S2), reported by 95.9% of studies, the statement of study purpose (S4), reported by 95.2%, and the presentation of main findings (S16), reported by 93.9%. On the other hand, only 31.3% of studies adequately reported the title elements (S1). Reporting of the qualitative approach or guiding paradigm (S5) was also limited, with 49.0% of studies meeting this criterion. Similarly, researchers’ characteristics and reflexivity (S6) were reported in just 37.4% of studies (Fig 3).

In the regression analysis, some study characteristics were associated with a higher prevalence of adequate SRQR reporting (score ≥ 37). Studies published in Spanish showed a lower frequence of adequate reporting compared with those published in English (PR = 0.12; 95% CI: 0.05–0.32). Studies with eight or more authors (PR = 3.47; 95% CI: 1.81–6.67), studies whose first author was affiliated with a non-Peruvian institution (PR = 0.40; 95% CI: 0.26–0.62), and studies involving international collaboration (PR = 2.35; 95% CI: 1.45–3.82), had a higher prevalence of adequate reporting. In contrast, neither the main population of interest nor publication in a Peruvian journal showed a statistically significant association (Table 2).

**Table 2:** Factors associated with adequate SRQR checklist reporting (n = 147).

Variables	Adequate SRQR reporting		RP (IC 95%)
	No (SRQR score < 37)	Yes (SRQR score ≥ 37)	
Language of the journal			
English	40 (47.6)	44 (52.4)	Ref.
Spanish	59 (93.7)	4 (6.3)	**0.12 (0.05 to 0.32)**
Published in a Peruvian journal			
No	87 (66.9)	43 (33.1)	Ref.
Yes	12 (70.6)	5 (29.4)	0.89 (0.41 to 1.94)
Number of authors			
1–4	44 (83.0)	9 (17.0)	Ref.
5–7	39 (70.9)	16 (29.1)	1.71 (0.83 to 3.54)
8–36	16 (41.0)	23 (59.0)	**3.47 (1.81 to 6.67)**
Country of the first author’s first affiliation			
Non Peru	18 (42.9)	24 (57.1)	Ref
Peru	81 (77.1)	24 (22.9)	**0.40 (0.26 to 0.62)**
International collaboration			
Only authors from Peru	68 (79.1)	18 (20.9)	Ref.
At least one international author	31 (50.8)	30 (49.2)	**2.35 (1.45 to 3.82)**
Main population of the study			
Children, adolescents, and young people	16 (76.2)	5 (23.8)	Ref.
Mothers, fathers, caregivers, and families	25 (83.3)	5 (16.7)	0.70 (0.23 to 2.13)
Indigenous peoples, individuals from rural communities, and culturally specific groups	12 (57.1)	9 (42.9)	1.80 (0.72 to 4.49)
Patients, service users, and individuals living with health conditions	14 (56.0)	11 (44.0)	1.85 (0.76 to 4.49)
Health professionals (clinicians and care personnel)	11 (55.0)	9 (45.0)	1.89 (0.76 to 4.69)
Policy makers, administrators, program managers, community leaders, stakeholders, and other non-clinical populations	21 (70.0)	9 (30.0)	1.26 (0.49 to 3.24)

SRQR: Standards for Reporting Qualitative Research.

PR: Prevalence ratio. 95% CI: 95% confidence interval. Ref: reference.

## Discussion

### Main results

A total of 147 qualitative studies were included. Most were published in English, had a median of five authors, and were conducted primarily in the capital city and in community settings. First authors were most commonly affiliated with Peruvian institutions, and the majority of studies were conducted exclusively by Peruvian authors. Ethics committee approval was not reported in nearly one quarter of the studies, and almost half did not provide an ethics approval code. The SRQR items with the highest adherence were the abstract (S2), the statement of study purpose (S4), and the presentation of main findings (S16), whereas title elements (S1), the qualitative approach or guiding paradigm (S5), and researchers’ characteristics and reflexivity (S6) were less frequently reported. Adequate SRQR reporting was associated with publication in English, a higher number of authors, non-Peruvian first-author affiliation, and international collaboration, with no significant associations observed for the study population or publication in Peruvian journals.

### Characteristics of the studies

Approximately half of the included studies were conducted in Lima, indicating a pronounced geographical concentration of qualitative research in Peru, consistent with prior evidence of higher scientific output in Lima, the capital of Peru [18,19]. This finding suggests that qualitative research follows the pattern of centralized scientific activity in Peru. Barriers to research production in other regions may be driven by structural inequities, such as limited access to research training and funding, as well as the concentration of institutions with greater research capacity in Lima [18]. Strengthening interregional collaboration, revising funding allocation mechanisms, and prioritizing equitable research capacity development may contribute to a more balanced national research landscape [20].

Nearly one quarter of the studies did not report ethical procedures, and over two fifths failed to include an ethics approval code. This finding may reflect two non-mutually exclusive explanations. On the one hand, some studies may not have been reviewed by an ethics committee, which is particularly concerning given that qualitative research typically involves close engagement with participants and may address sensitive topics, thereby requiring ethical oversight to safeguard participant welfare, informed consent, and confidentiality. On the other hand, these results may indicate shortcomings in reporting practices rather than deficiencies in ethical conduct, whereby ethical approval was obtained but not explicitly documented in the published reports.

Well-established ethical and regulatory frameworks, including the Declaration of Helsinki and the International Ethical Guidelines for Health-related Research Involving Humans issued by CIOMS, as well as current Peruvian regulations [21], stipulate that all research involving human participants must undergo prior review and approval by a research ethics committee. These provisions aim to safeguard participants’ rights, safety, and well-being, and to ensure adherence to fundamental ethical principles such as autonomy, confidentiality, and justice.

Although the first author’s primary institutional affiliation was predominantly Peruvian (71.4%), a substantial proportion of the studies involved international collaboration (41.5%). This pattern suggests that, despite local leadership in qualitative research production, international collaboration could play a meaningful role in the development of these studies. Such collaborations may reflect efforts to strengthen methodological rigor, access complementary expertise, and enhance the visibility of research conducted in Peru.

### Results of the SRQR checklist

The items most consistently reported included the presence of an abstract containing the key elements of the study (S2), the specification of the study objective in the introduction (S4), and the reporting of results, particularly the description of the main findings and the evidence supporting them (S16 and S17). In contrast, the least frequently addressed elements were the use of techniques to ensure the credibility of the findings (S15), the qualitative approach and research paradigm (S5), the characteristics of the researchers (S6), and the formulation of a title that accurately reflects the nature and focus of the study (S1).

Our findings align with a previous study of qualitative research in pediatric urology, where the abstract and the study objective were also among the most consistently reported items [10]. Moreover, our results are consistent with a previous research on qualitative studies of malaria in Colombia, which identified credibility techniques and the description of the methodological paradigm as among the least reported elements [12]. Another study examining qualitative research in community pharmacies likewise documented poor reporting of the title, researcher characteristics, and the nature of the study [9]. However, unlike those studies, which focused on specific populations or thematic areas, our analysis encompasses the qualitative scientific output of an entire region. This convergence suggests that there is a persistent tendency to report items that are commonly required in scientific manuscripts, irrespective of study design (e.g., abstract key aspects, objective, main findings), reflecting adherence to general reporting conventions rather than specific compliance with qualitative reporting guidelines. By contrast, several items that are fundamental to the rigor and transparency of qualitative research were among the least frequently reported.

This pattern indicates that while general reporting standards are largely met, less emphasis is placed on elements that are distinctive to qualitative inquiry and essential for the appraisal of methodological rigor. In this context, strengthening training in qualitative research reporting, encouraging the adoption of reporting guidelines such as SRQR by journals, and ensuring specialized peer review processes are essential to improve reporting quality.

### Factors associated with adequate SRQR reporting

Regarding associated factors, articles published in Spanish were associated with lower reporting quality scores. This may be explained by the tendency of non-English-speaking authors to prioritize publication in English due to its greater international visibility [22] and citation potential [23]. As a result, manuscripts with stronger methodological rigor and better adherence to reporting standards may be more likely to be submitted to, and accepted by, English-language journals. Additionally, studies published in English may more frequently involve international collaborations, which can contribute additional methodological expertise and promote stricter adherence to reporting standards.

Additionally, lower reporting quality was observed in studies whose first author was affiliated with a Peruvian institution, whereas higher reporting quality was found in studies with a larger number of authors and at least one internationally affiliated author. These findings may reflect the potential contribution of international collaboration to improving reporting standards in qualitative research involving Peruvian populations. Previous evidence suggests that international collaboration has a positive impact on research quality [24,25], particularly in less well-established research systems [26], such as Peru.

### Limitations and strengths

This study has some limitations. First, mixed-methods studies were not included, as their methodological quality is assessed using specific appraisal tools, such as the Mixed Methods Appraisal Tool (MMAT), which fall outside the scope of the present evaluation [27]. Second, the search was restricted to Scopus, excluding regional databases such as SciELO or LILACS due to their limitation in filtering results by affiliation in these repositories. Although Scopus allows the identification of studies with affiliation with Peruvian institutions, this restriction may have omitted qualitative research published in journals with a local scope. Third, the analysis was limited to the 2022–2025 period, to focus on recent studies and reduce potential bias related to the accelerated publication processes during the COVID-19 pandemic [28]. While this timeframe may have limited the inclusion of relevant scientific production, this scope allows for an evaluation of the current state of qualitative research in Peru. Fourth, although the SRQR provides a structured framework for evaluating reporting quality, the interpretation of some items may involve a degree of reviewer subjectivity. Also, because the SRQR evaluates reporting quality rather than intrinsic methodological rigor, some studies may have received higher or lower scores based on reporting completeness rather than the strength of their design. Finally, the regression analysis estimated crude associations; therefore, the observed relationships should be interpreted cautiously and should not be interpreted as evidence of causal relationships.

On the other hand, this study also has some strengths. First, flexibility was applied in the interpretation of certain SRQR items to avoid ratings being influenced by editorial decisions rather than the absence of relevant content. For instance, some articles did not present a structured abstract (assessed under item 2) yet contained all expected elements and were therefore awarded full points. Similarly, statements regarding reflexivity and ethical considerations located outside the methods section were accepted as valid. Second, study selection, data extraction, and analysis were conducted independently by pairs of reviewers, with discrepancies resolved by a third or even fourth evaluator, thereby reducing the risk of individual bias and strengthening the reliability of the assessment process. Third, for data extraction, each item was assigned a score, and this score was accompanied by a justification, which allowed for greater discussion among the authors and increased rigor in the assignment of the scores. Finally, a widely recognized instrument such as the SRQR was used, whose structure allows for the assessment of items applicable across multiple methodological approaches within qualitative research [29].

## Conclusion

This review identifies key areas for improvement in the reporting of qualitative health studies in the Peruvian population, including techniques to ensure trustworthiness and credibility, methodological approach, researcher reflexivity, title quality, and reporting of ethics approval. Editorial teams could encourage adherence to standardized reporting guidelines and make editorial policies more explicit regarding ethics committee approval and key elements of methodological transparency, thereby supporting improved reporting practices. Future research could investigate whether these challenges persist in other contexts with similar structural and geographical disparities.

## Supporting information

S1 Table Preferred Reporting Items for Systematic reviews and Meta-Analyses extension for Scoping Reviews (PRISMA-ScR) checklist. (DOCX)

S2 Table Characteristics of the included studies (n = 147). (DOCX)

S3 Table Table of excluded studies (n = 55). (DOCX)

S4 Table Extracted dataset. (XLSX)

## References

[1] Kuper A, Reeves S, Levinson W. An introduction to reading and appraising qualitative research. BMJ. 2008;337:a288. doi: 10.1136/bmj.a288 18687727

[2] Huston P, Rowan M. Qualitative studies. Their role in medical research. Can Fam Physician. 1998;44:2453–8. 9839063 PMC2277956

[3] Renjith V, Yesodharan R, Noronha JA, Ladd E, George A. Qualitative methods in health care research. Int J Prev Med. 2021;12:20. doi: 10.4103/ijpvm.IJPVM_321_19 34084317 PMC8106287

[4] Dunt D, McKenzie R. Improving the quality of qualitative studies: Do reporting guidelines have a place?. Fam Pract. 2012;29(4):367–9. doi: 10.1093/fampra/cms041 22850798

[5] Mays N, Pope C. Qualitative research in health care. Assessing quality in qualitative research. BMJ. 2000;320(7226):50–2. doi: 10.1136/bmj.320.7226.50 10617534 PMC1117321

[6] O’Brien BC, Harris IB, Beckman TJ, Reed DA, Cook DA. Standards for reporting qualitative research: A synthesis of recommendations. Acad Med. 2014;89(9):1245–51. doi: 10.1097/ACM.0000000000000388 24979285

[7] Tong A, Sainsbury P, Craig J. Consolidated criteria for reporting qualitative research (COREQ): A 32-item checklist for interviews and focus groups. Int J Qual Health Care. 2007;19(6):349–57. doi: 10.1093/intqhc/mzm042 17872937

[8] Tong A, Flemming K, McInnes E, Oliver S, Craig J. Enhancing transparency in reporting the synthesis of qualitative research: ENTREQ. BMC Med Res Methodol. 2012;12:181. doi: 10.1186/1471-2288-12-181 23185978 PMC3552766

[9] Aref HAT, Witry M, Olufemi-Yusuf D, Guirguis LM. Ensuring quality qualitative research reporting in community pharmacy: A systematic literature review. Int J Pharm Pract. 2021;29(5):416–27. doi: 10.1093/ijpp/riab027 34390342

[10] McCloskey K, Neuzil K, Basak R, Chan KH. Quality of reporting for qualitative studies in pediatric urology-A scoping review. J Pediatr Urol. 2023;19(5):643–51. doi: 10.1016/j.jpurol.2023.04.027 37481426

[11] Silva GS, Fernandes D de RF, Alves CRL. Avaliação da assistência à saúde da criança na Atenção Primária no Brasil: Revisão sistemática de métodos e resultados. Ciênc saúde coletiva. 2020;25(8):3185–200. doi: 10.1590/1413-81232020258.27512018 32785553

[12] Cardona-Arias JA, Salas-Zapata W, Carmona-Fonseca J. Systematic review of qualitative studies about malaria in Colombia. Heliyon. 2020;6(5):e03964. doi: 10.1016/j.heliyon.2020.e03964 32885059 PMC7452435

[13] Angarita FA, Zhang Y, Elmi M, Look Hong NJ. Older women’s experience with breast cancer treatment: A systematic review of qualitative literature. Breast. 2020;54:293–302. doi: 10.1016/j.breast.2020.11.009 33242756 PMC7695983

[14] Mendoza-Chuctaya G, Chachaima-Mar JE, Mejia CR, et al. Análisis de producción, impacto y redes de colaboración en investigaciones científicas en Scopus en Perú de 2000 a 2019. Medwave. 2021;21(2). doi: 10.5867/medwave.2021.02.8121 33830979

[15] Carcausto-Calla WH, Morales-Quispe J. Investigaciones cualitativas en salud publicadas en revistas biomédicas peruanas. An Fac Med. 2018;79(2):144–8. doi: 10.15381/anales.v79i2.14941

[16] Watson A, Jackson D. The importance of analytic integrity and reporting guidelines in reporting qualitative research. J Adv Nurs. 2025;81(7):3425–7. doi: 10.1111/jan.16617 39513647

[17] Tricco AC, Lillie E, Zarin W, O’Brien KK, Colquhoun H, Levac D, et al. PRISMA Extension for Scoping Reviews (PRISMA-ScR): Checklist and explanation. Ann Intern Med. 2018;169(7):467–73. doi: 10.7326/M18-0850 30178033

[18] Arcila-Diaz J, Delgado-Caramutti J, Millones-Gómez PA, Figueroa-Quiñones J, Valencia-Arias A. Research trends in Peruvian universities: Proposal for a research agenda with a bibliometric approach. Scientometrics. 2024;130(1):489–514. doi: 10.1007/s11192-024-05211-z

[19] Vásquez-Uriarte K, Roque-Henriquez JC, Angulo-Bazán Y, Ninatanta Ortiz JA. Análisis bibliométrico de la producción científica peruana sobre la COVID-19. Rev Peru Med Exp Salud Publica. 2021;38(2):224–31. doi: 10.17843/rpmesp.2021.382.7470 34468568

[20] Choquez-Millan MF, Lechtape CL, Löhr K, Schröter B, Graef F. Uncovering power asymmetries in North-South research collaborations – An example from sustainability research in Tanzania. Futures. 2024;156:103316. doi: 10.1016/j.futures.2023.103316

[21] Resolución ministerial n.° 233-2020-MINSA. https://www.gob.pe/institucion/minsa/normas-legales/541139-233-2020-minsa. Accessed 2026 January 8.

[22] Stockemer D, Wigginton MJ. Publishing in English or another language: An inclusive study of scholar’s language publication preferences in the natural, social and interdisciplinary sciences. Scientometrics. 2019;118(2):645–52. doi: 10.1007/s11192-018-2987-0

[23] Di Bitetti MS, Ferreras JA. Publish (in English) or perish: The effect on citation rate of using languages other than English in scientific publications. Ambio. 2017;46(1):121–7. doi: 10.1007/s13280-016-0820-7 27686730 PMC5226904

[24] Kohus Z, Demeter M, Szigeti GP, Kun L, Lukács E, Czakó K. The Influence of international collaboration on the scientific impact in V4 countries. Publications. 2022;10(4):35. doi: 10.3390/publications10040035

[25] Wang J, Frietsch R, Neuhäusler P, Hooi R. International collaboration leading to high citations: Global impact or home country effect?. Journal of Informetrics. 2024;18(4):101565. doi: 10.1016/j.joi.2024.101565

[26] Farias Borges LF, Fischer B, Mazoni A, Bascur JP. The complex interplay between international collaboration and scientific impact: Evidence from the Scopus database. Cogent Education. 2025;12(1). doi: 10.1080/2331186x.2025.2563708

[27] Hong QN, Fàbregues S, Bartlett G, et al. The Mixed Methods Appraisal Tool (MMAT) version 2018 for information professionals and researchers. Educ Inf. 2018;34(4):285–91. doi: 10.3233/EFI-180221

[28] McDermott KT, Perry M, Linden W, Croft R, Wolff R, Kleijnen J. The quality of COVID-19 systematic reviews during the coronavirus 2019 pandemic: An exploratory comparison. Syst Rev. 2024;13(1):126. doi: 10.1186/s13643-024-02552-x 38720337 PMC11077834

[29] Dossett LA, Kaji AH, Cochran A. SRQR and COREQ reporting guidelines for qualitative studies. JAMA Surg. 2021;156(9):875–6. doi: 10.1001/jamasurg.2021.0525 33825809

