# Supplementary material for: Reporting quality of qualitative health studies published by Peruvian authors: A scoping review
Source: PLoS One. 2026 Jun 23;21(6):e0351494. doi: 10.1371/journal.pone.0351494 (PMC13289893; doi:10.1371/journal.pone.0351494)
Supplement: S2 Table — (DOCX) [file pone.0351494.s002.docx]

**S2 Table.** Characteristics of the included studies (n=147)

| **Author (year)** | **Language** | **Published in a Peruvian journal** | **Number of authors** | **Country of the first author's first affiliation** | **International collaboration** | **Population group** | **Sample size** | **Study setting** | **Region where the study was conducted** | **Multicountry sample** | **Reported the ethics committee approval** | **Reported the ethics approval code** | **SRQR score (range: 0 - 42)** |
| --- | --- | --- | --- | --- | --- | --- | --- | --- | --- | --- | --- | --- | --- |
| (Ayala et al., 2024) | Spanish | No | 5 | Peru | No | Mothers, fathers, caregivers, and families | 10 | Community settings | Cajamarca | No | Yes | Yes | 35 |
| (Agulnik, Ferrara, et al., 2022) | English | No | 18 | United States of America | Yes | Health professionals (clinicians and care personnel) | 71 | Health facilities | Lima | Yes | Yes | No | 37 |
| (Agulnik, Schmidt-Grimminger, et al., 2022) | English | No | 20 | United States of America | Yes | Health professionals (clinicians and care personnel) | 71 | Health facilities | Lima | Yes | Yes | No | 36 |
| (Aldana-Heredia & Zamudio-Rojas, 2025) | English | No | 2 | Peru | No | Policy makers, administrators, program managers, community leaders, stakeholders, and other non-clinical populations | 9 | Others | Not reported | No | No | No | 26 |
| (Allen et al., 2022) | English | No | 5 | United States of America | Yes | Policy makers, administrators, program managers, community leaders, stakeholders, and other non-clinical populations | 53 | Community settings | Junín, Trujillo | No | Yes | No | 33 |
| (Al-Rousan et al., 2023) | English | No | 10 | United States of America | Yes | Health professionals (clinicians and care personnel) | 12 | Health facilities | Lima | Yes | Yes | Yes | 36 |
| (Amado-Tineo et al., 2024) | English | No | 4 | Peru | Yes | Mothers, fathers, caregivers, and families | 12 | Health facilities | Lima | No | Yes | Yes | 41 |
| (Arango et al., 2025) | English | No | 8 | United States of America | Yes | Patients, service users, and individuals living with health conditions | 85 | Health facilities | Lima | No | Yes | Yes | 35 |
| (Arroyo-Laguna & Aramburú, 2023) | Spanish | No | 2 | Peru | No | Policy makers, administrators, program managers, community leaders, stakeholders, and other non-clinical populations | 62 | Community settings | Lima | No | Yes | No | 26 |
| (Arévalo-Ipanaqué et al., 2023) | English | No | 3 | Peru | No | Health professionals (clinicians and care personnel) | 6 | Health facilities | Lima | No | Yes | Yes | 37 |
| (Ascorbe-Amaya et al., 2023) | Spanish | No | 6 | Peru | No | Mothers, fathers, caregivers, and families | 13 | Community settings | Lambayeque | No | Yes | Yes | 33 |
| (Chancafe et al., 2024) | Spanish | No | 6 | Peru | No | Patients, service users, and individuals living with health conditions | 12 | Health facilities | Lambayeque | No | Yes | Yes | 34 |
| (Baca-Romero et al., 2022) | Spanish | No | 5 | Peru | No | Policy makers, administrators, program managers, community leaders, stakeholders, and other non-clinical populations | 10 | Community settings | Lima, Loreto, La Libertad, Lambayeque, Piura | No | No | No | 21 |
| (Berlowitz et al., 2025) | English | No | 6 | Switzerland | Yes | Indigenous peoples, individuals from rural communities, and culturally specific groups | 13 | Community settings | Loreto, Ucayali, San Martín | No | Yes | Yes | 34 |
| (Bressan et al., 2022) | English | No | 9 | Canada | Yes | Policy makers, administrators, program managers, community leaders, stakeholders, and other non-clinical populations | 13 | Community settings | Loreto, Ucayali, Madre de Dios, Junin, Cusco, San Martin | No | Yes | Yes | 40 |
| (Brown et al., 2023) | English | No | 4 | United Kingdom | Yes | Policy makers, administrators, program managers, community leaders, stakeholders, and other non-clinical populations | 18 | Community settings | Not reported | No | Yes | Yes | 37 |
| (Cañari et al., 2023) | English | No | 4 | Peru | No | Patients, service users, and individuals living with health conditions | 4 | Community settings | Not reported | Yes | Yes | Yes | 28 |
| (Calle-Chumacero et al., 2024) | Spanish | No | 4 | Peru | No | Children, adolescents, and young people | 16 | Others | Lima | No | No | No | 29 |
| (Camizan García et al., 2025) | English and Spanish | No | 6 | Peru | No | Patients, service users, and individuals living with health conditions | 12 | Health facilities | Lambayeque | No | Yes | Yes | 33 |
| (Carney et al., 2024) | English | No | 5 | United States of America | Yes | Health professionals (clinicians and care personnel) | 25 | Community settings | Lima | No | Yes | No | 36 |
| (Carroll et al., 2025) | English | No | 5 | United States of America | Yes | Indigenous peoples, individuals from rural communities, and culturally specific groups | 35 | Community settings | Lima | No | Yes | Yes | 30 |
| (Ramos Castro, 2022) | Spanish | No | 1 | Peru | No | Patients, service users, and individuals living with health conditions | 12 | Health facilities | Lambayeque | No | Yes | No | 31 |
| (Cavero et al., 2022) | English | No | 11 | Peru | Yes | Health professionals (clinicians and care personnel) | 45 | Others | Lima | Yes | Yes | No | 39 |
| (Oliden Chavez et al., 2023) | Spanish | No | 3 | Peru | No | Health professionals (clinicians and care personnel) | 12 | Health facilities | Lambayeque | No | Yes | Yes | 32 |
| (Chiang et al., 2023) | English | No | 10 | United States of America | Yes | Patients, service users, and individuals living with health conditions | 83 | Others | Lima | No | Yes | Yes | 39 |
| (Chicmana-Zapata et al., 2023) | English | No | 19 | Peru | Yes | Indigenous peoples, individuals from rural communities, and culturally specific groups | 18 | Community settings | Junín, Loreto | No | Yes | Yes | 38 |
| (Cuba-Martínez et al., 2023) | Spanish | No | 3 | Peru | No | Children, adolescents, and young people | 12 | Community settings | Not reported | No | No | No | 35 |
| (Cuba-Sancho et al., 2023) | Spanish | No | 5 | Peru | Yes | Mothers, fathers, caregivers, and families | 11 | Community settings | Lima | No | Yes | Yes | 27 |
| (Cueto et al., 2024) | Spanish | Yes | 4 | Peru | No | Policy makers, administrators, program managers, community leaders, stakeholders, and other non-clinical populations | 13 | Community settings | Lima | No | No | No | 22 |
| (Cusihuaman-Lope et al., 2023) | English and Spanish | Yes | 5 | Peru | No | Mothers, fathers, caregivers, and families | 24 | Health facilities | Lima, Callao | No | Yes | Yes | 40 |
| (Rubio & Asenjo-Alarcón, 2023) | Spanish | No | 2 | Peru | No | Patients, service users, and individuals living with health conditions | 16 | Community settings | Cajamarca | No | Yes | Yes | 36 |
| (De la Cruz-Torralva et al., 2024) | Spanish | Yes | 8 | Peru | No | Health professionals (clinicians and care personnel) | 8 | Health facilities | Lima, Loreto, La Libertad, Junín, Piura | No | Yes | Yes | 32 |
| (Velásquez et al., 26) | Spanish | No | 6 | Peru | No | Mothers, fathers, caregivers, and families | 15 | Health facilities | Lambayeque | No | No | No | 35 |
| (Priego, 2023) | Spanish | No | 1 | Peru | No | Children, adolescents, and young people | 10 | Educational centers | Lima | No | No | No | 32 |
| (Diez-Canseco et al., 2024) | English | No | 7 | Peru | Yes | Mothers, fathers, caregivers, and families | 98 | Community settings | Lima, Piura, Ayacucho, San Martín | No | Yes | Yes | 36 |
| (Escalante et al., 2023) | English | No | 5 | Peru | No | Policy makers, administrators, program managers, community leaders, stakeholders, and other non-clinical populations | 9 | Educational centers | Not reported | No | No | No | 14 |
| (Domínguez-Vergara et al., 2024) | English and Spanish | No | 4 | Peru | No | Policy makers, administrators, program managers, community leaders, stakeholders, and other non-clinical populations | 26 | Health facilities | Lima | No | Yes | Yes | 37 |
| (Quispe-Mamani et al., 2022) | Spanish | No | 5 | Peru | No | Policy makers, administrators, program managers, community leaders, stakeholders, and other non-clinical populations | 16 | Others | Puno | No | No | No | 23 |
| (Fhon et al., 2023) | English and Spanish | No | 8 | Brazil | Yes | Health professionals (clinicians and care personnel) | 47 | Health facilities | Lima, La Libertad | No | Yes | Yes | 34 |
| (Flores et al., 2025) | English | No | 7 | United Kingdom | Yes | Mothers, fathers, caregivers, and families | 48 | Community settings | Piura, Puno, Ucayali, Ayacucho | No | Yes | Yes | 40 |
| (Galea et al., 2022) | English | No | 15 | United States of America | Yes | Patients, service users, and individuals living with health conditions | 26 | Health facilities | Lima | No | Yes | No | 36 |
| (Heredia Guevara et al., 2022) | Spanish | No | 3 | Peru | No | Health professionals (clinicians and care personnel) | 12 | Health facilities | Lambayeque | No | Yes | Yes | 34 |
| (Guillén Zambrano et al., 2024) | English | No | 3 | Peru | No | Children, adolescents, and young people | 32 | Educational centers | Lima, Cusco, San Martín | No | No | No | 28 |
| (Heredia-Ampudia et al., 2025) | Spanish | No | 5 | Peru | No | Health professionals (clinicians and care personnel) | 15 | Health facilities | Lima | No | Yes | Yes | 31 |
| (Hernandez et al., 2023) | Spanish | No | 6 | Peru | No | Health professionals (clinicians and care personnel) | 15 | Health facilities | Lambayeque | No | Yes | Yes | 33 |
| (Herrera et al., 2023) | English | No | 4 | Peru | No | Policy makers, administrators, program managers, community leaders, stakeholders, and other non-clinical populations | 14 | Educational centers | Lima | No | Yes | No | 31 |
| (Hinckley et al., 2025) | English | No | 13 | Switzerland | Yes | Mothers, fathers, caregivers, and families | 25 | Community settings | Cajamarca | No | Yes | Yes | 40 |
| (Huaraz-Gutierrez et al., 2025) | Spanish | Yes | 3 | Peru | No | Indigenous peoples, individuals from rural communities, and culturally specific groups | 14 | Community settings | Junin | No | Yes | No | 33 |
| (Gutierrez et al., 2022) | Spanish | No | 6 | Peru | No | Health professionals (clinicians and care personnel) | 15 | Health facilities | Amazonas | No | No | No | 34 |
| (Jara-Avellaneda et al., 2023) | Spanish | No | 4 | Peru | No | Children, adolescents, and young people | 10 | Educational centers | Not reported | No | No | No | 29 |
| (Jaramillo et al., 2025) | English | No | 13 | Colombia | Yes | Children, adolescents, and young people | 112 | Community settings | Lima | Yes | Yes | Yes | 42 |
| (Jimenez Sanchez et al., 2025) | English | No | 10 | Peru | Yes | Children, adolescents, and young people | 25 | Health facilities | Lima | No | Yes | Yes | 41 |
| (Julca-Chilcon et al., 2022) | Spanish | No | 4 | Peru | No | Health professionals (clinicians and care personnel) | 20 | Health facilities | Lambayeque | No | Yes | Yes | 34 |
| (Juárez-Chávez et al., 2025) | English | No | 5 | Peru | No | Indigenous peoples, individuals from rural communities, and culturally specific groups | 19 | Health facilities | Lima | No | Yes | Yes | 41 |
| (Chumbiauca et al., 2022) | Spanish | Yes | 14 | Peru | No | Children, adolescents, and young people | 13 | Others | Lima | No | No | No | 28 |
| (Lai et al., 2022) | English | No | 7 | United Kingdom | Yes | Health professionals (clinicians and care personnel) | 21 | Health facilities | Loreto | No | Yes | No | 42 |
| (Lankowski et al., 2024) | English | No | 8 | United States of America | Yes | Indigenous peoples, individuals from rural communities, and culturally specific groups | 26 | Community settings | Lima | No | Yes | Yes | 40 |
| (Lazo-Gonzales et al., 2023) | English | No | 5 | Peru | Yes | Indigenous peoples, individuals from rural communities, and culturally specific groups | 60 | Community settings | Junin | No | Yes | Yes | 39 |
| (Pérez et al., 2024) | Spanish | No | 5 | Peru | No | Mothers, fathers, caregivers, and families | 29 | Others | Not reported | No | No | No | 23 |
| (Levey et al., 2023) | English | No | 7 | United States of America | Yes | Mothers, fathers, caregivers, and families | 43 | Health facilities | Lima | No | Yes | No | 40 |
| (Levey et al., 2024) | English | No | 8 | United States of America | Yes | Mothers, fathers, caregivers, and families | 43 | Health facilities | Lima | No | Yes | No | 40 |
| (Leyva-Moral et al., 2025) | English | No | 6 | Spain | Yes | Children, adolescents, and young people | 17 | Educational centers | Lima | No | Yes | Yes | 41 |
| (Linares-Olano et al., 2023) | Spanish | No | 6 | Peru | No | Health professionals (clinicians and care personnel) | 27 | Health facilities | Lima | No | Yes | Yes | 38 |
| (Lizarraga et al., 2025) | English | No | 9 | United States of America | Yes | Children, adolescents, and young people | 12 | Health facilities | Arequipa, Lima | Yes | Yes | Yes | 41 |
| (Llanos-Zavalaga et al., 2023) | Spanish | Yes | 2 | Peru | No | Policy makers, administrators, program managers, community leaders, stakeholders, and other non-clinical populations |  | Others | Lima | No | Yes | Yes | 32 |
| (Lovera Anyosa et al., 2025) | English | No | 5 | Peru | No | Policy makers, administrators, program managers, community leaders, stakeholders, and other non-clinical populations | 10 | Others | Lima | No | No | No | 28 |
| (Merino Lozano et al., 2024) | Spanish | No | 6 | Peru | No | Health professionals (clinicians and care personnel) | 13 | Others | Not reported | No | No | No | 32 |
| (Castillejo Rodriguez et al., 2022) | Spanish | No | 6 | Peru | No | Mothers, fathers, caregivers, and families | 17 | Community settings | Lima | No | No | No | 33 |
| (Manrique et al., 2023) | Spanish | No | 3 | Peru | No | Policy makers, administrators, program managers, community leaders, stakeholders, and other non-clinical populations | 5 | Health facilities | Lima | No | No | No | 32 |
| (Maquera Maquera, Bermejo Paredes, et al., 2024) | Spanish | No | 8 | Peru | No | Policy makers, administrators, program managers, community leaders, stakeholders, and other non-clinical populations | 15 | Educational centers | Puno | No | No | No | 26 |
| (Maquera Maquera et al., 2025) | Spanish | No | 7 | Peru | No | Policy makers, administrators, program managers, community leaders, stakeholders, and other non-clinical populations | 14 | Educational centers | Puno | No | No | No | 26 |
| (Maquera Maquera, Olivera Condori, et al., 2024) | Spanish | No | 4 | Peru | No | Policy makers, administrators, program managers, community leaders, stakeholders, and other non-clinical populations | 12 | Educational centers | Puno | No | No | No | 25 |
| (Maquera-Maquera et al., 2025) | Spanish | No | 4 | Peru | No | Policy makers, administrators, program managers, community leaders, stakeholders, and other non-clinical populations | 14 | Educational centers | Puno | No | No | No | 23 |
| (Marcelo et al., 2024) | English and Spanish | No | 3 | Peru | Yes | Patients, service users, and individuals living with health conditions | 32 | Community settings | Lambayeque | No | Yes | Yes | 35 |
| (Becerra et al., 2022) | Spanish | Yes | 13 | Peru | No | Patients, service users, and individuals living with health conditions | 11 | Health facilities | Lima | No | Yes | No | 39 |
| (Mayo-Puchoc et al., 2023) | English | No | 9 | Perú | Yes | Health professionals (clinicians and care personnel) | 20 | Health facilities | Lima | No | Yes | Yes | 39 |
| (Cervera Vallejos et al., 2024) | Spanish | No | 6 | Peru | No | Mothers, fathers, caregivers, and families | 15 | Community settings | Lambayeque | No | Yes | Yes | 37 |
| (Meneses-La-Riva et al., 2025) | English | No | 5 | Peru | No | Health professionals (clinicians and care personnel) | 9 | Health facilities | Lima | No | Yes | Yes | 39 |
| (Agip & Castillo, 2024) | Spanish | No | 2 | Peru | No | Policy makers, administrators, program managers, community leaders, stakeholders, and other non-clinical populations | 20 | Others | Lima | No | No | No | 26 |
| (Kamichi Miyashiro, 2022) | Spanish | No | 1 | Peru | No | Health professionals (clinicians and care personnel) | 3 | Community settings | Not reported | No | No | No | 11 |
| (Perez Mogrovejo et al., 2024) | Spanish | Yes | 5 | Peru | No | Health professionals (clinicians and care personnel) | 15 | Health facilities | Lima, Callao | No | Yes | Yes | 32 |
| (Monteagudo et al., 2025) | English | No | 5 | Spain | Yes | Children, adolescents, and young people | 160 | Educational centers | Not reported | Yes | Yes | Yes | 41 |
| (Morse et al., 2022) | English | No | 34 | United States of America | Yes | Patients, service users, and individuals living with health conditions | 47 | Health facilities | Loreto | No | Yes | Yes | 40 |
| (Morse et al., 2023) | English | No | 36 | United States of America | Yes | Patients, service users, and individuals living with health conditions | 20 | Others | Loreto | No | Yes | Yes | 40 |
| (Moya-Salazar et al., 2023) | English | No | 5 | Peru | No | Mothers, fathers, caregivers, and families | 15 | Others | Not reported | No | Yes | Yes | 36 |
| (Moya-Salazar et al., 2022) | English | No | 6 | Peru | No | Mothers, fathers, caregivers, and families | 15 | Others | Lima, Arequipa, Ayacucho, Pasco | No | Yes | Yes | 38 |
| (Mogollón Torres et al., 2024) | Spanish | No | 6 | Peru | No | Mothers, fathers, caregivers, and families | 12 | Health facilities | Lambayeque, Cajamarca, Amazonas | No | Yes | Yes | 36 |
| (Navarro-Ordinola et al., 2024) | Spanish | No | 4 | Peru | No | Health professionals (clinicians and care personnel) | 10 | Health facilities | Lambayeque | No | Yes | Yes | 34 |
| (Naz-McLean et al., 2024) | English | No | 7 | Canada | Yes | Indigenous peoples, individuals from rural communities, and culturally specific groups | 34 | Community settings | Lima | No | Yes | Yes | 38 |
| (Naz-McLean et al., 2022) | English | No | 10 | United States of America | Yes | Indigenous peoples, individuals from rural communities, and culturally specific groups | 20 | Community settings | Lima | No | Yes | Yes | 39 |
| (Ramón & Giove, 2023) | Spanish | Yes | 2 | Peru | No | Policy makers, administrators, program managers, community leaders, stakeholders, and other non-clinical populations |  | Others | San Martin | No | No | No | 33 |
| (Nunez et al., 2025) | English | No | 7 | United States of America | Yes | Patients, service users, and individuals living with health conditions | 110 | Community settings | Lima | No | Yes | Yes | 39 |
| (Ochoa-Panaifo et al., 2024) | Spanish | No | 3 | Peru | No | Patients, service users, and individuals living with health conditions | 8 | Others | Lima, Lambayeque, San Martin, La Libertad, Junin | No | Yes | Yes | 33 |
| (Oliva Rapoport et al., 2022) | English | No | 10 | United States of America | Yes | Patients, service users, and individuals living with health conditions | 85 | Others | Lima | No | Yes | Yes | 39 |
| (Ordoñez Espinoza et al., 2022) | Spanish | No | 4 | Peru | No | Patients, service users, and individuals living with health conditions | 12 | Health facilities | Lima | No | Yes | No | 30 |
| (Orozco-Poore et al., 2024) | English | No | 9 | United States of America | Yes | Indigenous peoples, individuals from rural communities, and culturally specific groups | 17 | Community settings | Lima | No | Yes | Yes | 37 |
| (Polo Campos et al., 2022) | Spanish | No | 6 | Peru | No | Mothers, fathers, caregivers, and families | 16 | Community settings | Lambayeque | No | Yes | Yes | 31 |
| (Paredes Ajalla et al., 2022) | Spanish | Yes | 2 | Peru | No | Patients, service users, and individuals living with health conditions | 25 | Health facilities | Lima | No | No | No | 28 |
| (Paredes-Angeles et al., 2024) | English | No | 6 | Peru | No | Policy makers, administrators, program managers, community leaders, stakeholders, and other non-clinical populations | 49 | Health facilities | Lima, Callao | No | Yes | Yes | 38 |
| (Pease Dreibelbis et al., 2024) | English | No | 3 | Peru | No | Children, adolescents, and young people | 66 | Educational centers | Lima, Cusco, San Martín | No | Yes | No | 34 |
| (Pedersen et al., 2023) | English | No | 12 | United States of America | Yes | Health professionals (clinicians and care personnel) | 27 | Others | Not reported | Yes | Yes | Yes | 36 |
| (Perez-Lluncor et al., 2022) | Spanish | No | 3 | Peru | No | Children, adolescents, and young people | 14 | Educational centers | Lambayeque | No | Yes | Yes | 31 |
| (Jesús et al., 2024) | Spanish | No | 3 | Mexico | Yes | Indigenous peoples, individuals from rural communities, and culturally specific groups | 14 | Community settings | Not reported | No | Yes | Yes | 26 |
| (Puicón-Mejía et al., 2024) | Spanish | No | 3 | Peru | No | Children, adolescents, and young people | 15 | Educational centers | Lambayeque | No | Yes | Yes | 25 |
| (Reisner et al., 2023) | English | No | 7 | United States of America | Yes | Indigenous peoples, individuals from rural communities, and culturally specific groups | 21 | Not reported | Not reported | No | Yes | No | 35 |
| (Rivas-Chapoñan et al., 2022) | Spanish | No | 3 | Peru | Yes | Health professionals (clinicians and care personnel) | 10 | Health facilities | Lambayeque | No | Yes | Yes | 36 |
| (Rivera-Cruzatt et al., 2022) | English | Yes | 3 | Peru | No | Patients, service users, and individuals living with health conditions | 16 | Health facilities | Lima | No | Yes | Yes | 37 |
| (Rivera-Miranda Giral et al., 2024) | English | No | 3 | Peru | No | Patients, service users, and individuals living with health conditions | 10 | Health facilities | Lima, Piura | No | Yes | No | 39 |
| (Mamani et al., 2023) | Spanish | No | 3 | Peru | No | Health professionals (clinicians and care personnel) | 10 | Health facilities | Lambayeque | No | No | No | 32 |
| (Flores Rojas & Tello Pompa, 2024) | Spanish | Yes | 3 | Peru | No | Indigenous peoples, individuals from rural communities, and culturally specific groups | 5 | Community settings | Cajamarca | No | No | No | 19 |
| (Rozas & Busse, 2022) | English | No | 2 | Peru | No | Indigenous peoples, individuals from rural communities, and culturally specific groups | 44 | Educational centers | Lima | No | Yes | Yes | 38 |
| (Safary et al., 2024) | English | No | 13 | Switzerland | Yes | Patients, service users, and individuals living with health conditions | 383 | Community settings | Lima | Yes | Yes | Yes | 39 |
| (Salinas-Gutierrez et al., 2024) | Spanish | No | 6 | Peru | No | Health professionals (clinicians and care personnel) | 12 | Health facilities | Lambayeque | No | Yes | Yes | 37 |
| (Tamayo et al., 2024) | Spanish | No | 3 | Peru | Yes | Health professionals (clinicians and care personnel) | 21 | Health facilities | La Libertad | No | Yes | Yes | 33 |
| (Falcón et al., 2022) | Spanish | No | 7 | Peru | No | Children, adolescents, and young people | 15 | Educational centers | Lima | No | Yes | Yes | 32 |
| (Sedano et al., 2022) | Spanish | Yes | 4 | Peru | No | Policy makers, administrators, program managers, community leaders, stakeholders, and other non-clinical populations | 15 | Educational centers | Junin | No | No | No | 20 |
| (Sánchez Chanamé et al., 2025) | Spanish | No | 6 | Peru | No | Children, adolescents, and young people | 12 | Educational centers | Lambayeque | No | Yes | Yes | 36 |
| (Sologuren-García et al., 2023) | English | No | 8 | Peru | No | Indigenous peoples, individuals from rural communities, and culturally specific groups | 7 | Community settings | Tacna | No | Yes | Yes | 40 |
| (Subileta-Yangali et al., 2025) | English | No | 3 | Peru | No | Indigenous peoples, individuals from rural communities, and culturally specific groups | 12 | Educational centers | Lima | No | No | No | 35 |
| (Córdova López et al., 2025) | English | Yes | 3 | Peru | No | Patients, service users, and individuals living with health conditions | 10 | Health facilities | Lambayeque | No | Yes | Yes | 40 |
| (Tapullima-Mori et al., 2024) | Spanish | Yes | 3 | Peru | No | Policy makers, administrators, program managers, community leaders, stakeholders, and other non-clinical populations | 6 | Educational centers | San Martin | No | No | No | 30 |
| (Tarazona-Meza et al., 2025) | English | No | 8 | Peru | Yes | Mothers, fathers, caregivers, and families | 36 | Community settings | Lima, Huánuco | No | Yes | Yes | 41 |
| (Tejada Muñoz et al., 2022) | Spanish | No | 5 | Peru | No | Children, adolescents, and young people | 14 | Community settings | Amazonas | No | No | No | 24 |
| (Tejada Muñoz et al., 2024) | Spanish | No | 6 | Peru | No | Children, adolescents, and young people | 12 | Community settings | Amazonas | No | No | No | 32 |
| (Temelkovska et al., 2023) | English | No | 9 | United States of America | Yes | Indigenous peoples, individuals from rural communities, and culturally specific groups | 20 | Community settings | Lima | No | Yes | Yes | 31 |
| (Tenorio-Mucha et al., 2022) | English and Spanish | Yes | 5 | Peru | Yes | Policy makers, administrators, program managers, community leaders, stakeholders, and other non-clinical populations | 30 | Community settings | Lima, Cusco, Ayacucho, Arequipa e Ica | No | Yes | Yes | 36 |
| (Torres-Fernandez et al., 2024) | Spanish | No | 5 | Peru | No | Policy makers, administrators, program managers, community leaders, stakeholders, and other non-clinical populations | 20 | Community settings | Lambayeque | No | Yes | Yes | 32 |
| (Torres-Slimming et al., 2023) | English and Spanish | No | 6 | Peru | Yes | Policy makers, administrators, program managers, community leaders, stakeholders, and other non-clinical populations | 59 | Community settings | Lima, Junín | No | Yes | Yes | 34 |
| (Toyama, Cavero, et al., 2022) | English and Spanish | No | 7 | Peru | Yes | Patients, service users, and individuals living with health conditions | 32 | Community settings | Lima | No | Yes | Yes | 34 |
| (Toyama, Godoy-Casasbuenas, et al., 2022) | English | No | 13 | Peru | Yes | Children, adolescents, and young people | 185 | Community settings | Lima | Yes | Yes | Yes | 36 |
| (Toyama et al., 2025) | English | No | 15 | Peru | Yes | Children, adolescents, and young people | 112 | Community settings | Lima | Yes | Yes | Yes | 34 |
| (Ubillús et al., 2023) | English and Spanish | Yes | 4 | Peru | No | Patients, service users, and individuals living with health conditions | 19 | Community settings | Lambayeque | No | Yes | Yes | 39 |
| (Ulco-Bravo et al., 2022) | Spanish | No | 5 | Peru | No | Mothers, fathers, caregivers, and families | 12 | Community settings | Lambayeque | No | Yes | Yes | 31 |
| (Uzátegui-Gamarra & Malvaceda-Espinoza, 2023) | English and Spanish | No | 2 | Peru | No | Mothers, fathers, caregivers, and families | 12 | Community settings | Lima | No | No | No | 30 |
| (Valenzuela Antezana et al., 2024) | Spanish | No | 5 | Peru | No | Policy makers, administrators, program managers, community leaders, stakeholders, and other non-clinical populations | 10 | Community settings | Lima | No | Yes | No | 32 |
| (VanDerWal et al., 2025) | English | No | 18 | United States of America | Yes | Policy makers, administrators, program managers, community leaders, stakeholders, and other non-clinical populations | 49 | Community settings | Lima | No | Yes | No | 35 |
| (Benites-Vargas et al., 2024) | English and Spanish | No | 3 | Peru | No | Health professionals (clinicians and care personnel) | 10 | Community settings | Lima | No | Yes | No | 20 |
| (Velázquez & Bravo, 2024) | English and Spanish | No | 2 | Peru | No | Indigenous peoples, individuals from rural communities, and culturally specific groups | 10 | Community settings | Ayacucho | No | Yes | Yes | 30 |
| (Lustig Vijay et al., 2024) | English | No | 4 | Peru | Yes | Indigenous peoples, individuals from rural communities, and culturally specific groups | 11 | Community settings | San Martin | No | Yes | Yes | 31 |
| (Williams et al., 2024) | English | No | 20 | United States of America | Yes | Health professionals (clinicians and care personnel) | 55 | Community settings | Puno | No | Yes | Yes | 38 |
| (Wilson et al., 2023) | English | No | 9 | United States of America | Yes | Health professionals (clinicians and care personnel) | 12 | Community settings | Lima | No | Yes | No | 31 |
| (Woodson, Garcia Saldivar, et al., 2024) | English | No | 7 | United States of America | Yes | Indigenous peoples, individuals from rural communities, and culturally specific groups | 41 | Community settings | Loreto | No | Yes | Yes | 36 |
| (Woodson, Saldivar, et al., 2024) | English | No | 9 | United States of America | Yes | Children, adolescents, and young people | 56 | Community settings | Loreto | No | No | No | 35 |
| (Yslado-Méndez et al., 2025) | English | No | 9 | Peru | No | Policy makers, administrators, program managers, community leaders, stakeholders, and other non-clinical populations | 34 | Community settings | Ancash | No | Yes | Yes | 39 |
| (Zafra-Tanaka et al., 2024) | English | No | 11 | Peru | Yes | Patients, service users, and individuals living with health conditions | 22 | Community settings | Lima | No | No | No | 32 |
| (Zafra-Tanaka et al., 2022) | English | No | 5 | Peru | Yes | Patients, service users, and individuals living with health conditions | 18 | Community settings | Lima | No | Yes | No | 34 |
| (Zuleta et al., 2023) | English | No | 4 | United Kingdom | Yes | Policy makers, administrators, program managers, community leaders, stakeholders, and other non-clinical populations | 11 | Community settings | Not reported | No | Yes | No | 32 |

**References:**

Agip, M., & Castillo, S. (2024). La dinámica del comercio ilegal de armas de fuego en Lima, Perú. *Criminalidad*, *66*(1), 11-23.

Agulnik, A., Ferrara, G., Puerto-Torres, M., Gillipelli, S. R., Elish, P., Muniz-Talavera, H., Gonzalez-Ruiz, A., Armenta, M., Barra, C., Diaz, R., Hernandez, C., Juárez Tobias, S., de Jesus Loeza, J., Mendez, A., Montalvo, E., Penafiel, E., Pineda, E., & Graetz, D. E. (2022). Assessment of Barriers and Enablers to Implementation of a Pediatric Early Warning System in Resource-Limited Settings. *JAMA Network Open*, *5*(3), e221547. https://doi.org/10.1001/jamanetworkopen.2022.1547

Agulnik, A., Schmidt-Grimminger, G., Ferrara, G., Puerto-Torres, M., Gillipelli, S. R., Elish, P., Muniz-Talavera, H., Gonzalez-Ruiz, A., Armenta, M., Barra, C., Diaz-Coronado, R., Hernandez, C., Juarez, S., Loeza, J. de J., Mendez, A., Montalvo, E., Penafiel, E., Pineda, E., Graetz, D. E., & McKay, V. (2022). Challenges to sustainability of pediatric early warning systems (PEWS) in low-resource hospitals in Latin America. *Frontiers in Health Services*, *2*, 1004805. https://doi.org/10.3389/frhs.2022.1004805

Aldana-Heredia, W. I., & Zamudio-Rojas, R. E. (2025). TERRITORIAL GOVERNANCE IN HEALTH IN PERU: CHALLENGES AND OPPORTUNITIES OF THE NATIONAL MULTISECTORAL POLICY 2025. *TPM – Testing, Psychometrics, Methodology in Applied Psychology*, *32*(S2 (2025): Posted 09 June), 1266-1280.

Allen, E. M., Frisancho, A., Llanten, C., Knep, M. E., & Van Skiba, M. J. (2022). Community Health Agents Advancing Women’s Empowerment: A Qualitative Data Analysis. *Journal of Community Health*, *47*(5), 806-813. https://doi.org/10.1007/s10900-022-01107-2

Al-Rousan, T., Awad, M., Amalia Pesantes, M., Kandula, N. R., Huffman, M. D., Jaime Miranda, J., Vidal-Perez, R., Dzudie, A., Anderson, C. A. M., & BP MONITOR Study Group. (2023). Healthcare provider’s perspectives on home blood pressure management in Peru and Cameroon: Findings from the BPMONITOR study. *Preventive Medicine Reports*, *33*, 102179. https://doi.org/10.1016/j.pmedr.2023.102179

Amado-Tineo, J., Oscanoa-Espinoza, T., Loli-Ponce, R., & Delgado-Guay, M. O. (2024). Experiences of caregivers of patients with noncancer diseases readmitted to an emergency department at the end of life. *BMC Palliative Care*, *23*(1), 265. https://doi.org/10.1186/s12904-024-01596-z

Arango, D., Cintron, C., Beckhorn, C. B., Wong, M., Senador, L., Altamirano, E., Lecca, L., & Chiang, S. S. (2025). *Care for adolescents with drug-susceptible pulmonary tuberculosis in Lima, Peru: A qualitative assessment*. https://doi.org/10.1136/bmjopen-2024-090707

Arévalo-Ipanaqué, J. M., Obando Zegarra, R., & Cabanillas Chávez, M. T. (2023). Experiences of the Nursing Professionals Hospitalized by Covid-19 in Peru: Dawning Every Day Meant one More Chance to Live. *SAGE Open Nursing*, *9*, 23779608231196844. https://doi.org/10.1177/23779608231196844

Arroyo-Laguna, J., & Aramburú, C. E. (2023). Patrones de conducta social de trabajadores informales durante eventos extremos: Lecciones de la vida social durante la pandemia de covid-19 en Lima, Perú. *Salud Colectiva*, *19*, e4494. https://doi.org/10.18294/sc.2023.4494

Ascorbe-Amaya, A. A., Manchay, R. J. D., Ramírez, A. S. V.-, Rodríguez-Cruz, L. D. D., Mogollón-Torres, F. de M., & Constantino-Facundo, F. (2023). Medidas preventivas y costumbres en las familias no contagiadas por la COVID-19 durante el confinamiento. *Cultura de los Cuidados*, *27*(65), 285-299. https://doi.org/10.14198/cuid.2023.65.22

Ayala, C. L. A., Manchay, R. J. D., Vallejos, M. F. C., Montenegro, J. M. N., & Cruz, L. D. R. (2024). Prácticas culturales para prevenir la COVID-19 en una comunidad indígena. *Cultura de los Cuidados*, *69*, 213-228. https://doi.org/10.14198/cuid.21967

Baca-Romero, D., Aiquipa-Tello, J. J., Barboza-Palomino, M., Ibañez-Montoya, J. B., & Oré-Pacheco, L. P. (2022). Impacto psicológico y estrategias de afrontamiento en adultos peruanos durante la pandemia del Covid-19. *Index de Enfermería*, 61-65. https://doi.org/10.58807/indexenferm20224955

Becerra, P. S., Eyzaguirre, S. M. M., Yapa, C. V., Chunga, A. C., Soto, A. S., Vera, I. M. N., Ordoñez, S. S., Román, M. G., Miranda, M. O., Soto-Becerra, P., Roca, L. Y. H., Maguiña, J. L., & Araujo-Castillo, R. V. (2022). Narrativas del donante de plasma convaleciente en EsSalud: Motivaciones, miedos, expectativas y experiencias. *Revista del Cuerpo Médico Hospital Nacional Almanzor Aguinaga Asenjo*, *15*(4), 541-548. https://doi.org/10.35434/rcmhnaaa.2022.154.1689

Benites-Vargas, Y. N., Calderon-Luyo, A. S., Chaname-Ampuero, E. V., Benites-Vargas, Y. N., Calderon-Luyo, A. S., & Chaname-Ampuero, E. V. (2024). Percepciones de los profesionales de enfermería en la prevención del sobrepeso y la obesidad infantil. *Index de Enfermería*, *33*(1). https://doi.org/10.58807/index%252520enferm16570

Berlowitz, I., Pesantes, M. A., Cárdenas Palacios, C., Martin-Soelch, C., Wolf, U., & Maake, C. (2025). Towards culturally inclusive healthcare in Peru: Mapping epistemic concepts in contemporary Indigenous Amazonian medicine-Traditional healers’ perspectives. *PLOS Global Public Health*, *5*(1), e0003912. https://doi.org/10.1371/journal.pgph.0003912

Bressan, T., Valdivia-Gago, A., Silvera-Ccallo, R. M., Llanos-Cuentas, A., Condor, D. F., Padilla-Huamantinco, P. G., Vilcarromero, S., Miranda, J. J., & Zavaleta-Cortijo, C. (2022). Challenges of design, implementation, acceptability, and potential for, biomedical technologies in the Peruvian Amazon. *International Journal for Equity in Health*, *21*(1), 183. https://doi.org/10.1186/s12939-022-01773-7

Brown, L. J., Turner, B. M., Cavero, V., & Flores, E. C. (2023). Gender and the environmental health agenda: A qualitative study of policy, academic, and advocacy perspectives in Peru. *The Journal of Climate Change and Health*, *10*, 100217. https://doi.org/10.1016/j.joclim.2023.100217

Calle-Chumacero, K., Tinoco-Ibáñez, V., Almeida-Huanca, G., & Malvaceda-Espinoza, E. (2024). Calidad de vida laboral en practicantes universitarios de Psicología. *Revista Brasileira de Orientação Profissional*, *25*(1), 41-52. https://doi.org/10.26707/1984-7270/2024v25n0105

Camizan García, J. M., Díaz -Manchay, R. J., Cervera-Vallejos, M. F., Rodríguez-Cruz, L. D., Huyhua-Gutierrez, S. C., & Tejada-Muñoz, S. (2025). Cuidados en el hogar en personas sujetas a hemodiálisis enfatizando la dieta y el acceso vascular. *Enfermería Nefrológica*, *28*(1), 10-16. https://doi.org/10.37551/S2254-28842025002

Cañari, B., Moya-Salazar, J., Bussalleu, D., & Contreras-Pulache, H. (2023). Does the use of cysteine-rich whey protein supplements (Inmunocal®) improve the health well-being of COVID-19 patients? A qualitative study. *Electronic Journal of General Medicine*, *20*(1), em433. https://doi.org/10.29333/ejgm/12652

Carney, J. R., Gallo, P. A., Espinoza, V., Yataco, L., & Miller-Graff, L. E. (2024). Supporting Accessible Care for Pregnant Women Experiencing IPV in Peru: A Thematic Analysis of Barriers to and Facilitators of Women’s Help Seeking. *Journal of Family Violence*, *39*(4), 581-593. https://doi.org/10.1007/s10896-023-00526-6

Carroll, H. A., Guevara, T., Aron, V., Freier, L. F., & Bird, M. D. (2025). «Pa’lante!» Perseverance, progress and mental health among venezuelan migrants in Peru: A qualitative study. *Journal of Migration and Health*, *11*, 100336. https://doi.org/10.1016/j.jmh.2025.100336

Castillejo Rodriguez, K., Arenas Iparraguirre, C., Malvaceda Espinoza, E., Reinstein Romero, S., Segovia Roque, M., & Sayán Cortéz, S. (2022). Experiencias de afrontamiento psicosocial en el contexto de aislamiento social obligatorio, en jefes de familia de Lima Metropolitana. *Cultura de los cuidados: Revista de Enfermería y Humanidades*, *64*, 5-18.

Cavero, V., Toyama, M., Castro, H., Couto, M. T., Brandt, L., Quayle, J., Menezes, P. R., Mohr, D. C., Araya, R., Miranda, J. J., & Diez-Canseco, F. (2022). Implementation and scalability of a digital intervention to reduce depressive symptoms in people with diabetes, hypertension or both in Brazil and Peru: A qualitative study of health system’s stakeholders’ perspectives. *Discover Mental Health*, *2*(1), 12. https://doi.org/10.1007/s44192-022-00015-0

Cervera Vallejos, M. F., Tejada Muñoz, S., Mejía Goicochea, D. C., Díaz Manchay, R., Vega Ramírez, A. S., & Mogollón Torres, F. de M. (2024). Cuidado domiciliario a personas con oxigenoterapia por COVID-19 según la experiencia del familiar cuidador. *Cultura de los cuidados: Revista de Enfermería y Humanidades*, *69*, 229-243.

Chancafe, J. M. B., Ramírez, A. S. V., Manchay, R. J. D., Torres, F. D. M. M., Vallejos, M. F. C., & Quiroz, E. S. G. (2024). El cuidado de Enfermería desde la percepción de las personas con VIH/sida. *Revista Cubana de Enfermería*, *40*. https://revenfermeria.sld.cu/index.php/enf/article/view/6085

Chiang, S. S., Senador, L., Altamirano, E., Wong, M., Beckhorn, C. B., Roche, S., Coit, J., Oliva Rapoport, V. E., Lecca, L., & Galea, J. T. (2023). Adolescent, caregiver and provider perspectives on tuberculosis treatment adherence: A qualitative study from Lima, Peru. *BMJ Open*, *13*(5), e069938. https://doi.org/10.1136/bmjopen-2022-069938

Chicmana-Zapata, V., Arotoma-Rojas, I., Anza-Ramírez, C., Ford, J., Galappaththi, E. K., Pickering, K., Sacks, E., Togarepi, C., Perera, C. D., van Bavel, B., Hyams, K., Akugre, F. A., Nkalubo, J., Dharmasiri, I., Nakwafila, O., Mensah, A., Miranda, J. J., & Zavaleta-Cortijo, C. (2023). Justice implications of health and food security policies for Indigenous peoples facing COVID-19: A qualitative study and policy analysis in Peru. *Health Policy and Planning*, *38*(Supplement_2), ii36-ii50. https://doi.org/10.1093/heapol/czad051

Chumbiauca, K. S., García, L. E. A., Pujay, E. C., Rivero, C. A. C., Calvo, G. K. C., Espinoza, R. N. C., Solís, M. C., Colan, M. F. C., Guidotti, J. E., Urdanivia, S. M. N., Azañedo, I. D. R., Polo, P. M. R., Silva, L. R., & Yachachín, K. N. V. (2022). Experiencias subjetivas sobre las clases virtuales en universitarios peruanos en tiempos de COVID 19. *PsiqueMag*, *11*(2), 117-127. https://doi.org/10.18050/psiquemag.v11i2.2116

Córdova López, E. T., Loayza-Enríquez, B. K., León Jiménez, F. E., Córdova López, E. T., Loayza-Enríquez, B. K., & León Jiménez, F. E. (2025). Myths and beliefs about diabetic foot: A cultural study in outpatients with type 2 diabetes at the Hospital Regional de Lambayeque. *Revista Medica Herediana*, *36*(1), 26-36. https://doi.org/10.20453/rmh.v36i1.5439

Cuba-Martínez, C. de L., Malvaceda-Espinoza, E., & Bossio, M. R.-. (2023). Experiencias de afrontamiento psicosocial frente a partidos internacionales en voleibolistas profesionales de la selección nacional del Perú. *Cuadernos de Psicología del Deporte*, *23*(1), 89-102. https://doi.org/10.6018/cpd.512421

Cuba-Sancho, J. M., Vivas Durand, T. de J., Eliete Batista, M., Zeladita-Huaman, J. A., Huamán Salazar, N., Cuba-Sancho, J. M., Vivas Durand, T. de J., Eliete Batista, M., Zeladita-Huaman, J. A., & Huamán Salazar, N. (2023). Representaciones sociales del desastre natural para las madres damnificadas de una comunidad urbano-marginal de Lima, Perú. *Revista Cubana de Enfermería*, *39*. http://scielo.sld.cu/scielo.php?script=sci_abstract&pid=S0864-03192023000100024&lng=es&nrm=iso&tlng=es

Cueto, R. M., Ayma, L., Llanco, C., & Espinosa, A. (2024). Significados y efectos del voluntariado en los Juegos Panamericanos y Parapanamericanos Lima 2019 y el Proyecto Especial Legado. *Revista de Psicología*, *42*(2), 1061-1096. https://doi.org/10.18800/psico.202402.014

Cusihuaman-Lope, N., Vilela-Estrada, A. L., Cavero, V., Villarreal-Zegarra, D., & Diez-Canseco, F. (2023). Experiencias de usuarios y familiares sobre la atención recibida en centros de salud mental comunitaria de Lima y Callao durante la pandemia de la COVID-19. *Revista Peruana de Medicina Experimental y Salud Pública*, 278-286. https://doi.org/10.17843/rpmesp.2023.403.12717

De la Cruz-Torralva, K., Escobar-Agreda, S., Pampa-Espinoza, L., Silva-Valencia, J., Reategui-Rivera, M., Chahuara-Rojas, M., Rojas-Mezarina, L., Cabezas-Sánchez, C., De la Cruz-Torralva, K., Escobar-Agreda, S., Pampa-Espinoza, L., Silva-Valencia, J., Reategui-Rivera, M., Chahuara-Rojas, M., Rojas-Mezarina, L., & Cabezas-Sánchez, C. (2024). Percepción de médicos especialistas sobre la usabilidad de una aplicación móvil de dengue en zonas rurales: Un estudio cualitativo. *Anales de la Facultad de Medicina*, *85*(1), 14-20. https://doi.org/10.15381/anales.v85i1.26175

Diez-Canseco, F., Najarro, L., Cavero, V., Saavedra-Garcia, L., Taillie, L. S., Carpentier, F. R. D., & Miranda, J. J. (2024). Recall, understanding, use, and impact of front-of-package warning labels on ultra-processed foods: A qualitative study with mothers of preschool children in Peru. *PLOS Global Public Health*, *4*(12), e0003938. https://doi.org/10.1371/journal.pgph.0003938

Domínguez-Vergara, J., Santa-Cruz-Espinoza, H., Torres-Villanueva, G. N., & Cabos Zelada, E. F. (2024). [Operational domains of healthy aging: A qualitative description in older adults in Peru]. *Revista Espanola De Geriatria Y Gerontologia*, *59*(4), 101485. https://doi.org/10.1016/j.regg.2024.101485

Escalante, E. R. D., Garay, J. P. P., Oliver, J. M. Z., Orbegoso, C. O. V., & Nuñez, E. F. D. (2023). Post-pandemic prevention strategies for burnout syndrome in Peruvian university teachers. *Salud, Ciencia y Tecnología*, *3*, 572-572. https://doi.org/10.56294/saludcyt2023572

Falcón, G. C. S., Moncada, M. J. A., Rojas, T. C. S., Arias, G. F. G., Camacho, C. V. C., Correa, M. I. del M. M., & Lluncor, E. M. L. (2022). Preventing COVID-19 as a nursing student through social networks in their family-social environment. *Revista Brasileira de Enfermagem*, *75*, e20210631. https://doi.org/10.1590/0034-7167-2021-0631

Fhon, J. R. S., Arpasi-Quispe, O., Zegarra, G. S. C., Neto, A. P. dos S., Gomez-Lujan, M. del P., Cueva, K. F. F., Araujo, T. L. D., & Leitón-Espinoza, Z. E. (2023). PERCEPCIÓN DEL ENFERMERO PERUANO EN EL CUIDADO DEL PACIENTE CON COVID-19. *Revista Baiana de Enfermagem‏*, *37*. https://doi.org/10.18471/rbe.v37.52052

Flores, E. C., Flores, A. F., Abarca-Diaz, B., Camizan-Castro, R., Mendoza-Lozada, E., Ortiz-Contreras, F., & Kakuma, R. (2025). Fostering leadership and gender equality in climate action among underserved, rural and Indigenous women: A qualitative exploration of opportunities and limitations in Peru. *Lancet Regional Health. Americas*, *46*, 101109. https://doi.org/10.1016/j.lana.2025.101109

Flores Rojas, E. S., & Tello Pompa, C. A. (2024). *Enfermería y necesidades colectivas en familias rurales, Chalamarca (Chota, Perú), 2024. | EBSCOhost*. https://openurl.ebsco.com/EPDB%3Agcd%3A10%3A35039402/detailv2?sid=ebsco%3Aplink%3Ascholar&id=ebsco%3Agcd%3A186513649&crl=c&link_origin=scholar.google.com

Galea, J. T., Wong, M., Ninesling, B., Ramos, A., Senador, L., Sanchez, H., Kolevic, L., Matos, E., Sanchez, E., Errea, R. A., Lindeborg, A., Benites, C., Lecca, L., Shin, S., & Franke, M. F. (2022). Patient and provider perceptions of a community-based accompaniment intervention for adolescents transitioning to adult HIV care in urban Peru: A qualitative analysis. *Journal of the International AIDS Society*, *25*(10), e26019. https://doi.org/10.1002/jia2.26019

Guillén Zambrano, H. R., de la Puente Ronceros, R. A., & Pease Dreibelbis, M. A. (2024). The subjective well-being in Peruvian adolescence: Identifying domains and evaluations in a non-WEIRD context. *Journal of Research on Adolescence: The Official Journal of the Society for Research on Adolescence*, *34*(2), 466-476. https://doi.org/10.1111/jora.12909

Gutierrez, S. C. H., Zumaeta, L. M. M., Alvites, S. V. R., Rojas, L. Z. S., Manchay, R. J. D., & Muñoz, S. T. (2022). Sentimientos de los profesionales de enfermería frente a la COVID-19. *Revista Cubana de Enfermería*, *38*. https://revenfermeria.sld.cu/index.php/enf/article/view/5094

Heredia Guevara, Z. T., Cervera Vallejos, M. F., & Diaz Manchay, R. J. (2022). DESAFÍOS ÉTICO-LEGALES EN EL CUIDADO AL PACIENTE EN EL SERVICIO DE URGENCIA: PERSPECTIVA DE LAS ENFERMERAS. *Ciencia y enfermería*, *28*. https://doi.org/10.29393/ce28-7dezr30007

Heredia-Ampudia, L., Palomino-Zarate, M., Mamani-Benito, O., Carranza-Esteban, R., & Malvaceda-Espinoza, E. (2025). Comunicación interna entre enfermeras de un hospital de Lima Metropolitana que realizan teletrabajo en el contexto de pandemia. *Revista Cubana de Información en Ciencias de la Salud*, *36*. https://acimed.sld.cu/index.php/acimed/article/view/2645

Hernandez, A. K. M., Manchay, R. J. D., Vallejos, M. F. C., Cruz, L. R., Tenorio, M. A., & Quiroz, S. G. (2023). Experiencias de las enfermeras no infectadas con COVID-19 que trabajaron durante el confinamiento. *Revista Cubana de Enfermería*, *39*. https://revenfermeria.sld.cu/index.php/enf/article/view/5853

Herrera, D., Iberico, C., Matos, L., & Cerna, Y. (2023). Professors’ motivational styles, future orientation, and engagement: A qualitative study in professor–student dyads in the school of music. *Psychology of Music*, *51*(4), 1243-1258. https://doi.org/10.1177/03057356221135348

Hinckley, K., Gastiaburú Cabello, M. C., Alvarado, M., McCoy, D. C., Mäusezahl, D., Fink, G., Hatch, S. F., Quispe Roncal, N. G., Gonzales Eslava, N., Huaylinos Bustamante, M. L., Castellanos, A., Hartinger, S. M., & Jäggi, L. (2025). Caregivers’ Perceptions and Practices Regarding Responsive Caregiving and Early Learning in Cajamarca, Andean Peru: A Qualitative Study. *Infant and Child Development*, *34*(4), e70036. https://doi.org/10.1002/icd.70036

Huaraz-Gutierrez, E. M., Ricse-Alcala, A. J., & Melendez-Jara, C. M. (2025). Percepción de la autoeficacia materna en madres primerizas adolescentes de la Amazonia peruana. *Revista de Psicología*, *43*(2), 913-940. https://doi.org/10.18800/psico.202502.010

Jara-Avellaneda, M. O., Huayta-Franco, Y. J., Arenas, E. R. S., & Flores, J. M. C. (2023). Motivation in virtual classrooms during COVID-19: Experiences of nursing students. *Salud, Ciencia y Tecnología*, *3*, 442-442. https://doi.org/10.56294/saludcyt2023442

Jaramillo, I. O., Gomez-Restrepo, C., Brusco, L. I., Diez-Canseco, F., Fung, C., Ariza-Salazar, K., Olivar, N., Toyama, M., Sureshkumar, D. S., Uribe-Restrepo, J. M., Carbonetti, F. L., Vilela-Estrada, A. L., & Priebe, S. (2025). Role of strategies used by young people for dealing with emotional distress: A qualitative study in deprived urban neighborhoods in Latin America. *Discover Mental Health*, *5*(1), 14. https://doi.org/10.1007/s44192-025-00143-3

Jesús, A. de, Álvarez Aguirre, A., & Jeuna Díaz Manchay, R. (2024). Uso de las plantas ancestrales durante el posparto mediato desde la cosmovisión nahua. *Medicina naturista*, *18*(2), 59-64.

Jimenez Sanchez, M. L., Gamarra, P., Brunner, J., Williams, H. A., LaNoire, M., Barrios, Y. V., Cruz, V. O., Rondon, M. B., Gelaye, B., & Levey, E. J. (2025). «I want to be a different kind of father»: A qualitative analysis of adolescent fatherhood in Perú. *Culture, Health & Sexuality*, *27*(6), 782-797. https://doi.org/10.1080/13691058.2024.2403773

Juárez-Chávez, E., Villalobos Ruiz, J. H., Konda, K. A., Urday-Fernández, D., & Cuba-Fuentes, M. S. (2025). Perceptions of primary care services among Afro-Peruvians in Lima, Peru. *Primary Health Care Research & Development*, *26*, e9. https://doi.org/10.1017/S1463423625000076

Julca-Chilcon, M. S., Diaz-Manchay, R. J., Guzman-Tello, S. M., Mesta-Delgado, R. del P., Julca-Chilcon, M. S., Diaz-Manchay, R. J., Guzman-Tello, S. M., & Mesta-Delgado, R. del P. (2022). Nurse-Patient Interaction in View of a Successful Surgical Process. *Revista Cubana de Enfermería*, *38*(2). http://scielo.sld.cu/scielo.php?script=sci_abstract&pid=S0864-03192022000200009&lng=en&nrm=iso&tlng=es

Kamichi Miyashiro, M. J. (2022). Intervenciones nutricionales de la empresa social Muna&Co. *Revista española de nutrición comunitaria = Spanish journal of community nutrition*, *28*(2), 12.

Lai, J. F., Clarke, J., de Wildt, G., Meza, G., Addo, M. A., Gardiner, E., & Khanna, D. (2022). Healthcare professionals’ perceptions of childhood obesity in Iquitos, Peru: A qualitative study. *BMC Health Services Research*, *22*(1), 175. https://doi.org/10.1186/s12913-022-07519-z

Lankowski, A., Tollefson, D., Sánchez, H., Cabello, R., Hidalgo, J., Mathison, M. N., Molina, Y., & Duerr, A. (2024). Acceptability of venue-based HIV testing and prevention interventions for men who have sex with transgender women and transgender women in Lima, Perú: A formative, qualitative study. *HIV Research & Clinical Practice*, *25*(1), 2331360.

Lazo-Gonzales, A. O., Sarmiento-Casavilca, T., Espinosa-Henao, O. E., Ruelas-González, M. G., & Alcalde-Rabanal, J. E. (2023). Looking at maternal health of Asháninka communities from the conceptual framework of the accessibility of care. *International Journal for Equity in Health*, *22*(1), 154. https://doi.org/10.1186/s12939-023-01943-1

Levey, E. J., Chang, A. R., Sanchez, M. L. J., Harrison, A. M., Rodriguez, A. E. M., Gelaye, B., & Rondon, M. B. (2023). Manifestations of Intergenerational Trauma During the Perinatal Period Among Adolescent Mothers in Lima, Peru: A Qualitative Analysis. *Journal of Child & Adolescent Trauma*, *16*(1), 21-30. https://doi.org/10.1007/s40653-022-00477-y

Levey, E. J., Rodriguez, A. E. M., Chang, A. R., Rondon, M. B., Sanchez, M. L. J., Harrison, A. M., Gelaye, B., & Becker, A. E. (2024). A qualitative analysis of adolescent motherhood within the broader family context in Peru. *Family Relations*, *73*(2), 1046-1066. https://doi.org/10.1111/fare.12904

Leyva-Moral, J. M., Moran-Paredes, G. I., Zegarra-Chapoñan, R., Chero-Pacheco, V. H., Gomez-Ibañez, R., & Aguayo-González, M. (2025). Experiences of nursing students and professors on teaching and learning about gender-based violence in Lima (Peru): A qualitative study. *BMC Nursing*, *24*(1), 825. https://doi.org/10.1186/s12912-025-03294-4

Linares-Olano, I. A., Cervera-Vallejos, M. F., Rodríguez-Cruz, L. D., Diaz-Manchay, R. J., Guerrero-Quiroz, E. S., Reluz-Barturen, F., Linares-Olano, I. A., Cervera-Vallejos, M. F., Rodríguez-Cruz, L. D., Diaz-Manchay, R. J., Guerrero-Quiroz, E. S., & Reluz-Barturen, F. (2023). VIRTUE ETHICS IN THE CARE OF COVID-19 PATIENTS IN PUBLIC HOSPITALS IN LIMA, PERU. *Ciencia y enfermería*, *29*. https://doi.org/10.29393/ce29-25evif60025

Lizarraga, K. J., Zizzi, C., Chunga, N., Quispe Moore, L. M., Risco, J., Valdovinos, B., Fernandez Macedo, V., Camargo Salazar, I., & Jozefowicz, R. (2025). Cross-cultural learning during an international exchange program in medical education: A qualitative study. *BMC Medical Education*, *25*(1), 1217. https://doi.org/10.1186/s12909-025-07763-x

Llanos-Zavalaga, L. F., Alarcón Tenorio, A., Llanos-Zavalaga, L. F., & Alarcón Tenorio, A. (2023). Creación e implementación del Sistema de Entrega de Medicamentos a Pacientes Crónicos SIENMECRO: Estudio cualitativo. *Anales de la Facultad de Medicina*, *84*(1), 70-75. https://doi.org/10.15381/anales.v84i1.23482

Lovera Anyosa, M. A., Díaz-Del Águila, F., Ninalaya Casallo, M., Huaranga Rivera, H. V., & Guerrero Meza, N. (2025). GOVERNANCE AND STRATEGIC PLANNING OF ICT PROJECTS FOR PUBLIC SAFETY IN LIMA. *TPM – Testing, Psychometrics, Methodology in Applied Psychology*, *32*(S2 (2025): Posted 09 June), 1281-1293.

Lustig Vijay, S., Harris, M., Friso, F., & Politi, M. (2024). Purging to Cleanse: A Qualitative Study of Ayahuasca Healing at a Drug Treatment Center in Peru. *Journal of Studies on Alcohol and Drugs*, *85*(5), 619-626. https://doi.org/10.15288/jsad.22-00429

Mamani, A. K. R., Manchay, R. J. D., & Vallejos, M. F. C. (2023). Desafíos éticos en el cuidado al paciente hospitalizado por COVID-19 desde la perspectiva de enfermeras peruanas. *Revista Cubana de Enfermería*, *39*. https://revenfermeria.sld.cu/index.php/enf/article/view/5854

Manrique, J. A. S., Ugaz, E. M. G., & Huayta-Franco, Y. J. (2023). Physiotherapists’ experiences of clinical practice: A Phenomenological Study. *Salud, Ciencia y Tecnología*, *3*, 528-528. https://doi.org/10.56294/saludcyt2023528

Maquera Maquera, Y. A., Bermejo Paredes, S., Maquera Maquera, Y., Chuquicallata Paricahua, S., Serruto Huanca, A., Gutiérrez Díaz, C. A., & Olivera Condori, E. (2025). Enseñanza de la Educación Física desde la perspectiva territorial y encuentro con el estudiante: Percepciones de masterandos en ciencias del deporte. *Retos: nuevas tendencias en educación física, deporte y recreación*, *62*, 1107-1116.

Maquera Maquera, Y. A., Bermejo Paredes, S., Olivera Condori, E., Cahuana Tapia, R. D., Pino Vanegas, Y. M., Yupanqui Pino, E. H., Chuquicallata Paricahua, S., & Vilca Apaza, H. M. (2024). Intrincada construcción de identidad profesional y relaciones de poder: Percepciones en docentes universitarios de Educación Física. *Retos: nuevas tendencias en educación física, deporte y recreación*, *55*, 946-956.

Maquera Maquera, Y. A., Olivera Condori, E., Bermejo Gonzáles, L. Y., & Bermejo Paredes, S. (2024). Tecnologías inmersivas y atención a la diversidad territorial en Educación Física. *Retos: nuevas tendencias en educación física, deporte y recreación*, *54*, 141-150.

Maquera-Maquera, Y. A., Bermejo-paredes, S., Condori, E. O., & Maquera, Y. M. (2025). Competencias y rol docente de educación física en nuevos contextos formativos. *Retos*, *66*, 599-608. https://doi.org/10.47197/retos.v66.112200

Marcelo, E. M. G., Huarcaya, S. S. L., & Ferreira, M. de A. (2024). REPRESENTACIÓN SOCIAL DE LA SOLEDAD EN PERSONAS SEPTUAGENARIAS Y DE MAYOR EDAD. *Texto & Contexto - Enfermagem*, *33*, e20230143. https://doi.org/10.1590/1980-265X-TCE-2023-0143es

Mayo-Puchoc, N., Bejarano-Carranza, J., Paredes-Angeles, R., Vilela-Estrada, A. L., García-Serna, J., Cusihuaman-Lope, N., Villarreal-Zegarra, D., Cavero, V., & Ardila-Gómez, S. (2023). Paper promises: Peruvian frontline health workers’ perspectives on mental health policies during COVID-19. *Health Policy and Planning*, *38*(Suppl 2), ii3-ii13. https://doi.org/10.1093/heapol/czad055

Meneses-La-Riva, M. E., Fernández-Bedoya, V. H., Suyo-Vega, J. A., Ocupa-Cabrera, H. G., & Paredes-Díaz, S. E. (2025). Humanized Care in Nursing Practice: A Phenomenological Study of Professional Experiences in a Public Hospital. *International Journal of Environmental Research and Public Health*, *22*(8), 1223. https://doi.org/10.3390/ijerph22081223

Merino Lozano, A. L., Valderrama Rios, O. G., Mamani Macedo, M. L., Vargas Pinedo, M. E., Ramírez García, E., Cuba Sancho, J. M., Merino Lozano, A. L., Valderrama Rios, O. G., Mamani Macedo, M. L., Vargas Pinedo, M. E., Ramírez García, E., & Cuba Sancho, J. M. (2024). Living Experiences of Nurses with COVID-19 and Isolated in the Family Environment. *Revista Cubana de Enfermería*, *40*. http://scielo.sld.cu/scielo.php?script=sci_abstract&pid=S0864-03192024000100016&lng=en&nrm=iso&tlng=es

Mogollón Torres, F. de M., Díaz Vásquez, M. A., Murillo Ruiz, I. F., Villarreal Ortiz, Y. B., Díaz Manchay, R., & Vega Ramírez, A. S. (2024). Cuidados en el hogar al niño hospitalizado con infecciones respiratorias agudas desde un enfoque cultural. *Cultura de los cuidados: Revista de Enfermería y Humanidades*, *69*, 299-313.

Monteagudo, N. C., Camacho Rodríguez, D. E., Gonzales Carhuajulca, D. B., Leyva Moral, J. M., & Martínez, O. N. (2025). Defining nursing entrepreneurship from the point of view of future professionals: A qualitative study. *Nurse Education Today*, *144*, 106421. https://doi.org/10.1016/j.nedt.2024.106421

Morse, R. M., Brown, J., Noble, H. E., Ríos López, E. J., Kohler-Smith, A., Soto, S., Del Cuadro, D. L., Gonzales Díaz, K., Figueredo Escudero, M., Vásquez Del Aguila, G., Carrillo Jara, L. E., Silva Delgado, H. F., Palacios, V. A., Santos-Ortiz, C., Gravitt, P. E., Paz-Soldan, V. A., & Proyecto Precancer Study Group. (2022). Women’s perspectives on the acceptability and feasibility of an HPV screen-and-treat approach to cervical cancer prevention in Iquitos, Peru: A qualitative study. *BMC Women’s Health*, *22*(1), 414. https://doi.org/10.1186/s12905-022-01943-3

Morse, R. M., Jurczuk, M., Brown, J., Jara, L. E. C., Meza, G., López, E. J. R., Tracy, J. K., Gravitt, P. E., Paz-Soldan, V. A., & Proyecto Precancer Study Group. (2023). «Day or night, no matter what, I will go»: Women’s perspectives on challenges with follow-up care after cervical cancer screening in Iquitos, Peru: A qualitative study. *BMC Women’s Health*, *23*(1), 293. https://doi.org/10.1186/s12905-023-02414-z

Moya-Salazar, J., Cañari, B., Zuñiga, N., Jaime-Quispe, A., & Contreras-Pulache, H. (2023). The End of Life Accompanied by COVID-19: A Qualitative Study on Changes in Behavior and Stigmatization of the Grieving Families in Peru (Part II). *Omega*, 302228231212655. https://doi.org/10.1177/00302228231212655

Moya-Salazar, J., Zuñiga, N., Cañari, B., Jaime-Quispe, A., Chicoma-Flores, K., & Contreras-Pulache, H. (2022). The End of Life Accompanied by COVID-19: A Qualitative Study on Grief During the First OutBreak In Peru (Part I). *Omega*, 00302228221134424. https://doi.org/10.1177/00302228221134424

Navarro-Ordinola, G., Mogollón-Torres, F. de M., Vega-Ramírez, A. S., Díaz-Manchay, R. J., Navarro-Ordinola, G., Mogollón-Torres, F. de M., Vega-Ramírez, A. S., & Díaz-Manchay, R. J. (2024). Experiences of the Health Team in the Application of Skin-To-Skin Contact. *Revista Cubana de Pediatría*, *96*. http://scielo.sld.cu/scielo.php?script=sci_abstract&pid=S0034-75312024000100002&lng=en&nrm=iso&tlng=es

Naz-McLean, S., Clark, J., Huerta, L., Mayer, K. H., Lama, J. R., Reisner, S., & Perez-Brumer, A. (2024). Social, economic, and physical side effects impact PrEP uptake and persistence among transgender women in Peru. *BMC Public Health*, *24*(1), 1985. https://doi.org/10.1186/s12889-024-19474-x

Naz-McLean, S., Clark, J. L., Reisner, S. L., Prenner, J. C., Weintraub, B., Huerta, L., Salazar, X., Lama, J. R., Mayer, K. H., & Perez-Brumer, A. (2022). Decision-Making at the Intersection of Risk and Pleasure: A Qualitative Inquiry with Trans Women Engaged in Sex Work in Lima, Peru. *AIDS and Behavior*, *26*(3), 843-852. https://doi.org/10.1007/s10461-021-03445-z

Nunez, A., Curtis, M., Wong, M., Kosyluk, K. A., Galea, J. T., Franke, M. F., & Errea, R. A. (2025). Change targets, messaging, and content delivery for a community-engaged social media campaign addressing HIV-related stigma in Peru: A qualitative study. *medRxiv: The Preprint Server for Health Sciences*, 2025.02.12.25321801. https://doi.org/10.1101/2025.02.12.25321801

Ochoa-Panaifo, M., Ramirez-Heros, A., & Malvaceda-Espinoza, E. (2024). Experiencias del pre, post y durante tratamiento del EPR online en personas con TOC, en contexto de pandemia. *Revista Latinoamericana de Psicopatologia Fundamental*, *27*, e230855. https://doi.org/10.1590/1415-4714.e230855

Oliden Chavez, E. del P., Díaz Vásquez, M. A., Díaz Manchay, R. J., Oliden Chavez, E. del P., Díaz Vásquez, M. A., & Díaz Manchay, R. J. (2023). Percepción del profesional de enfermería sobre los efectos del cambio climático en la salud infantil. *Revista Cubana de Enfermería*, *39*. http://scielo.sld.cu/scielo.php?script=sci_abstract&pid=S0864-03192023000100051&lng=es&nrm=iso&tlng=es

Oliva Rapoport, V. E., Altamirano, E., Senador, L., Wong, M., Beckhorn, C. B., Coit, J., Roche, S. D., Lecca, L., Galea, J. T., & Chiang, S. S. (2022). Impact of prolonged isolation on adolescents with drug-susceptible tuberculosis in Lima, Peru: A qualitative study. *BMJ Open*, *12*(9), e063287. https://doi.org/10.1136/bmjopen-2022-063287

Ordoñez Espinoza, L. A., Santos Falcón, G. C., Zeladita Huaman, J. A., Zegarra Chapoñán, R., Ordoñez Espinoza, L. A., Santos Falcón, G. C., Zeladita Huaman, J. A., & Zegarra Chapoñán, R. (2022). Resignification of Life by Young People Survivors of Hematologic Cancer. *Revista Cubana de Enfermería*, *38*(3). http://scielo.sld.cu/scielo.php?script=sci_abstract&pid=S0864-03192022000300012&lng=en&nrm=iso&tlng=es

Orozco-Poore, C., Perez-Brumer, A., Huerta, L., Salazar, X., Nunez, A., Nakamura, A., Aguayo-Romero, R., Silva-Santisteban, A., & Reisner, S. L. (2024). The «Cycle» of HIV: Limits of Personal Responsibility in HIV Vulnerability Among Transgender Adolescents and Young Women in Lima, Peru. *AIDS and Behavior*, *28*(11), 3893-3907. https://doi.org/10.1007/s10461-024-04462-4

Paredes Ajalla, A. M., Shishido Sánchez, S., Paredes Ajalla, A. M., & Shishido Sánchez, S. (2022). Percepción y disposición al tacto rectal en la prevención de cáncer de próstata. *Anales de la Facultad de Medicina*, *83*(1), 49-53. https://doi.org/10.15381/anales.v83i1.20779

Paredes-Angeles, R., Cavero, V., Vilela-Estrada, A. L., Cusihuaman-Lope, N., Villarreal-Zegarra, D., & Diez-Canseco, F. (2024). Telehealth in community mental health centers during the COVID-19 pandemic in Peru: A qualitative study with key stakeholders. *SSM. Mental Health*, *5*, 100287. https://doi.org/10.1016/j.ssmmh.2023.100287

Pease Dreibelbis, M. A., Urbano Flores, E., & De la Puente Ronceros, R. A. (2024). «I will attend to college to give my family a better life»: Indebtedness with the family and the challenges of building occupational plans for Peruvian adolescents. *Journal of Research on Adolescence: The Official Journal of the Society for Research on Adolescence*, *34*(2), 477-489. https://doi.org/10.1111/jora.12961

Pedersen, G. A., Elnasseh, A., Bhattacharya, B., Moran, L., Neupane, V., Galea, J. T., Contreras, C., Pfeffer, K. A., Brown, A. D., Sangraula, M., Luitel, N. P., & Kohrt, B. A. (2023). Practitioners’ perspectives on preparing for and delivering remote psychological support in Nepal, Perú and the United States during COVID-19. *Psychology and Psychotherapy*, *96*(4), 849-867. https://doi.org/10.1111/papt.12476

Pérez, F. E. L., Montero, J. M. C., Navarro, E. R., Meléndez, L. V., & Salinas, A. P. M. H. de. (2024). Arrepentimiento y esperanza de unión familiar en madres de infantes recluidas en Perú. *Revista Cubana de Medicina Militar*, *53*(2), e02456116-e02456116.

Perez Mogrovejo, F., Villaseca Cruz, J., Cubillas Espinoza, P., Rivera Cruzatt, F., Malvaceda-Espinoza, E., Perez Mogrovejo, F., Villaseca Cruz, J., Cubillas Espinoza, P., Rivera Cruzatt, F., & Malvaceda-Espinoza, E. (2024). Percepción de riesgo de contagio del COVID-19 en personal de salud. *Revista de Psicología (PUCP)*, *42*(1), 240-266. https://doi.org/10.18800/psico.202401.009

Perez-Lluncor, M. F., Cervera-Vallejos, M. F., Diaz-Manchay, R. J., Perez-Lluncor, M. F., Cervera-Vallejos, M. F., & Diaz-Manchay, R. J. (2022). La ética de los cuidados en la trayectoria universitaria de enfermería. *Revista Cubana de Enfermería*, *38*(4). http://scielo.sld.cu/scielo.php?script=sci_abstract&pid=S0864-03192022000400006&lng=es&nrm=iso&tlng=.

Polo Campos, F. H., Tejada Muñoz, S., Palomino Salazar, M. Y., Vega Ramírez, A. S., Mogollón Torres, F. de M., & Díaz Manchay, R. (2022). Prácticas culturales de higiene bucal y lavado de manos en escolares de una zona urbano marginal. *Cultura de los cuidados: Revista de Enfermería y Humanidades*, *64*, 161-175.

Priego, M. K. M. D. (2023). Exploración cualitativa de la experiencia de flow en bailarinas de ballet que lo practican como hobby. *Revista de Psicología Aplicada al Deporte y al Ejercicio Físico*, *8*(1), 2.

Puicón-Mejía, M. J., Díaz-Vasquez, M. A., & Díaz-Manchay, R. J. (2024). Percepciones y actitudes sobre cambio climático en estudiantes universitarios de Enfermería en Perú. *Index de Enfermería*, e14699-e14699. https://doi.org/10.58807/indexenferm20246865

Quispe-Mamani, E., Pineda-Palomino, K. P., Poma-Callo, Y., Quispe-Huaranca, I. L., & Inquilla-Mamani, J. (2022). Organizational Management of Primary Care in the Regional Health System in Puno, Peru. *Revista de Salud Pública*, *24*(4), 1. https://doi.org/10.15446/rsap.v24n4.101006

Ramón, R. N. R., & Giove, M. V. V. (2023). Sistema de Creencias Familiares e Infidelidad en Parejas Convivientes de Tarapoto. *PsiqueMag*, *12*(1), 82-92. https://doi.org/10.18050/psiquemag.v12i1.2490

Ramos Castro, R. E. (2022). Cambios en el estilo de vida de mujeres mastectomizadas. *Revista Cubana de Enfermería*, *38*(4), 5.

Reisner, S. L., Aguayo-Romero, R. A., Perez-Brumer, A., Salazar, X., Nunez-Curto, A., Orozco-Poore, C., & Silva-Santisteban, A. (2023). A life course health development model of HIV vulnerabilities and resiliencies in young transgender women in Peru. *Global Health Research and Policy*, *8*(1), 32. https://doi.org/10.1186/s41256-023-00317-y

Rivas-Chapoñan, J. D., Cervera-Vallejos, M. F., Diaz-Manchay, R. J., Rivas-Chapoñan, J. D., Cervera-Vallejos, M. F., & Diaz-Manchay, R. J. (2022). Intervención terapéutica trascendental del profesional de enfermería al familiar acompañante en etapa de duelo. *Revista Cubana de Enfermería*, *38*(2). http://scielo.sld.cu/scielo.php?script=sci_abstract&pid=S0864-03192022000200003&lng=es&nrm=iso&tlng=es

Rivera-Cruzatt, F. D., Cubillas-Espinoza, P. P., & Malvaceda-Espinoza, E. L. (2022). Psychological coping in female breast cancer patients from a Metropolitan Lima hospital. *Revista Peruana De Medicina Experimental Y Salud Publica*, *39*(4), 400-407. https://doi.org/10.17843/rpmesp.2022.394.12322

Rivera-Miranda Giral, C. M., Díaz-Manchay, R. J., & León-Jiménez, F. E. (2024). What it means to live with fibromyalgia for Peruvian women: A phenomenological study. *Revista Colombiana de Reumatología*, *31*(3), 339-348. https://doi.org/10.1016/j.rcreu.2023.07.003

Rozas, L., & Busse, P. (2022). The foodscapes of children and adolescents attending schools in Lima, Peru. *Appetite*, *168*, 105738. https://doi.org/10.1016/j.appet.2021.105738

Rubio, M. C. D., & Asenjo-Alarcón, J. A. (2023). Mujeres andinas: Actitudes en el uso de plantas para el tratamiento de eventos ginecológicos. *Revista Cuidarte*, *14*(2). https://doi.org/10.15649/cuidarte.2724

Safary, E., Beran, D., Vetter, B., Lepeska, M., Abdraimova, A., Dunganova, A., Besançon, S., Lazo-Porras, M., Mazanett, J. P., Pérez-León, S., Maixenchs, M., Nchimbi, H., Ramaiya, K., Munishi, C., & Martínez-Pérez, G. Z. (2024). *User requirements for non-invasive and minimally invasive glucose self-monitoring devices in low-income and middle-income countries: A qualitative study in Kyrgyzstan, Mali, Peru and Tanzania*. https://doi.org/10.1136/bmjopen-2023-076685

Salinas-Gutierrez, M. P., Díaz-Manchay, R. J., Rodríguez-Cruz, L. D., Cervera-Vallejos, M. F., Constantino-Facundo, F., & Tejada-Muñoz, S. (2024). Experiencias de las enfermeras en primera línea contagiadas con COVID-19 que recibieron atención domiciliaria. *Cultura de los Cuidados*, *68*, 297-312. https://doi.org/10.14198/cuid.22906

Sánchez Chanamé, S. L. del C., Díaz Manchay, R., Cervera Vallejos, M. F., Rodriguez Cruz, L. D., Huyhua Gutierrez, S. C., & Tejada Muñoz, S. (2025). Experiencias de estudiantes peruanos de Enfermería sobre el retorno a las clases presenciales. *Revista Cubana de Enfermería*, *41*(0), 5.

Sedano, C. A. C., Hinojosa Núñez, M. L., Veli Rojas, D. D., & Rodríguez, M. N. C. (2022). Percepciones sanitarias y educativas condicionadas por la pandemia COVID-19: Intercambio internacional de experiencias docentes, 2021. *Boletin de Malariologia y Salud Ambiental*, *62*(3), 461. https://doi.org/10.52808/bmsa.7e6.623.012

Sologuren-García, G., Linares, C. L., Flores, J. R., Mendoza-Mamani, K., Pilco, R. M., Escobar-Bermejo, G., Sotelo-Gonzales, S., & Palza-Portugal, G. (2023). Breaking the taboo: Qualitative analysis of the sexuality in people with acquired motor disability. *BMC Psychology*, *11*(1), 380. https://doi.org/10.1186/s40359-023-01423-9

Subileta-Yangali, R. A., Rojas-Vargas, S. G., & Malvaceda-Espinoza, E. (2025). Experiences of emotional dependency in lesbian couples. *Estudos de Psicologia (Campinas)*, *42*, e220021. https://doi.org/10.1590/1982-0275202542e220021

Tamayo, G. K. S., Angulo, F. M. del R. H., & Rosa, R. M. A. S. C. (2024). CUIDADO ESPIRITUAL DESDE CARITAS VERITAS DE WATSON EN ENFERMERAS DE UN HOSPITAL DISTRITAL EN PERÚ. *Ciencia y Enfermería*, *30*. https://doi.org/10.29393/CE30-19CEGR30019

Tapullima-Mori, C., Apolin, D. E. M., & Bautista, S. D. B. (2024). Estrategias didácticas en la asesoría de tesis para desarrollar competencias investigativas: Perspectivas de docentes y estudiantes. *Revista Digital de Investigación en Docencia Universitaria*, *18*(2), e1884-e1884. https://doi.org/10.19083/ridu.2024.1884

Tarazona-Meza, C., Bartolini, R. M., Romero, K., Pradeilles, R., Goya, C., Rousham, E. K., Griffiths, P. L., & Creed-Kanashiro, H. M. (2025). Facilitators and barriers to anemia prevention in the urban government childcare program for infants and young children in Peru. *Global Health Action*, *18*(1), 2475580. https://doi.org/10.1080/16549716.2025.2475580

Tejada Muñoz, S., Huyhua Gutierrez, S. C., Díaz Manchay, R. J., Herrera Garcia, Y. I., Sandoval-Bances, J. C., Chenet, S. M., Tejada Muñoz, S., Huyhua Gutierrez, S. C., Díaz Manchay, R. J., Herrera Garcia, Y. I., Sandoval-Bances, J. C., & Chenet, S. M. (2024). Reflexiones sobre el cuidado ambiental desde la perspectiva de estudiantes universitarios de Enfermería. *Revista Cubana de Enfermería*, *40*. http://scielo.sld.cu/scielo.php?script=sci_abstract&pid=S0864-03192024000100013&lng=es&nrm=iso&tlng=es

Tejada Muñoz, S., Jeuna Díaz Manchay, R., Huyhua Gutierrez, S. C., Hinojosa Salazar, C. A., & Vega Ramírez, A. S. (2022). Beneficios de la musicoterapia desde la mirada de los estudiantes universitarios de enfermería. *Medicina naturista*, *16*(1), 21-26.

Temelkovska, T., Moriarty, K., Huerta, L., Perez-Brumer, A., Segura, E., Passaro, R. C., Lake, J. E., Clark, J., & Blair, C. (2023). Social Networks Play a Complex Role in HIV Prevention Knowledge, Attitudes, Practices, and the Uptake of PrEP Through Transgender Women Communities Centered Around Three “Casas Trans” in Lima, Peru: A Qualitative Study. *Journal of the International Association of Providers of AIDS Care (JIAPAC)*, *22*, 23259582231196705. https://doi.org/10.1177/23259582231196705

Tenorio-Mucha, J., Portocarrero, J., Busta-Flores, P., Pesantes, M. A., & Lazo-Porras, M. (2022). Percepciones de aceptabilidad y reticencia a las vacunas contra la COVID-19 en el Perú. *Revista Peruana de Medicina Experimental y Salud Pública*, 274-280. https://doi.org/10.17843/rpmesp.2022.393.11337

Torres-Fernandez, S. L., Vega Ramírez, A. S., Mogollón Torres, F. de M., Díaz Manchay, R. J., & Tejada Muñoz, S. (2024). *Interculturalidad: Un reto en la formación de enfermeros*. http://hdl.handle.net/10045/147954

Torres-Slimming, P. A., Carcamo, C., Martínez-Pérez, G. Z., Mallma, P., Pflucker, C., & Shilton, S. (2023). Rapid SARS-CoV-2 Antigen Detection Self-Tests to Increase COVID-19 Case Detection in Peru: Qualitative Study. *JMIR Formative Research*, *7*(1), e43183. https://doi.org/10.2196/43183

Toyama, M., Cavero, V., Araya, R., Menezes, P. R., Mohr, D. C., Miranda, J. J., & Diez-Canseco, F. (2022). Participants’ and Nurses’ Experiences With a Digital Intervention for Patients With Depressive Symptoms and Comorbid Hypertension or Diabetes in Peru: Qualitative Post–Randomized Controlled Trial Study. *JMIR Human Factors*, *9*(3), e35486. https://doi.org/10.2196/35486

Toyama, M., Godoy-Casasbuenas, N., Olivar, N., Brusco, L. I., Carbonetti, F., Diez-Canseco, F., Gómez-Restrepo, C., Heritage, P., Hidalgo-Padilla, L., Uribe, M., Steffen, M., Fung, C., & Priebe, S. (2022). Identifying resources used by young people to overcome mental distress in three Latin American cities: A qualitative study. *BMJ Open*, *12*(8), e060340. https://doi.org/10.1136/bmjopen-2021-060340

Toyama, M., Vilela-Estrada, A. L., Ariza-Salazar, K., Osorio Jaramillo, I., Ramirez-Meneses, D., Flores, S., Carbonel, A., Olivar, N., Carbonetti, F. L., Fung, C., Stanislaus Sureshkumar, D., Brusco, L. I., Gómez-Restrepo, C., Diez-Canseco, F., & Priebe, S. (2025). Resources used by young people to overcome mental distress in deprived settings in Latin America: A qualitative study. *BMC Psychology*, *13*(1), 727. https://doi.org/10.1186/s40359-025-02830-w

Ubillús, J. L. V., Enríquez, B. K. L., Lozano, R. E. G., & Jiménez, F. E. L. (2023). Mitos y creencias sobre la insulinoterapia en pacientes con diabetes mellitus y sus familiares de un hospital del norte del Perú, 2020. *Revista Peruana de Medicina Experimental y Salud Pública*, 42-50. https://doi.org/10.17843/rpmesp.2023.401.12210

Ulco-Bravo, J., Cervera-Vallejos, M. F., Díaz-Manchay, R., Saavedra-Covarrubia, M., & Constantino-Facundo, F. (2022). El hogar recinto para sostener la vida sujeta a diálisis peritoneal: Experiencia de cuidadores familiares. *Enfermería Nefrológica*, *25*(2), 125-131. https://doi.org/10.37551/52254-28842022013

Uzátegui-Gamarra, R., & Malvaceda-Espinoza, E. (2023). Adaptación Psicosocial en Padres de Hijos Diagnosticados con Trastorno del Espectro Autista. *Psicologia: Teoria e Pesquisa*, *39*, e39230. https://doi.org/10.1590/0102.3772e39230.es

Valenzuela Antezana, P., Inglish Bravo, A. A., Malvaceda-Espinoza, E., Mamani-Benito, O., & Carranza Esteban, R. F. (2024). Percepción del duelo de adultos que han perdido a un familiar durante la situación de pandemia en Lima Metropolitana. *Revista Colombiana de Psiquiatría*, *53*(4), 488-495. https://doi.org/10.1016/j.rcp.2022.09.003

VanDerWal, J. A., Dentice, A., Zavala-Wong, G., Chavarria, M., Morales, C. E., Agrawal, A., Blaser, M., Pittman, S., LaGrone, L. N., Arredondo-Manrique, G., Betalleluz Pallardel, J. R., Aragon-Graneros, G., Rodríguez-Castro, M., Borda-Luque, G., Castro-Dolorier, A., Allagual, A., Huamán-Egoávil, E., & Iverson, K. R. (2025). A qualitative needs assessment of Lima’s prehospital emergency trauma system: Identifying challenges and opportunities for improvement. *Surgery*, *185*, 109519. https://doi.org/10.1016/j.surg.2025.109519

Velásquez, P. del M. S., Manchay, R. J. D., Ramírez, A. S. V., Cruz, L. D. R., Gutierrez, S. C. H., & Muñoz, S. T. (26). Participación de la familia en la estimulación psicomotriz de lactantes mayores en una zona urbano-marginal. *Cultura de los Cuidados*, *62*, 206-224. https://doi.org/10.14198/cuid.2022.62.15

Velázquez, T., & Bravo, P. R. (2024). Migration Processes in Indigenous Women Post Internal Armed Conflict in Peru. *Revista Interamericana de Psicología/Interamerican Journal of Psychology*, *58*(2), e1652. https://doi.org/10.30849/ripijp.v58i2.1652

Williams, K. N., Tenorio-Mucha, J., Campos-Blanco, K., Underhill, L. J., Valdés-Velásquez, A., Herbozo, A. F., Beres, L. K., Fuentes, L. de las, Cordova-Ascona, L., Vela-Clavo, Z., Cuentas-Canal, G. M., Mendoza-Velasquez, J. C., Paredes-Barriga, S. M., Rosa, R. H. L., Williams, M., Geng, E. H., Checkley, W., Gittelsohn, J., Davila-Roman, V. G., & Hartinger-Peña, S. M. (2024). Health system barriers to hypertension care in Peru: Rapid assessment to inform organizational-level change. *PLOS Global Public Health*, *4*(8), e0002404. https://doi.org/10.1371/journal.pgph.0002404

Wilson, S. N., Noble, H., Ordoñez, W. J. N., Wong, G. Z., Rodríguez, M. J., Checa, D. O., Warne, M., Senturia, K., & LaGrone, L. N. (2023). Implementing point-of-care medical information systems into trauma and general surgeon practice in a middle-income country: A qualitative study utilizing the Consolidated Framework for Implementation Research. *Implementation Science Communications*, *4*(1), 38. https://doi.org/10.1186/s43058-023-00397-4

Woodson, L. L., Garcia Saldivar, A., Brown, H. E., Magrath, P. A., Farland, L. V., Blas, M. M., & Madhivanan, P. (2024). ‘You have a lot of mirrors’: Structural and socioecological factors impacting adolescent pregnancy and reproductive health in the Amazon basin, Peru, a qualitative study. *Culture, Health & Sexuality*, *26*(10), 1268-1284. https://doi.org/10.1080/13691058.2024.2308666

Woodson, L. L., Saldivar, A. G., Brown, H. E., Magrath, P. A., Mayolo, N. A. de, Pettygrove, S., Farland, L. V., Madhivanan, P., & Blas, M. M. (2024). The downstream effects of COVID-19 on adolescent girls in the Peruvian Amazon: Qualitative findings on how the pandemic affected education and reproductive health. *BMJ Global Health*, *9*(4). https://doi.org/10.1136/bmjgh-2023-012391

Yslado-Méndez, R., Escobar-Agreda, S., Vilela-Estrada, A. L., Villarreal-Zegarra, D., Sánchez-Broncano, J. D., Olivares Cordova, J. H., Trejo Flores, W. M., Alvarez-Yslado, C., & Rojas-Mezarina, L. (2025). Perceptions, beliefs, and attitudes toward mental health and the implementation of digital mental health interventions in a university community in an Andean region: A qualitative study. *SSM - Mental Health*, *8*, 100541. https://doi.org/10.1016/j.ssmmh.2025.100541

Zafra-Tanaka, J. H., Almeida, G., Andrade Montalvo, J. E., Anza-Ramirez, C., Jauregui, J., Perez-Leon, S., Lazo-Porras, M., Mayo-Puchoc, N., Taype-Rondán, A., Miranda, J. J., & Beran, D. (2024). Involving different stakeholders in prioritising outcomes to assess healthcare systems response for type 1 diabetes management: Using co-creation approaches in Peru. *The International Journal of Health Planning and Management*, *39*(6), 1675-1695. https://doi.org/10.1002/hpm.3821

Zafra-Tanaka, J. H., Portocarrero, J., Abanto, C., Zunt, J. R., & Miranda, J. J. (2022). Managing Post-Stroke Care During the COVID-19 Pandemic at a Tertiary Care Level Hospital in Peru. *Journal of Stroke and Cerebrovascular Diseases*, *31*(4). https://doi.org/10.1016/j.jstrokecerebrovasdis.2021.106275

Zuleta, M., Perez-Leon, S., Mialon, M., & Delgado-Zegarra, J. (2023). Political and socioeconomic factors that shaped health taxes implementation in Peru. *BMJ Global Health*, *8*(Suppl 8). https://doi.org/10.1136/bmjgh-2023-012024
