# Supplementary material for: Reporting quality of qualitative health studies published by Peruvian authors: A scoping review
Source: PLoS One. 2026 Jun 23;21(6):e0351494. doi: 10.1371/journal.pone.0351494 (PMC13289893; doi:10.1371/journal.pone.0351494)
Supplement: S3 Table — (DOCX) [file pone.0351494.s003.docx]

**S3 Table.** Table of excluded studies (n=55)

| **N°** | **Study title** | **Year** | **Reason for exclusion** |
| --- | --- | --- | --- |
| 1 | Outcomes and Experiences of Patients and Their Caregivers After Severe Stroke Requiring Tube Feeding in Peru | 2024 | Mixed-methods study |
| 2 | Impact of hospital characteristics on implementation of a Pediatric Early Warning System in resource-limited cancer hospitals | 2023 | Mixed-methods study |
| 3 | Dental Superheroine: A Culturally Adapted Storytelling Strategy for Oral Health Education in Rural Peru | 2025 | Mixed-methods study |
| 4 | Analysis of binge-watching on streaming services by Peruvian young adults | 2022 | Not published in a peer-reviewed journal |
| 5 | Perceptions of Problems with Household Insects: Qualitative and Quantitative Findings from Peri-Urban Communities in Arequipa, Peru | 2023 | Mixed-methods study |
| 6 | Vulnerabilidad de la infraestructura sanitaria en relación a la COVID-19 en el distrito de Castilla-Piura 2021–2022 | 2023 | Mixed-methods study |
| 7 | Latin American patients’ perceptions on osteoporosis diagnosis and treatment: The IOF Patient Advisory Group in Latin America | 2025 | Mixed-methods study |
| 8 | Telehealth Actions to Address COVID-19 in Latin American Countries | 2023 | Peruvian population size not specified |
| 9 | Assessment of a Pilot Program for Remote Support on Mental Health for Young Physicians in Rural Settings in Peru: Mixed Methods Study | 2024 | Mixed-methods study |
| 10 | Association between time of residence and self-perception of distress, interpersonal relationships, and social role in Venezuelan immigrants in Lima, Peru 2018–19: mixed-methods study | 2022 | Mixed-methods study |
| 11 | Cáncer cervicouterino en el contexto de la pandemia de COVID-19 en comunidades rurales de Ayacucho | 2024 | Mixed-methods study |
| 12 | Women’s mental health in the doctoral context: Protective function of the psychological capital and academic motivation | 2025 | Mixed-methods study |
| 13 | Understanding linkage to biopsy and treatment for breast cancer after a high-risk telemammography result in Peru: a mixed-methods study | 2022 | Mixed-methods study |
| 14 | Barreras y facilitadores en la implementación de un sistema de tele-triaje para la identificación remota de casos sospechosos de COVID-19 por estudiantes voluntarios | 2024 | Mixed-methods study |
| 15 | Patient Engagement With and Perceptions of the COVIDA Project, a Volunteer-Led Telemonitoring and Teleorientation Service for COVID-19 Community Management: Mixed Methods Study | 2024 | Mixed-methods study |
| 16 | Influencias socioculturales que enfrenta la mujer futbolista en el departamento de Puno | 2022 | Not health-related topic |
| 17 | El uso del consentimiento informado en la práctica odontológica en el sur del Perú | 2024 | Mixed-methods study |
| 18 | A design science approach to mixed-methods evaluation in serious game research | 2025 | Mixed-methods study |
| 19 | Task Sharing and Remote Delivery of Brief Interpersonal Counseling for Venezuelan Migrants and Refugees Living in Peru during the COVID-19 Pandemic: A Mixed-Methods Pilot Study | 2024 | Mixed-methods study |
| 20 | Pedagogical accompaniment in Peruvian Amazon: conceptions, process and problems | 2024 | Not health-related topic |
| 21 | A mixed methods evaluation of a World Health Organization competency-based training package for foundational helping skills among pre-service and in-service health workers in Nepal, Peru and Uganda | 2023 | Mixed-methods study |
| 22 | My Brother the “Other”: Use of Satire and Boundary-Making by Venezuelan Migrants in Peru | 2022 | Mixed-methods study |
| 23 | Documenting adaptations to an evidence-based intervention in 58 resource-variable pediatric oncology hospitals across implementation phases | 2024 | Mixed-methods study |
| 24 | Establishing the health and wellbeing needs of mining host community in Brazil, Chile and Peru: a mixed-method approach to identify priority areas for action to help communities meet their SDG3 targets | 2023 | Mixed-methods study |
| 25 | Analysis of Facebook publications and comments about ivermectin during the COVID-19 pandemic in Peru | 2022 | Mixed-methods study |
| 26 | Ayahuasca Treatment Outcome Project (ATOP): One-Year Results from Takiwasi Center and Implications for Psychedelic Science | 2023 | Mixed-methods study |
| 27 | Exploring treatment decision-making at diagnosis for children with advanced cancer in low- and middle-income countries | 2024 | Mixed-methods study |
| 28 | Actions against the double burden of malnutrition in Peru: a community-informed system dynamics model | 2025 | Mixed-methods study |
| 29 | Evaluation of Women's Empowerment in a Community-Based Human Papillomavirus Self-Sampling Social Entrepreneurship Program (Hope Project) in Peru: A Mixed-Method Study | 2022 | Mixed-methods study |
| 30 | When street sexual harassment becomes invisible: Some factors that promote its tolerance | 2022 | Mixed-methods study |
| 31 | Development and Pilot Testing of PrOFILE-ST: A Pediatric Surgical Oncology Capacity and Quality Assessment Tool for Resource-Limited Settings | 2025 | Mixed-methods study |
| 32 | Boosting self-efficacy and improving practices for smoking prevention and cessation among South American cancer care providers with a web-based algorithm | 2024 | Mixed-methods study |
| 33 | Both/And: Mixed methods analysis of network composition, communication patterns, and socio-economic support within social networks of transgender women involved in sex work in Lima, Peru | 2023 | Mixed-methods study |
| 34 | Prevalence and exploration of HIV pre-exposure prophylaxis awareness in men who have sex with men aged 18–29 years in Lima, Peru, during 2021: A mixed methods study | 2025 | Mixed-methods study |
| 35 | Identifying access barriers faced by rural and dispersed communities to better address their needs: implications and lessons learned for rural proofing for health in the Americas and beyond | 2023 | Mixed-methods study |
| 36 | Factors Associated with Mental Health Outcomes in Hospital Workers during the COVID-19 Pandemic: A Mixed-Methods Study | 2022 | Mixed-methods study |
| 37 | Eficacia de un programa de terapia nutricional domiciliaria ambulatoria | 2025 | Mixed-methods study |
| 38 | Digital tools to improve parenting behaviour in low-income settings: A mixed-methods feasibility study | 2023 | Mixed-methods study |
| 39 | Country and policy factors influencing the implementation of primary care-based alcohol screening: A comparison of Colombia, Mexico and Peru | 2022 | Mixed-methods study |
| 40 | Influence of the Six-Phase Program on L2 Motivational Self System of Immigrants | 2022 | Insufficient sample size (<25% Peruvian participants) |
| 41 | Perception and experience of obstetric violence in postpartum women at a public hospital in Peru: a mixed study. | 2025 | Mixed-methods study |
| 42 | The advertising discourse of menstrual activism in the femcare industry | 2023 | Not published in a peer-reviewed journal |
| 43 | The impact of the COVID-19 pandemic on patients’ experiences obtaining a tuberculosis diagnosis in Peru: a mixed-methods study | 2022 | Mixed-methods study |
| 44 | Community and hospital academic performance of working nurse interns: A mixed-methods study in Peru | 2023 | Mixed-methods study |
| 45 | Development of a Framework to Assess Challenges to Virtual Education in an Emergency Remote Teaching Environment: A Developing Country Student Perspective—The Case of Peru | 2022 | Mixed-methods study |
| 46 | The Dynamics of Intimate Partner Violence and Its Impact on HIV Care: A Cross-Sectional Study of People of Mixed Gender and Sexual Preference in Lima, Peru | 2022 | Mixed-methods study |
| 47 | Does Access to Point-of-Care Medical Information Improve Trauma and General Surgeons' Clinical Knowledge in a Middle-Income Country? A Mixed-Methods Study with Random Assignment | 2022 | Mixed-methods study |
| 48 | Impact of digitization on educational management: Results of the introduction of a learning management system in a traditional school context | 2025 | Mixed-methods study |
| 49 | An approach to the meaning of love in a child population in Lima, Peru | 2022 | Mixed-methods study |
| 50 | Core outcome sets for trials of interventions to prevent and to treat multimorbidity in adults in low and middle-income countries: The COSMOS study | 2024 | Sistematic Review |
| 51 | Invisible girls and boys, their rights to a healthy environment and a family environment in the Establecimiento Penitenciario de Mujeres Socabaya-Arequipa-Peru | 2024 | Not health-related topic |
| 52 | The impact of research on health education/health literacy on policymaking in Latin America and the Caribbean Region | 2024 | Mixed-methods study |
| 53 | Exploring the Role of the Private Sector in Tuberculosis Detection and Management in Lima, Peru: A Mixed-Methods Patient Pathway Analysis | 2023 | Mixed-methods study |
| 54 | COVID-19's impact on type 1 diabetes management: A mixed-methods study exploring the Peruvian experience | 2022 | Mixed-methods study |
| 55 | Seeds of art: a community intervention based on the principles of expressive arts therapy, developed with migrant women | 2023 | Not health-related topic |
