# Supplementary material for: Reporting quality of qualitative health studies published by Peruvian authors: A scoping review
Source: PLoS One. 2026 Jun 23;21(6):e0351494. doi: 10.1371/journal.pone.0351494 (PMC13289893; doi:10.1371/journal.pone.0351494)
Supplement: Attachment — Submitted filename: PONE-D-26-05648-AF.pdf [file pone.0351494.s005.pdf]

PLOS One

Reporting quality of qualitative health studies published by Peruvian authors: a scoping review

--Manuscript Draft--

**Manuscript Number:**	PONE-D-26-05648
**Article Type:**	Research Article
**Full Title:**	Reporting quality of qualitative health studies published by Peruvian authors: a scoping review
**Short Title:**	Quality of Reporting in Peruvian Qualitative Health Studies
**Corresponding Author:**	Rubí Paredes-Angeles<br>Universidad Cientifica del Sur<br>Lima, PERU
**Keywords:**	qualitative research; Reporting quality; Standards for Reporting Qualitative Research; scoping review; Peru
**Abstract:**	Qualitative research offers valuable insights into complex social and behavioral processes in the health sciences; however, its contribution depends on the quality and transparency of reporting. This scoping review aimed to describe the methodological characteristics and reporting quality of qualitative studies published by Peruvian authors between 2022 and 2025. Following PRISMA-ScR guidelines, a systematic search was conducted in SCOPUS, and reporting quality was assessed using the Standards for Reporting Qualitative Research (SRQR) tool. A total of 147 qualitative studies were included. Most were published in English (49.0%), had a median of five authors; 71.4% had a first author affiliated with a Peruvian institution, and 58.5% involved exclusively Peruvian authors. Studies were mainly conducted in Lima (50.7%). Ethics committee approval was not reported in 24.5% of studies, and 40.8% did not provide an ethics approval code. Adherence to SRQR items was highest for abstracts (95.9%), study purpose (95.2%), and presentation of findings (93.9%), whereas reporting of title elements (31.3%), qualitative approach (49.0%), and researcher reflexivity (37.4%) was limited. Publication in Spanish and Peruvian first authorship were associated with lower SRQR scores, whereas a higher number of authors and international collaboration were associated with higher scores. Overall, qualitative studies published by Peruvian authors showed variable reporting quality, with important methodological and ethical aspects frequently underreported, highlighting the need to strengthen reporting practices, particularly for core qualitative components.
**Order of Authors:**	Cristhian Rojas-Miliano
	Andrea Dámaso-Román
	Guillermo Almeida-Huanca
	Yolanda Viguria-Chavez
	Nikol Mayo-Puchoc
	Andy Rick Sánchez-Villena
	Rubí Paredes-Angeles
	Alvaro Taype-Rondan
**Opposed Reviewers:**	
**Additional Information:**	
**Question**	**Response**
**Financial Disclosure**<br><br>Enter a financial disclosure statement that	The author(s) received no specific funding for this work.

Powered by Editorial Manager® and ProduXion Manager® from Aries Systems Corporation

describes the sources of funding for the work included in this submission. Review the submission guidelines for detailed requirements. View published research articles from *PLOS ONE* for specific examples.<br><br>This statement is required for submission and **will appear in the published article** if the submission is accepted. Please make sure it is accurate.<br><br>**Funded studies**<br>Enter a statement with the following details:<br>• Initials of the authors who received each award<br>• Grant numbers awarded to each author<br>• The full name of each funder<br>• URL of each funder website<br>• Did the sponsors or funders play any role in the study design, data collection and analysis, decision to publish, or preparation of the manuscript?<br><br>Did you receive funding for this work?	
**Competing Interests**<br><br>Use the instructions below to enter a competing interest statement for this submission. On behalf of all authors, disclose any competing interests that could be perceived to bias this work—acknowledging all financial support and any other relevant financial or non-financial competing interests.<br><br>This statement is required for submission and **will appear in the published article** if the submission is accepted. Please make sure it is accurate and that any funding sources listed in your Funding Information later in the submission form are also declared in your Financial Disclosure statement.<br><br>View published research articles from *PLOS ONE* for specific examples.	The authors have declared that no competing interests exist.

**NO authors have competing interests**<br><br>Enter: *The authors have declared that no competing interests exist.*<br><br>**Authors with competing interests**<br><br>Enter competing interest details beginning with this statement:<br><br>*I have read the journal's policy and the authors of this manuscript have the following competing interests: [insert competing interests here]*<br><br>* typeset	
**Ethics Statement**<br><br>Enter an ethics statement for this submission. This statement is required if the study involved:<br><br>• Human participants<br>• Human specimens or tissue<br>• Vertebrate animals or cephalopods<br>• Vertebrate embryos or tissues<br>• Field research<br><br>Write "N/A" if the submission does not require an ethics statement.<br><br>General guidance is provided below. Consult the submission guidelines for detailed instructions. **Make sure that all information entered here is included in the Methods section of the manuscript.**	N/A

Powered by Editorial Manager® and ProduXion Manager® from Aries Systems Corporation

**Format for specific study types**<br><br>**Human Subject Research (involving human participants and/or tissue)**<br>• Give the name of the institutional review board or ethics committee that approved the study<br>• Include the approval number and/or a statement indicating approval of this research<br>• Indicate the form of consent obtained (written/oral) or the reason that consent was not obtained (e.g. the data were analyzed anonymously)<br><br>**Animal Research (involving vertebrate animals, embryos or tissues)**<br>• Provide the name of the Institutional Animal Care and Use Committee (IACUC) or other relevant ethics board that reviewed the study protocol, and indicate whether they approved this research or granted a formal waiver of ethical approval<br>• Include an approval number if one was obtained<br>• If the study involved *non-human primates*, add *additional details* about animal welfare and steps taken to ameliorate suffering<br>• If anesthesia, euthanasia, or any kind of animal sacrifice is part of the study, include briefly which substances and/or methods were applied<br><br>**Field Research**<br><br>Include the following details if this study involves the collection of plant, animal, or other materials from a natural setting:<br>• Field permit number<br>• Name of the institution or relevant body that granted permission	
**Data Availability**<br><br>Authors are required to make all data underlying the findings described fully available, without restriction, and from the time of publication. PLOS allows rare exceptions to address legal and ethical concerns. See the PLOS Data Policy and FAQ for detailed information.	Yes - all data are fully available without restriction

Powered by Editorial Manager® and ProduXion Manager® from Aries Systems Corporation

A Data Availability Statement describing where the data can be found is required at submission. Your answers to this question constitute the Data Availability Statement and **will be published in the article**, if accepted.<br><br>**Important:** Stating 'data available on request from the author' is not sufficient. If your data are only available upon request, select 'No' for the first question and explain your exceptional situation in the text box.<br><br>Do the authors confirm that all data underlying the findings described in their manuscript are fully available without restriction?	
**Describe where the data may be found in full sentences. If you are copying our sample text, replace any instances of XXX with the appropriate details.**<br><br>• If the data are **held or will be held in a public repository**, include URLs, accession numbers or DOIs. If this information will only be available after acceptance, indicate this by ticking the box below. For example: *All XXX files are available from the XXX database (accession number(s) XXX, XXX.*).<br>• If the data are all contained **within the manuscript and/or Supporting Information files**, enter the following: *All relevant data are within the manuscript and its Supporting Information files.*<br>• If neither of these applies but you are able to provide **details of access elsewhere**, with or without limitations, please do so. For example:<br><br>*Data cannot be shared publicly because of [XXX]. Data are available from the XXX Institutional Data Access / Ethics Committee (contact via XXX) for researchers who meet the criteria for access to confidential data.*<br><br>*The data underlying the results presented in the study are available from (include the name of the third party*	All relevant data are within the manuscript and its Supporting Information files.

and contact information or URL).<br>• This text is appropriate if the data are owned by a third party and authors do not have permission to share the data.<br><br>* typeset	
Additional data availability information:	

COVER LETTER

Editor-in-Chief
Plos One

Dear Editor,

We are pleased to submit our manuscript entitled: **"Reporting quality of qualitative health studies published by Peruvian authors: a scoping review"** for consideration for publication in Plos One.

This manuscript is a scoping review that evaluates the reporting quality of qualitative health research conducted in the Peruvian population, using the Standards for Reporting Qualitative Research (SRQR) as an assessment framework. While prior studies have examined reporting quality in quantitative or mixed-methods research in Latin America, evidence focusing specifically on qualitative health research and its reporting practices remains scarce. Our study addresses this gap by providing a structured and comprehensive overview of current reporting practices among Peruvian qualitative studies.

Our findings indicate that although general methodological components are frequently reported, elements that are specific and essential to qualitative research—such as researcher reflexivity, analytic processes, and contextual positioning—are often underreported. Additionally, a substantial proportion of studies did not report approval by a research ethics committee. We further explored factors associated with reporting quality, including publication language, first author affiliation, number of authors, and international collaboration, thereby contributing novel insights into structural and contextual influences on reporting practices.

We believe that these findings contribute meaningfully to the existing literature by identifying recurring deficiencies in qualitative research reporting and offering evidence that may inform researchers, editors, and reviewers. Although the study focuses on Peru, the challenges identified may reflect similar issues in countries with comparable research environments.

We confirm that this manuscript is original, is not under consideration by any other journal, and that all authors have approved its submission. There has been no prior interaction with PLOS or this journal regarding this manuscript.

Thank you for your time and consideration. We appreciate the opportunity to submit our work and look forward to your response.

Sincerely,

Rubí Paredes-Angeles
Corresponding author
Universidad Científica del Sur, Lima, Peru

Manuscript

Click here to access/download;Manuscript;1. Full-text Final Version.docx

Reporting quality of qualitative health studies published by Peruvian authors: a scoping review

Authors

Cristhian Rojas-Miliano* (1,2)

alexrojasmiliano@gmail.com https://orcid.org/0000-0002-3364-4940

Andrea Dámaso-Román (3)

andrea.damaso@unmsm.edu.pe https://orcid.org/0000-0002-8150-5777

Guillermo Almeida-Huanca (4)

guillermoalmeida1604@gmail.com https://orcid.org/0000-0003-0283-3586

Nikol Mayo-Puchoc (5)

nikol.mayo@upch.pe https://orcid.org/0000-0002-6182-7605

Yolanda Viguria-Chavez (6)

yolanda.viguria.c@upch.pe https://orcid.org/0000-0002-3339-112X

Andy Rick Sánchez-Villena (7)

andysavi92@gmail.com https://orcid.org/0000-0001-7828-5884

Alvaro Taype-Rondan (8,9)

alvaro.taype.r@gmail.com https://orcid.org/0000-0001-8758-0463

Rubí Paredes-Angeles (10)

rubiparedesa@gmail.com https://orcid.org/0000-0003-3669-4932

Affiliations

1) Universidad Nacional del Centro del Perú, Junín, Perú
2) Centro de Salud Mental Comunitario Pangoa, Junín, Perú

3) Universidad Nacional Mayor de San Marcos, Facultad de Psicología, Lima, Perú
4) Grupo de Investigación en Psicología Social y Desarrollo Humano, Facultad de Psicología, Universidad Nacional Mayor de San Marcos, Lima, Perú
5) Instituto Peruano de Orientación Psicológica, Lima, Perú
6) Universidad Peruana Cayetano Heredia, School of Public Health, Lima, Perú
7) Universidad Señor de Sipán, Vicerrectorado de Investigación, Chiclayo, Perú
8) Unidad de Investigación para la Generación y Síntesis de Evidencias en Salud, Vicerrectorado de Investigación, Universidad San Ignacio de Loyola, Lima, Perú
9) EviSalud – Evidencias en Salud, Lima, Perú
10) Universidad Científica del Sur, Lima, Perú

***Corresponding author:**

Rubi Paredes-Angeles

Phone number: +51 964772360

Mail: rubiparedesa@gmail.com

Universidad Científica del Sur, Lima, Peru

Author contributions:

Conceptualization: Rubi Paredes-Angeles, Alvaro Taype-Rondan

Data Curation: Cristhian Rojas-Miliano, Andrea Dámaso-Román, Guillermo Almeida-Huanca, Nikol Mayo-Puchoc, Yolanda Viguria-Chavez, Andy Rick Sánchez-Villena

Formal Analysis: Rubi Paredes-Angeles

Investigation: Cristhian Rojas-Miliano, Andrea Dámaso-Román, Guillermo Almeida-Huanca, Nikol Mayo-Puchoc, Yolanda Viguria-Chavez, Andy Rick Sánchez-Villena

Methodology: Rubi Paredes-Angeles Alvaro Taype-Rondan

Writing – Original Draft Preparation: Cristhian Rojas-Miliano, Andrea Dámaso-Román, Guillermo Almeida-Huanca, Nikol Mayo-Puchoc, Yolanda Viguria-Chavez, Andy Rick Sánchez-Villena, Rubi Paredes-Angeles Alvaro Taype-Rondan

Writing – Review & Editing: Cristhian Rojas-Miliano, Andrea Dámaso-Román, Guillermo Almeida-Huanca, Nikol Mayo-Puchoc, Yolanda Viguria-Chavez, Andy Rick Sánchez-Villena, Rubi Paredes-Angeles Alvaro Taype-Rondan

Abstract

Qualitative research offers valuable insights into complex social and behavioral processes in the health sciences; however, its contribution depends on the quality and transparency of reporting. This scoping review aimed to describe the methodological characteristics and reporting quality of qualitative studies published by Peruvian authors between 2022 and 2025. Following PRISMA-ScR guidelines, a systematic search was conducted in SCOPUS, and reporting quality was assessed using the Standards for Reporting Qualitative Research (SRQR) tool. A total of 147 qualitative studies were included. Most were published in English (49.0%), had a median of five authors; 71.4% had a first author affiliated with a Peruvian institution, and 58.5% involved exclusively Peruvian authors. Studies were mainly conducted in Lima (50.7%). Ethics committee approval was not reported in 24.5% of studies, and 40.8% did not provide an ethics approval code. Adherence to SRQR items was highest for abstracts (95.9%), study purpose (95.2%), and presentation of findings (93.9%), whereas reporting of title elements (31.3%), qualitative approach (49.0%), and researcher reflexivity (37.4%) was limited. Publication in Spanish and Peruvian first authorship were associated with lower SRQR scores, whereas a higher number of authors and international collaboration were associated with higher scores.

Overall, qualitative studies published by Peruvian authors showed variable reporting quality, with important methodological and ethical aspects frequently underreported, highlighting the need to strengthen reporting practices, particularly for core qualitative components.

Key words: Qualitative Research; Reporting quality; Standards for Reporting Qualitative Research; Scoping review; Peru.

Introduction

Qualitative research provides a deeper understanding of complex social and behavioral processes in health sciences [illegible]Unlike the quantitative methods, qualitative studies seek to explore, narrate and explain experiences, perceptions, and contexts, offering a transformative perspective that enriches evidence-based decision-making [2,3]. Thus, qualitative research enhances the contextual understanding necessary for developing grounded health interventions [1,2].

However, the usefulness of qualitative findings can be affected by the quality of reporting [4]. Although the inherent heterogeneity of qualitative research reporting reflects its richness and diversity, this variability can also impede systematic evaluation of quality and comparison across studies [5]. Clear, complete, and transparent reporting enables readers to better assess the credibility, transferability, and methodological rigor of qualitative studies.

Some international guidelines have been developed to standardize reporting in qualitative research, including the Standards for Reporting Qualitative Research (SRQR) [6], the Consolidated Criteria for Reporting Qualitative Research (COREQ) [7], and the Enhancing Transparency in Reporting the synthesis of Qualitative research (ENTREQ) [8]. Unlike COREQ, which is tailored for interviews and focus groups, and ENTREQ, which is focused on qualitative synthesis studies, SRQR provides a comprehensive framework that authors can use across a wide range of qualitative methodologies [6,9]. Thus, previous research has employed the SRQR to evaluate reporting quality in qualitative studies conducted in diverse health domains, including urology [10], pediatrics [11], infectious diseases [12], and breast cancer [13].

In Peru, health research publications have increased [14]; however, to date, no studies have specifically examined qualitative health research or assessed the quality of its reporting. The absence of such information limits understanding of the transparency and reporting standards within this research field in Peru. Assessing these aspects may help identify strengths, weaknesses, and opportunities for improvement among researchers, reviewers, and editors of scientific journals [15]. Therefore, this study aimed to describe the methodological characteristics and assess the reporting quality of qualitative studies published by Peruvian authors.

Methods

This scoping review was carried out and reported following the guidelines of the Preferred Reporting Items for Systematic Reviews and Meta-Analyses Extension for Scoping Reviews (PRISMA-ScR)[16]. The completed PRISMA-ScR checklist is provided in **Supplementary Material 1**. A detailed study protocol was developed prior to study initiation and made publicly available. The protocol of this review is available at https://osf.io/jes6v/files/3rn52.

Search strategy

A systematic search was performed in Scopus on October 15, 2025, using the following search strategy to identify qualitative health studies authored by researchers affiliated with institutions in Peru and published between 2022 and 2025:

AFFILCOUNTRY (peru) AND TITLE-ABS (qualitative) AND ((SUBJAREA (MEDI OR NURS OR DENT OR HEAL) OR SUBJAREA (PSYC))) AND PUBYEAR>2021 AND PUBYEAR < 2026

Study eligibility

We included original articles authored by researchers affiliated with institutions in Peru, that were published in scientific journals between 2022 and 2025 and presented findings from qualitative studies. This timeframe was chosen to reflect the current state of the field.

Only studies in which at least 25% of participants were based in Peru were considered. Studies related to health disciplines, including medicine, nursing, dentistry, psychology, and other health professions, were included. Mixed-methods studies were excluded due to the need for specific assessment tools. No language restrictions were applied.

Studies selection

Records were uploaded to the “Rayyan” software. Six authors in pairs independently screened the records applying the selection criteria. Disagreements between the reviewers were resolved through discussion among the reviewers or with the collaboration of another authors.

Data extraction

To ensure consistency, a pilot data extraction was conducted prior to the main process. Subsequently, six authors worked in pairs to independently extract data from each study, recording all information in a predesigned Excel spreadsheet. Any discrepancies were reviewed and resolved with input from a fifth author. The research team consisted of professionals in Medicine and Psychology with experience in conducting health research.

For each included study, we collected information on its general and methodological characteristics, including: language of the study (English, Spanish, or English and

Spanish), whether the study was published in a Peruvian journal (yes or no), number of authors (divided in tertiles), country of the first author's institutional affiliation, international collaboration, defined as the inclusion of at least one co-author with an institutional affiliation in a country other than Peru (yes or no), study population, sample size, study setting (community settings, health facilities or educational centers), Peruvian region where the study was conducted, multicountry study, defined as a study that included research sites in countries other than Peru (only Peru or Peru and others countries), ethics committee approval (whether the article reported approval from at least one ethics committee), and ethics approval code reported (whether the article, supplementary material, or appendix included the ethics approval code). Additionally, we assessed the quality of reporting using the SRQR checklist.

Quality of reporting

We used the SRQR 21-item checklist [6], which provides guidance on transparency and completeness in qualitative research reporting. Each of the 21 items was assessed to determine whether it was 'reported' (score = 2 points), 'partially reported' (score = 1 point), or 'not reported' (score = 0 points).

The total SRQR score ranged from 0 to 42 points. An item was considered 'reported' when the authors presented the required information in accordance with the SRQR guidelines, 'partially reported' when the authors provided some, but not all, of the required information, and 'not reported' when the authors failed to provide any of the required information according to the SRQR reporting standard [9]. Also, we created the variable "adequate SRQR reporting," defined as belonging to the highest tertile (≥ 37 points) of the SRQR score.

This SRQR checklist assessed the following items:

S1. Title. Concise description of the nature and topic of the study. Identifying the study as qualitative/mixed or indicating the approach (e.g., ethnography, grounded theory) or data collection methods (e.g., interview, focus group) is recommended.

S2. Abstract. Summary of key elements of the study using the abstract format of the intended publication; typically includes background, purpose, methods, results, and conclusions.

S3. Introduction. Description and significance of the problem/phenomenon studied; review of relevant theory and empirical work problem statement.

S4. Introduction. Purpose of the study and specific objectives or questions.

S5. Methods. Qualitative approach (e.g., ethnography, grounded theory, case study, phenomenology, narrative research) and guiding theory if appropriate; identifying the research paradigm (e.g., postpositivist, constructivist/ interpretivist) is also recommended; rationale.

S6. Methods. Researchers' characteristics that may influence the research, including personal attributes, qualifications/experience, relationship with participants, assumptions, and/or presuppositions; potential or actual interaction between researchers' characteristics and the research questions, approach, methods, results, and/or transferability.

S7. Methods. Context. Setting/site and salient contextual factors; rationale.

S8. Methods. Sampling strategy. How and why research participants, documents, or events were selected; criteria for deciding when no further sampling was necessary (e.g., sampling saturation); rationale

S9. Methods. Documentation of approval by an appropriate ethics review board and participant consent, or explanation for lack thereof; other confidentiality and data security issues

S10. Methods. Types of data collected; details of data collection procedures including (as appropriate) start and stop dates of data collection and analysis, iterative process, triangulation of sources/methods, and modification of procedures in response to evolving study findings; rationale

S11. Methods. Description of instruments (e.g., interview guides, questionnaires) and devices (e.g., audio recorders) used for data collection; if/how the instrument(s) changed over the course of the study

S12. Methods (or results). Number and relevant characteristics of participants, documents, or events included in the study; level of participation (could be reported in results)

S13. Methods. Methods for processing data prior to and during analysis, including transcription, data entry, data management and security, verification of data integrity, data coding, and anonymization/deidentification of excerpts"

S14. Methods. Process by which inferences, themes, etc., were identified and developed, including the researchers involved in data analysis; usually references a specific paradigm or approach; rationale

S15. Methods. Techniques to enhance trustworthiness and credibility of data analysis (e.g., member checking, audit trail, triangulation); rationale

S16. Results. Main findings (e.g., interpretations, inferences, and themes); might include development of a theory or model, or integration with prior research or theory

S17. Results. Evidence (e.g., quotes, field notes, text excerpts, photographs) to substantiate analytic findings

S18. Discussion. Short summary of main findings; explanation of how findings and conclusions connect to, support, elaborate on, or challenge conclusions of earlier scholarship; discussion of scope of application/generalizability; identification of unique contribution(s) to scholarship in a discipline or field

S19. Discussion. Trustworthiness and limitations of findings

S20. Conflicts of interest. Potential sources of influence or perceived influence on study conduct and conclusions; how these were managed

S21. Funding. Sources of funding and other support; role of funders in data collection, interpretation, and reporting.

Data analysis

Data were entered into Microsoft Excel, and all statistical analyses were performed using Stata v19.0 software. Descriptive analyses included absolute and relative frequencies for categorical variables and medians with interquartile ranges for numeric variables. Additionally, an exploratory analysis of factors associated with adequate SRQR reporting was conducted. For this purpose, crude prevalence ratios (PR) were estimated using Poisson regression with robust variance.

Ethical statement:

Ethics committee approval was not required because this study involved only a review of previously published literature and did not include human participants.

Results

The search yielded 489 records, of which 202 were selected for full-text assessment after duplicate removal and title and abstract screening. Of these, 55 reports were excluded for the following reasons: mixed-methods design (n = 46), non–health-related topic (n = 4), publication outside a peer-reviewed journal (n = 2), unspecified Peruvian population size (n = 1), inclusion of less than 25% Peruvian participants (n = 1), and systematic review design (n = 1). Consequently, a total of 147 studies were included in the final analysis (**Figure 1**). The characteristics of the included studies can be found in **Supplementary Material 2**, while the list of excluded reports is presented in **Supplementary Material 3**.

Figure 1. Flowchart of the article selection process.

Across the included studies, most were published in English (49.0%) and were not published in a Peruvian journal (88.4%). The median number of authors was five, and 37.4% were studies with four to seven authors. Regarding affiliation, the first author most frequently had their primary institutional affiliation in Peru (71.4%), and most studies involved only Peruvian authors (58.5%). The most frequent main population studied were health professionals (20.4%) and non-clinical stakeholders (20.4%). The majority of studies were conducted in community settings (42.2%). Most studies included only participants from Peru (91.8%). Ethical procedures were not reported in 24.5% of the studies, and 40.8% did not provide an ethics approval code **(Table 1).**

Table 1. Characteristics of included studies (n=147).

Characteristics	n (%)
Language	
English	72 (49.0)
Spanish	63 (42.8)
English and Spanish	12 (8.2)
Published in a Peruvian journal	
No	130 (88.4)
Yes	17 (11.6)
Number of authors*	5 [4 - 8]
1 to 4	53 (36.1)
4 to 7	55 (37.4)
8 to 36	39 (26.5)
Country of the first author's first affiliation	
Peru	105 (71.4)
United States	27 (18.4)
United Kingdom	4 (2.7)
Switzerland	3 (2.0)
Spain	2 (1.4)
Canada	2 (1.4)
Other countries	4 (2.7)
International collaboration	
Only authors from Peru	86 (58.5)
At least one international author	61 (41.5)
Main population of the study	
Children, adolescents, and young people	21 (14.3)
Mothers, fathers, caregivers, and families	22 (15.0)
Indigenous peoples, individuals from rural communities, and culturally specific groups	19 (12.9)
Patients, service users, and individuals living with health conditions	25 (17.0)
Health professionals (clinicians and care personnel)	30 (20.4)
Policy makers, administrators, program managers, community leaders, stakeholders, and other non-clinical populations	30 (20.4)
Sample size*	15 [12 - 30]
Study setting	
Not reported	1 (0.7)
Community settings	62 (42.2)
Health facilities	46 (31.2)
Educational centers	20 (13.6)
Others	18 (12.3)

Region where the study was conducted†	
Not reported	13 (8.8)
Lima	75 (50.3)
Lambayeque	29 (19.5)
Loreto	10 (6.8)
Junin	9 (6.1)
La Libertad	6 (4.1)
Others	37 (25.0)
Multicountry sample	
Peru only	135 (91.8)
Peru and other countries	12 (8.2)
Reported the ethics committee approval	
No	36 (24.5)
Yes	111 (75.5)
Reported the ethics approval code	
No	60 (40.8)
Yes	87 (59.2)
Total SRQR (in points)*	31 [34 - 38]
Adequate reporting	
No	99 (67.3)
Yes	48 (32.7)

SRQR: Standards for Reporting Qualitative Research.
*Median [p25 - p75]
†For this variable, the percentages add up to more than 100% because some studies were conducted in more than one Peruvian region.

The highest number of qualitative studies were identified in Lima and Lambayeque, with 75 (50.3%) and 29 (19.5%) studies, respectively, while no studies were identified in Apurímac, Huancavelica, and Tumbes **(Figure 2)**.

Figure 2. Number of qualitative studies conducted by region.

The SRQR items with the highest level of adherence were the abstract (S2), reported by 95.9% of studies, the statement of study purpose (S4), reported by 95.2%, and the presentation of main findings (S16), reported by 93.9%. On the other hand, only 31.3% of studies adequately reported the title elements (S1). Reporting of the qualitative

approach or guiding paradigm (S5) was also limited, with 49.0% of studies meeting this criterion. Similarly, researchers' characteristics and reflexivity (S6) were reported in just 37.4% of studies **(Figure 3).**

Figure 3. Fulfillment of the SRQR items among Peruvian qualitative studies (n=147).

This is the Figure 1 legend

SRQR: Standards for Reporting Qualitative Research
S1. Quality of title
S2. Quality of abstract
S3. Introduction. Background and problem description
S4. Introduction. Study purpose and objectives
S5. Methods: Qualitative approach and research paradigm
S6. Methods: Researcher characteristics and reflexivity
S7. Methods: Study context and setting
S8. Methods: Sampling strategy
S9. Methods: Ethical approval and consent
S10. Methods: Data collection procedures
S11. Methods: Data collection instruments
S12. Methods: Participant characteristics
S13. Methods: Data processing and management
S14. Methods: Data analysis process
S15. Methods: Trustworthiness and credibility techniques
S16. Results: Main findings
S17. Results: Supporting evidence for findings
S18. Discussion: Interpretation and contribution to literature
S19. Discussion: Limitations and trustworthiness
S20. Conflicts of interest
S21. Funding sources

In the regression analysis, some study characteristics were associated with a higher prevalence of adequate SRQR reporting (score $\geq$ 37). Studies published in Spanish showed a lower frequence of adequate reporting compared with those published in English (PR = 0.12; 95% CI: 0.05–0.32). Studies with eight or more authors (PR = 3.47; 95% CI: 1.81–6.67), studies whose first author was affiliated with a non-Peruvian institution (PR = 0.40; 95% CI: 0.26–0.62), and studies involving international

collaboration (PR = 2.35; 95% CI: 1.45–3.82), had a higher prevalence of adequate reporting. In contrast, neither the main population of interest nor publication in a Peruvian journal showed a statistically significant association (**Table 2**).

Table 2. Associated factors with adequate SRQR checklist reporting (n = 147).

Variables	Adequate SRQR reporting		RP (IC 95%)
	No (SRQR score < 37)	Yes (SRQR score ≥ 37)	
Language of the journal			
English	40 (47.6)	44 (52.4)	Ref.
Spanish	59 (93.7)	4 (6.3)	**0.12 (0.05 to 0.32)**
Published in a Peruvian journal			
No	87 (66.9)	43 (33.1)	Ref.
Yes	12 (70.6)	5 (29.4)	0.89 (0.41 to 1.94)
Number of authors			
1 to 4	44 (83.0)	9 (17.0)	Ref.
4 to 7	39 (70.9)	16 (29.1)	1.71 (0.83 to 3.54)
8 to 36	16 (41.0)	23 (59.0)	**3.47 (1.81 to 6.67)**
Country of the first author's first affiliation			
Non Peru	18 (42.9)	24 (57.1)	Ref
Peru	81 (77.1)	24 (22.9)	**0.40 (0.26 to 0.62)**
International collaboration			
Only authors from Peru	68 (79.1)	18 (20.9)	Ref.
At least one international author	31 (50.8)	30 (49.2)	**2.35 (1.45 to 3.82)**
Main population of the study			
Children, adolescents, and young people	16 (76.2)	5 (23.8)	Ref.

Mothers, fathers, caregivers, and families	25 (83.3)	5 (16.7)	0.70 (0.23 to 2.13)
Indigenous peoples, individuals from rural communities, and culturally specific groups	12 (57.1)	9 (42.9)	1.80 (0.72 to 4.49)
Patients, service users, and individuals living with health conditions	14 (56.0)	11 (44.0)	1.85 (0.76 to 4.49)
Health professionals (clinicians and care personnel)	11 (55.0)	9 (45.0)	1.89 (0.76 to 4.69)
Policy makers, administrators, program managers, community leaders, stakeholders, and other non-clinical populations	21 (70.0)	9 (30.0)	1.26 (0.49 to 3.24)

SRQR: Standards for Reporting Qualitative Research.
PR: Prevalence ratio. 95% CI: 95% confidence interval. Ref: reference.

Discussion

Main results

A total of 147 qualitative studies were included, most of which were published in English, had a median of five authors, and were primarily conducted in Lima and community settings. First authors were most commonly affiliated with Peruvian institutions as their primary affiliation (71.4%), and the majority of studies were conducted exclusively by Peruvian authors (58.5%). Moreover, ethics committee approval was not reported in 24.5% of the studies, and 40.8% did not provide an ethics approval code. The SRQR items with the highest adherence were the abstract (S2), the statement of study purpose (S4), and the presentation of main findings (S16), whereas title elements (S1), the qualitative approach or guiding paradigm (S5), and researchers' characteristics and reflexivity (S6) were less frequently reported. Adequate SRQR reporting was associated with publication in English, a higher number of authors, non-Peruvian first-author

affiliation, and international collaboration, with no significant associations observed for the study population or publication in Peruvian journals.

Characteristics of the studies

Approximately half of the included studies were conducted in Lima, indicating a pronounced geographical concentration of qualitative research in Peru, consistent with prior evidence of higher scientific output in Lima, the capital of Peru [17,18]. This finding suggests that qualitative research follows the pattern of centralized scientific activity in Peru. Barriers to research production in other regions may be driven by structural inequities, such as limited access to research training and funding, as well as the concentration of institutions with greater research capacity in Lima [17]. Strengthening interregional collaboration, revising funding allocation mechanisms, and prioritizing equitable research capacity development may contribute to a more balanced national research landscape [19].

Nearly one quarter of the studies did not report ethical procedures, and over two fifths failed to include an ethics approval code. This finding may reflect two non-mutually exclusive explanations. On the one hand, some studies may not have been reviewed by an ethics committee, which is particularly concerning given that qualitative research typically involves close engagement with participants and may address sensitive topics, thereby requiring ethical oversight to safeguard participant welfare, informed consent, and confidentiality. On the other hand, these results may indicate shortcomings in reporting practices rather than deficiencies in ethical conduct, whereby ethical approval was obtained but not explicitly documented in the published reports.

Well-established ethical and regulatory frameworks, including the Declaration of Helsinki and the International Ethical Guidelines for Health-related Research Involving Humans issued by CIOMS, as well as current Peruvian regulations [20], stipulate that all

research involving human participants must undergo prior review and approval by a research ethics committee. These provisions aim to safeguard participants' rights, safety, and well-being, and to ensure adherence to fundamental ethical principles such as autonomy, confidentiality, and justice.

Although the first author's primary institutional affiliation was predominantly Peruvian (71.4%), a substantial proportion of the studies involved international collaboration (41.5%). This pattern suggests that, despite local leadership in qualitative research production, international collaboration could play a meaningful role in the development of these studies. Such collaborations may reflect efforts to strengthen methodological rigor, access complementary expertise, and enhance the visibility of research conducted in Peru.

Results of the SRQR checklist

The items most consistently reported included the presence of an abstract containing the key elements of the study (S2), the specification of the study objective in the introduction (S4), and the reporting of results, particularly the description of the main findings and the evidence supporting them (S16 and S17). In contrast, the least frequently addressed elements were the use of techniques to ensure the credibility of the findings (S15), the qualitative approach and research paradigm (S5), the characteristics of the researchers (S6), and the formulation of a title that accurately reflects the nature and focus of the study (S1).

Our findings align with a previous study of qualitative research in pediatric urology, where the abstract and the study objective were also among the most consistently reported

items [10]. Moreover, our results are consistent with a previous research on qualitative studies of malaria in Colombia, which identified credibility techniques and the description of the methodological paradigm as among the least reported elements [12]. Another study examining qualitative research in community pharmacies likewise documented poor reporting of the title, researcher characteristics, and the nature of the study [9]. However, unlike those studies, which focused on specific populations or thematic areas, our analysis encompasses the qualitative scientific output of an entire region. This convergence suggests that there is a persistent tendency to report items that are commonly required in scientific manuscripts, irrespective of study design (e.g., abstract key aspects, objective, main findings), reflecting adherence to general reporting conventions rather than specific compliance with qualitative reporting guidelines. By contrast, several items that are fundamental to the rigor and transparency of qualitative research were among the least frequently reported.

This pattern indicates that while general reporting standards are largely met, less emphasis is placed on elements that are distinctive to qualitative inquiry and essential for the appraisal of methodological rigor. In this context, strengthening training in qualitative research reporting, encouraging the adoption of reporting guidelines such as SRQR by journals, and ensuring specialized peer review processes are essential to improve reporting quality.

Factors associated with adequate SRQR reporting

Regarding associated factors, articles published in Spanish were associated with lower reporting quality scores. This may be explained by the tendency of non-English-speaking authors to prioritize publication in English due to its greater international visibility [21] and citation potential [22]. As a result, manuscripts with stronger methodological rigor and

better adherence to reporting standards may be more likely to be submitted to, and accepted by, English-language journals, which could partially explain the observed association.

Additionally, lower reporting quality was observed in studies whose first author was affiliated with a Peruvian institution, whereas higher reporting quality was found in studies with a larger number of authors and at least one internationally affiliated author. These findings can be jointly interpreted within the context of international collaboration and may reflect the relevance of international collaboration in achieving higher reporting standards in Peruvian qualitative research. Previous evidence suggests that international collaboration has a positive impact on research quality [23,24], particularly in less well-established research systems [25], such as Peru.

Limitations and strengths

This study has some limitations. First, mixed-methods studies were not included, as their methodological quality is assessed using specific appraisal tools, such as the Mixed Methods Appraisal Tool (MMAT), which fall outside the scope of the present evaluation [26]. Second, the search was restricted to Scopus, excluding regional databases such as SciELO or LILACS due to their limitation in filtering results by affiliation in these repositories. Although Scopus allows the identification of studies with affiliation with Peruvian institutions, this restriction may have omitted qualitative research published in journals with a local scope. Third, the analysis was limited to the 2022–2025 period, to focus on recent studies and reduce potential bias related to the accelerated publication processes during the COVID-19 pandemic [27]. While this timeframe may have limited the inclusion of relevant scientific production, this scope allows for an evaluation of the current state of qualitative research in Peru. Finally, because the SRQR evaluates

reporting quality rather than intrinsic methodological rigor, some studies may have received higher or lower scores based on reporting completeness rather than the strength of their design.

On the other hand, this study also has some strengths. First, flexibility was applied in the interpretation of certain SRQR items to avoid ratings being influenced by editorial decisions rather than the absence of relevant content. For instance, some articles did not present a structured abstract (assessed under item 2) yet contained all expected elements and were therefore awarded full points. Similarly, statements regarding reflexivity and ethical considerations located outside the methods section were accepted as valid. Second, study selection, data extraction, and analysis were conducted independently by pairs of reviewers, with discrepancies resolved by a third or even fourth evaluator, thereby reducing the risk of individual bias and strengthening the reliability of the assessment process. Third, for data extraction, each item was assigned a score, and this score was accompanied by a justification, which allowed for greater discussion among the authors and increased rigor in the assignment of the scores. Finally, the use of a widely recognized instrument such as the SRQR, whose structure allows for the assessment of items applicable across multiple methodological approaches within qualitative research [28].

Conclusion

The study included 147 qualitative studies, most of which were published in English, conducted in Lima and community settings, and developed exclusively by Peruvian authors, with first authors primarily affiliated with Peruvian institutions. Nearly one quarter did not report ethics committee approval, and the least frequently reported SRQR items were title elements, the qualitative approach, and researchers' characteristics and reflexivity. Adequate SRQR reporting was associated with publication in English, a

higher number of authors, non-Peruvian first-author affiliation, and international collaboration.

Funding statement:

This study was self-funded by the authors. YVC's time was funded by the Neuropsychiatry and Behavioral Disorders in First Nations Multidisciplinary Training Program (NEUFIN) grant, NIMH D43MH108169.

Ethical statement:

Ethics committee approval was not required because this study involved only a review of previously published literature and did not include human participants.

Conflict of interest statement:

The authors declare no conflicts of interest related to this study.

Acknowledgments:

None.

Data availability statement:

The datasets generated and/or analyzed during the present study are available from the corresponding author upon reasonable request.

References

1. Kuper A, Reeves S, Levinson W. An introduction to reading and appraising qualitative research. *BMJ*. 2008;337:a288. doi:10.1136/bmj.a288

2. Huston P, Rowan M. Qualitative studies. Their role in medical research. *Can Fam Physician Med Fam Can*. 1998;44:2453-2458.

3. Renjith V, Yesodharan R, Noronha JA, Ladd E, George A. Qualitative Methods in Health Care Research. *Int J Prev Med*. 2021;12:20. doi:10.4103/ijpvm.IJPVM_321_19

4. Dunt D, McKenzie R. Improving the quality of qualitative studies: do reporting guidelines have a place? *Fam Pract*. 2012;29(4):367-369. doi:10.1093/fampra/cms041

5. Mays N, Pope C. Qualitative research in health care. Assessing quality in qualitative research. *BMJ*. 2000;320(7226):50-52. doi:10.1136/bmj.320.7226.50

6. O'Brien BC, Harris IB, Beckman TJ, Reed DA, Cook DA. Standards for reporting qualitative research: a synthesis of recommendations. *Acad Med J Assoc Am Med Coll*. 2014;89(9):1245-1251. doi:10.1097/ACM.0000000000000388

7. Tong A, Sainsbury P, Craig J. Consolidated criteria for reporting qualitative research (COREQ): a 32-item checklist for interviews and focus groups. *Int J Qual Health Care J Int Soc Qual Health Care*. 2007;19(6):349-357. doi:10.1093/intqhc/mzm042

8. Tong A, Flemming K, McInnes E, Oliver S, Craig J. Enhancing transparency in reporting the synthesis of qualitative research: ENTREQ. *BMC Med Res Methodol*. 2012;12:181. doi:10.1186/1471-2288-12-181

9. Aref HAT, Witry M, Olufemi-Yusuf D, Guirguis LM. Ensuring quality qualitative research reporting in community pharmacy: a systematic literature review. *Int J Pharm Pract*. 2021;29(5):416-427. doi:10.1093/ijpp/riab027

10. McCloskey K, Neuzil K, Basak R, Chan KH. Quality of reporting for qualitative studies in pediatric urology—A scoping review. *J Pediatr Urol*. 2023;19(5):643-651. doi:10.1016/j.jpurol.2023.04.027

11. Silva GS, Fernandes D de RF, Alves CRL. Avaliação da assistência à saúde da criança na Atenção Primária no Brasil: revisão sistemática de métodos e resultados. *Ciênc Saúde Coletiva*. 2020;25:3185-3200. doi:https://doi.org/10.1590/1413-81232020258.27512018

12. Cardona-Arias JA, Salas-Zapata W, Carmona-Fonseca J. Systematic review of qualitative studies about malaria in Colombia. *Heliyon*. 2020;6(5). doi:10.1016/j.heliyon.2020.e03964

13. Angarita FA, Zhang Y, Elmi M, Hong NJL. Older women's experience with breast cancer treatment: A systematic review of qualitative literature. *The Breast*. 2020;54:293-302. doi:10.1016/j.breast.2020.11.009

14. Mendoza-Chuctaya G, Chachaima-Mar JE, Mejia CR, et al. Análisis de producción, impacto y redes de colaboración en investigaciones científicas en Scopus en Perú de 2000 a 2019. *Medwave*. 2021;21(2). doi:10.5867/medwave.2021.02.8121

15. Watson A, Jackson D. The Importance of Analytic Integrity and Reporting Guidelines in Reporting Qualitative Research. *J Adv Nurs*. 2025;81(7):3425-3427. doi:10.1111/jan.16617

16. Tricco AC, Lillie E, Zarin W, et al. PRISMA Extension for Scoping Reviews (PRISMA-ScR): Checklist and Explanation. *Ann Intern Med*. 2018;169(7):467-473. doi:10.7326/M18-0850

17. Arcila-Diaz J, Delgado-Caramutti J, Millones-Gómez PA, Figueroa-Quiñones J, Valencia-Arias A. Research trends in Peruvian universities: proposal for a research agenda with a bibliometric approach. *Scientometrics*. 2025;130(1):489-514. doi:10.1007/s11192-024-05211-z

18. Vásquez-Uriarte K, Roque-Henriquez JC, Angulo-Bazán Y, Ninatanta Ortiz JA. Análisis bibliométrico de la producción científica peruana sobre la COVID-19. *Rev Peru Med Exp Salud Pública*. 2021;38:224-231. doi:https://doi.org/10.17843/rpmesp.2021.382.7470

19. Choquez-Millan MF, Lechtape CL, Löhr K, Schröter B, Graef F. Uncovering power asymmetries in North-South research collaborations – An example from sustainability research in Tanzania. *Futures*. 2024;156:103316. doi:10.1016/j.futures.2023.103316

20. Resolución Ministerial N.° 233-2020-MINSA. Accessed January 8, 2026. https://www.gob.pe/institucion/minsa/normas-legales/541139-233-2020-minsa

21. Stockemer D, Wigginton MJ. Publishing in English or another language: An inclusive study of scholar's language publication preferences in the natural, social and interdisciplinary sciences. *Scientometrics*. 2019;118(2):645-652. doi:10.1007/s11192-018-2987-0

22. Di Bitetti MS, Ferreras JA. Publish (in English) or perish: The effect on citation rate of using languages other than English in scientific publications. *Ambio*. 2017;46(1):121-127. doi:10.1007/s13280-016-0820-7

23. Kohus Z, Demeter M, Szigeti GP, Kun L, Lukács E, Czakó K. The Influence of International Collaboration on the Scientific Impact in V4 Countries. *Publications*. 2022;10(4):1-13.

24. Wang J, Frietsch R, Neuhäusler P, Hooi R. International collaboration leading to high citations: Global impact or home country effect? *J Informetr*. 2024;18(4):101565. doi:10.1016/j.joi.2024.101565

25. Farias Borges LF, Fischer B, Mazoni A, Bascur JP. The complex interplay between international collaboration and scientific impact: evidence from the Scopus database. *Cogent Educ*. 2025;12(1):2563708. doi:10.1080/2331186X.2025.2563708

26. Hong QN, Fàbregues S, Bartlett G, et al. The Mixed Methods Appraisal Tool (MMAT) version 2018 for information professionals and researchers. *Educ Inf*. 2018;34(4):285-291. doi:10.3233/EFI-180221

27. McDermott KT, Perry M, Linden W, Croft R, Wolff R, Kleijnen J. The quality of COVID-19 systematic reviews during the coronavirus 2019 pandemic: an exploratory comparison. *Syst Rev*. 2024;13(1):126. doi:10.1186/s13643-024-02552-x

28. Dossett LA, Kaji AH, Cochran A. SRQR and COREQ Reporting Guidelines for Qualitative Studies. *JAMA Surg*. 2021;156(9):875-876. doi:10.1001/jamasurg.2021.0525

Supplementary Material

Supplementary Material 1. Preferred Reporting Items for Systematic reviews and Meta-Analyses extension for Scoping Reviews (PRISMA-ScR) checklist.

Supplementary Material 2. Characteristics of the included studies (n=147)

Supplementary Material 3. Table of excluded studies (n=55)

1

Figure 1

Click here to access/download;Figure;2. Fig1.tif

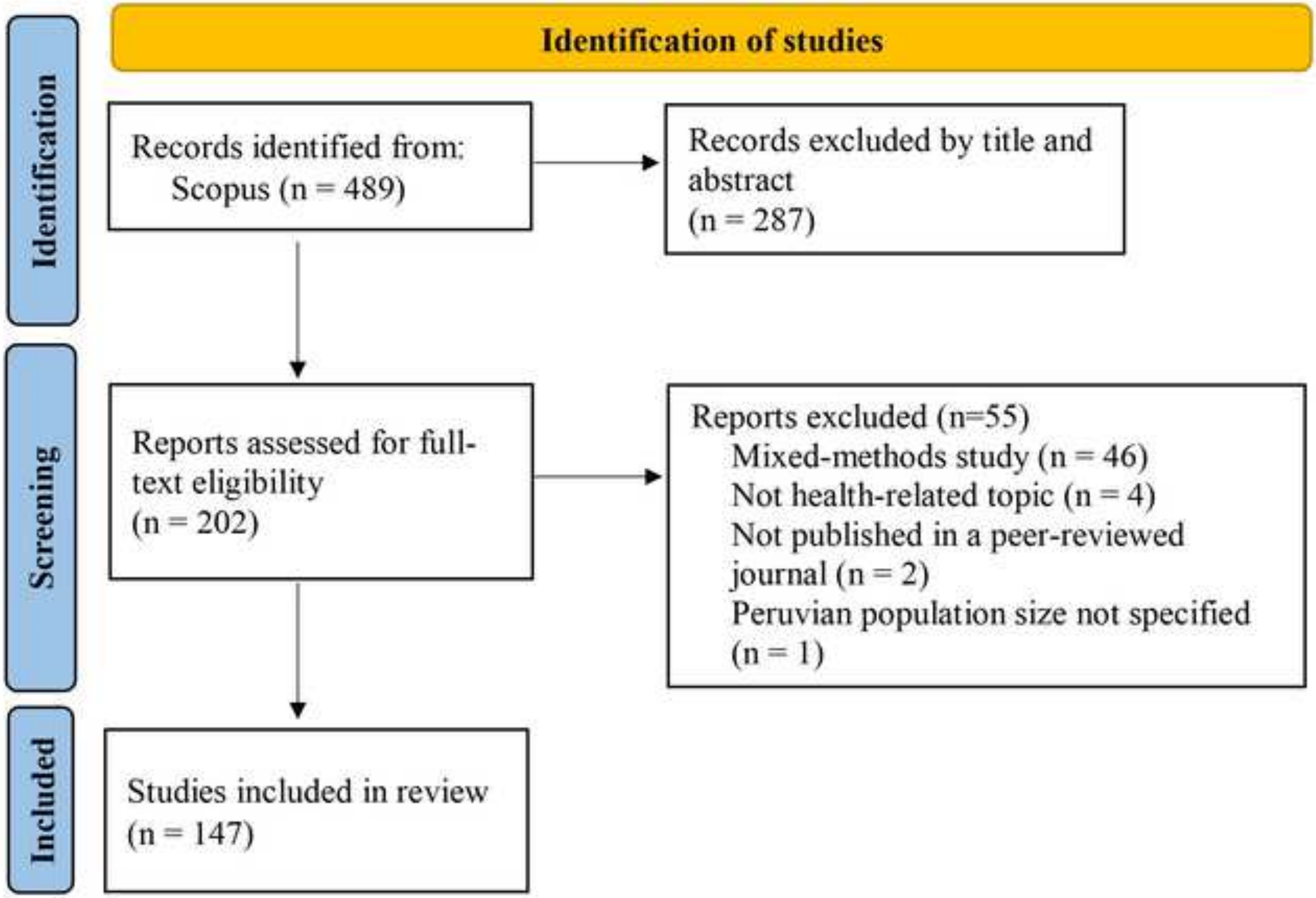

Figure 2

Click here to access/download;Figure;3. Fig2.tif

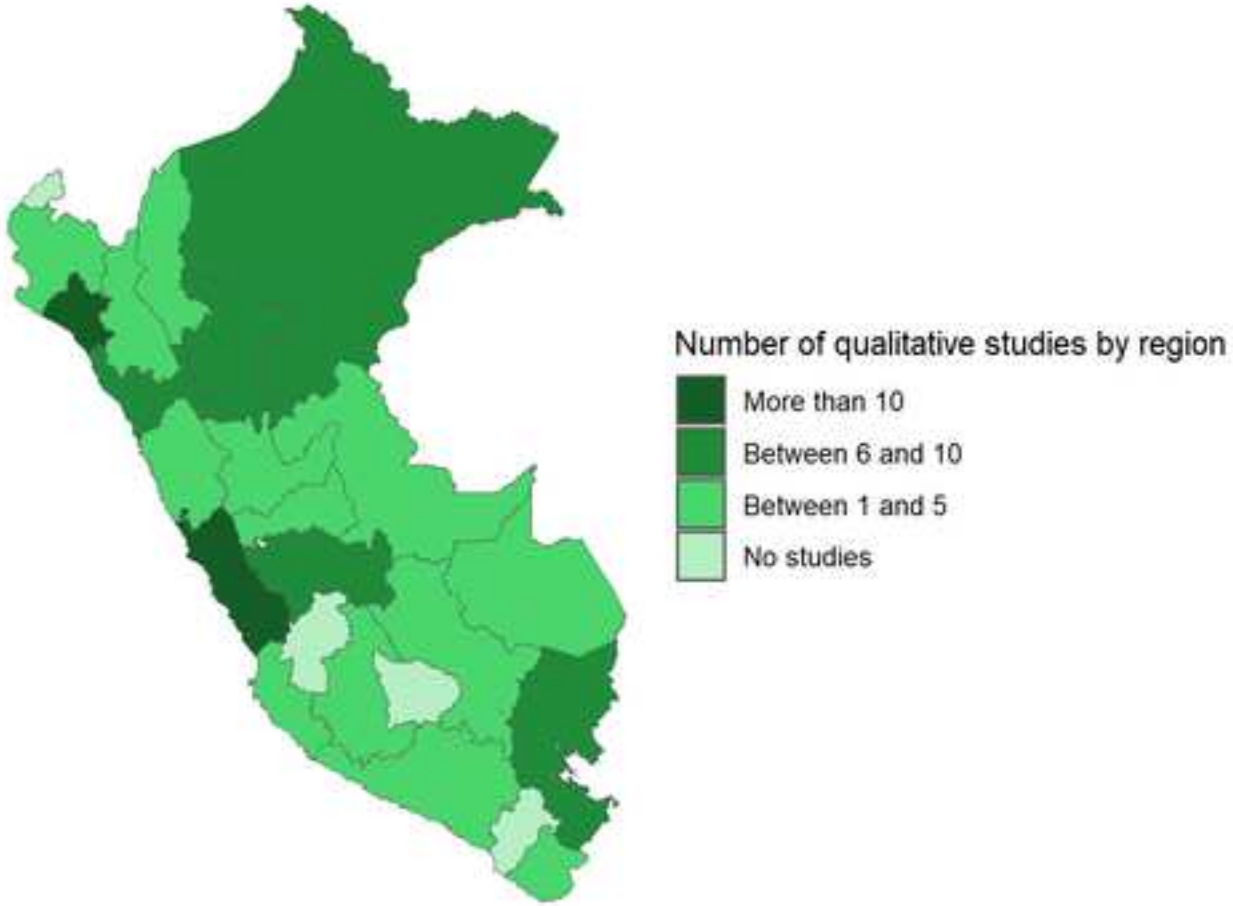

Figure 3

Click here to access/download;Figure;4. Fig3.tif

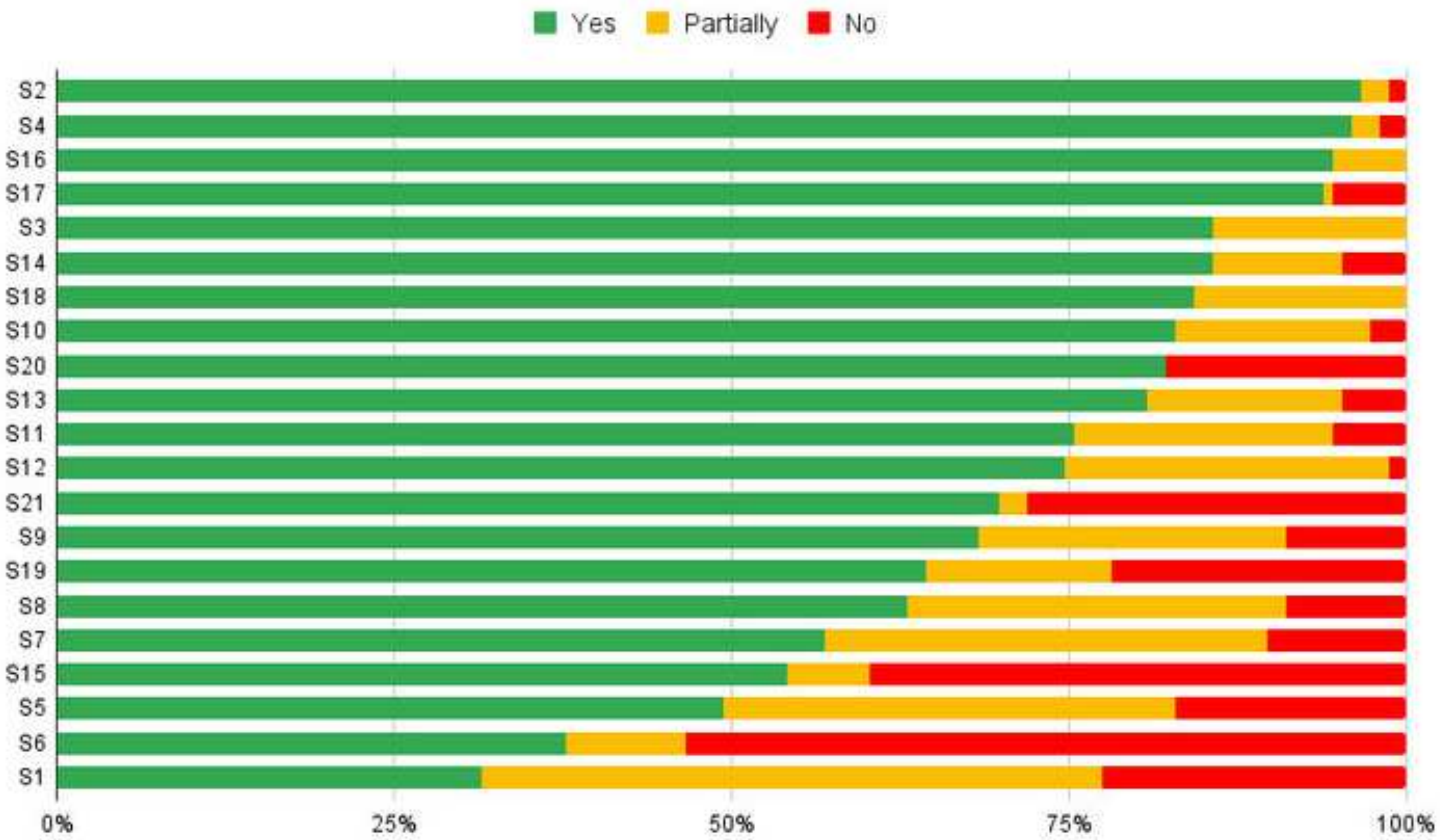

Click here to access/download
Supporting Information
6. Supplementary Material 2.docx

Click here to access/download
Supporting Information
7. Supplementary Material 3.docx

Supporting Information 1

Click here to access/download
Supporting Information
PRISMA-ScR-Fillable-Checklist_10Sept2019.docx
