# Supplementary material for: Reporting quality of qualitative health studies published by Peruvian authors: A scoping review
Source: PLoS One. 2026 Jun 23;21(6):e0351494. doi: 10.1371/journal.pone.0351494 (PMC13289893; doi:10.1371/journal.pone.0351494)
Supplement: Attachment — Submitted filename: PONE-D-26-05648_R1-AF.pdf [file pone.0351494.s007.pdf]

PLOS One

Calidad de la información en estudios cualitativos de salud publicados por autores peruanos: una revisión exploratoria

--Manuscript Draft--

Manuscript Number:	PONE-D-26-05648R1
Article Type:	Research Article
Full Title:	Calidad de la información en estudios cualitativos de salud publicados por autores peruanos: una revisión exploratoria
Short Title:	Quality of Reporting in Peruvian Qualitative Health Studies
Corresponding Author:	Rubí Paredes-Angeles<br>Universidad Cientifica del Sur<br>Lima, PERU
Keywords:	Qualitative research; reporting quality; Standards for Reporting Qualitative Research; scoping review; Peru
Abstract:	La investigación cualitativa ofrece valiosas perspectivas sobre procesos sociales y conductuales complejos en las ciencias de la salud; sin embargo, su contribución depende de la calidad y transparencia de la presentación de informes. Esta revisión exploratoria tuvo como objetivo describir las características metodológicas y la calidad de la presentación de informes de estudios cualitativos publicados por autores peruanos entre 2022 y 2025. Siguiendo las directrices PRISMA-ScR, se realizó una búsqueda sistemática en SCOPUS y se evaluó la calidad de la presentación de informes utilizando la herramienta Estándares para la Presentación de Informes de Investigación Cualitativa (SRQR). Se incluyeron un total de 147 estudios cualitativos. La mayoría se publicaron en inglés (49,0%), tenían una mediana de cinco autores; el 71,4% tenía un primer autor afiliado a una institución peruana, y el 58,5% involucraba exclusivamente a autores peruanos. Los estudios se realizaron principalmente en Lima (50,7%). La aprobación del comité de ética no se informó en el 24,5% de los estudios, y el 40,8% no proporcionó un código de aprobación ética. La adherencia a los ítems del SRQR fue mayor para los resúmenes (95,9%), el propósito del estudio (95,2%) y la presentación de los hallazgos (93,9%), mientras que la notificación de los elementos del título (31,3%), el enfoque cualitativo (49,0%) y la reflexividad del investigador (37,4%) fue limitada. La publicación en español y la autoría principal peruana se asociaron con puntuaciones más bajas en el SRQR, mientras que un mayor número de autores y la colaboración internacional se asociaron con puntuaciones más altas. En general, los estudios cualitativos publicados por autores peruanos mostraron una calidad de notificación variable, con aspectos metodológicos y éticos importantes frecuentemente subestimados, lo que resalta la necesidad de fortalecer las prácticas de notificación, particularmente para los componentes cualitativos centrales.
Order of Authors:	Cristhian Rojas-Miliano
	Andrea Dámaso-Román
	Guillermo Almeida-Huanca
	Yolanda Viguria-Chavez
	Nikol Mayo-Puchoc
	Andy Rick Sánchez-Villena
	Alvaro Taype-Rondan
	Rubí Paredes-Angeles
Opposed Reviewers:	
Response to Reviewers:	Dear editor,<br>Many thanks for your response to the submission of our paper<br><br>We sincerely thank you and the reviewers for their thoughtful and constructive

Powered by Editorial Manager® and ProduXion Manager® from Aries Systems Corporation

comments regarding the submission of our manuscript. We have carefully revised the manuscript in response to all reviewers' feedback and have addressed each suggestion in detail. We trust that the revisions undertaken have enhanced the clarity and overall quality of the manuscript. Our point-by-point responses to each comment are provided below.

Rubí Paredes-Angeles
rubiparedesa@gmail.com
Corresponding author

Review Comments to the Author

Reviewer #1
Comment:
Introduction section, paragraph 4, page 6
The authors mentioned that health research publications have increased in Peru. They cited the source of that information. Although it is okay, they could have spiced up the argument by stating the magnitude of the increase.

Reply: In response, we have specified the magnitude of the increase in health research in Peru. The following sentence was added in Introduction section: "In Peru, scientific research output has increased, with an estimated annual growth rate of 13.6%, with 70.2% corresponds to health sciences [14]"

Comment: The authors have claimed that "to date, no studies have specifically examined qualitative health research or assessed the quality of its reporting." The argument lacks clarity and evidence. Can they provide evidence that their study is of the first kind in Peru?
Study eligibility, paragraph 2, page 7

Reply: We have revised the statement to avoid an overgeneralized claim and improve clarity. The paragraph now reads in Introduction Section: "A previous study examined the scientific production of qualitative research in Peru found that the publication trend has increased since 2013; however, it did not assess the reporting quality of these studies [15]." This correction acknowledges prior evidence, incorporates the relevant reference [15], and recognizes the limitation regarding the absence of reporting quality assessment.

Comment: "Only studies in which at least 25% of participants were based in Peru were considered." Why? Can this statement be supported with the reason for including studies in which at least 25% of participants were based in Peru?

Reply: We added the following sentence in the second paragraph of the "Study eligibility" section: "Only studies in which at least 25% of participants were based in Peru were considered to ensure meaningful representation of the Peruvian population while allowing the inclusion of international studies."

Comment: Conclusion, pages 22 and 23
Conclusion lacks recommendations from the study's findings.

Reply: The conclusion has been revised as follows: "This review identifies key areas for improvement in the reporting of qualitative health studies in the Peruvian population, including techniques to ensure trustworthiness and credibility, methodological approach, researcher reflexivity, title quality, and reporting of ethics approval. Editorial teams could encourage adherence to standardized reporting guidelines and make editorial policies more explicit regarding ethics committee approval and key elements of methodological transparency, thereby supporting improved reporting practices. Future research could investigate whether these challenges persist in other contexts with similar structural and geographical disparities."

Comment: The authors have performed statistical analysis appropriately and

rigorously. They have used simple statistical analyses by reporting prevalence ratios and frequencies. However, they should consider revising the presentation of the frequencies in Table 1. It is more appearing to arrange the frequencies of the variables in a table, either from highest to lowest percentages or vice versa. Table 1 is on pages 13 and 14.

Reply: The order of variables in Table 1 has been revised to present them in descending order according to their percentages.

Comment: The authors did not make the data underlying the findings in their manuscript fully available. However, they state that the data will be available upon reasonable request from the corresponding author.

Reply: We have revised the Data Availability Statement to improve transparency. It now states: "The raw data underlying the findings are available in the S4 Table." Therefore, the complete data can be accessed in this supplementary material.

Comment: The manuscript is presented in an intelligible fashion and written in standard English. However, in the discussion section (main results, page 17), the paragraph is redundant to what has already been presented in the results section. Again, on pages 7 and 8, the Spanish was mentioned twice. Additionally, using the word "to date" (page 6) will be vague if the paper is read in 2 or more years from now. Can authors replace "to date" with other word(s)?

Reply: The "Main Results" paragraph in the Discussion section has been revised to avoid redundancy with the Results section. Specifically, numerical values were removed and replaced with approximate descriptive expressions (e.g., "nearly one quarter" instead of "24.5%") to improve readability and reduce repetition. Regarding the language variable (pages 7–8), the wording was clarified to avoid repetition. It has been revised as follows: "language of the study (English, Spanish, or both English and Spanish)". Finally, the expression "to date" has been removed and replaced with more precise wording, as mentioned before.

Reviewer #2:

Comment: Introduction. 1/ p.6: The study aim is stated as follows: "this study aimed to describe the methodological characteristics and assess the reporting quality of qualitative studies published by Peruvian authors." (p.6). In the eligibility criteria, authors stated: "Only studies in which at least 25% of participants were based in Peru were considered." (p.11).
This is a bit confusing. Authors should specify clearly if it's about qualitative studies published by Peruvian authors or qualitative studies conducted in Peru.

Reply: To clarify this point, we revised the final sentence of the Introduction (study aim) as follows: "this study aimed to describe the methodological characteristics and assess the reporting quality of qualitative studies conducted in populations residing in Peru and involving at least one author affiliated with a Peruvian institution." This revision clarifies that the study includes both research conducted in Peruvian populations and studies involving at least one author with a Peruvian institutional affiliation. This criterion is also consistently specified in the "Study eligibility" section.

Comment: Methods. 1/ p.6: Why focusing especially on 2022-2025 period to get recent studies? Authors have provided an explanation in Discussion. However, are the studies published prior 2022-2025 no more relevant or used to inform health practice and policy?

Reply: Our decision to restrict the analysis to studies published between 2022 and 2025 was not based on the assumption that earlier studies are no longer relevant. Rather, it was a deliberate methodological choice aligned with the objective of this scoping review, which was to describe the current state of reporting practices in qualitative health research conducted by Peruvian authors.

Focusing on recent years allows for a more accurate assessment of contemporary reporting standards, particularly in the context of the increasing dissemination and

Powered by Editorial Manager® and ProduXion Manager® from Aries Systems Corporation

adoption of reporting guidelines such as SRQR. In addition, this timeframe reduces potential distortions associated with atypical publication patterns observed during the COVID-19 pandemic period.

Comment: 2/ p.6: Authors claim to have conducted a systematic search. Yet they only searched Scopus without a justification. Why did they search only one database and how inclusive/comprehensive do they think their review is? Did they consider other sources such as references lists of identified articles?

Reply: The decision to use Scopus as the sole database was based on its broad coverage and its capacity to filter results by authors' institutional affiliation (i.e., affiliation with Peru), which was a key eligibility criterion in our review. To our knowledge, other commonly used databases such as PubMed, SciELO, or LILACS do not allow for such precise and reproducible filtering by affiliation, which limited their suitability for our specific objective. Regarding additional search strategies, we did not perform manual searches of reference lists. Given the scope of this review and the structured search strategy applied, we prioritized a reproducible and database-driven approach.
Therefore, our findings should be interpreted as representative of the qualitative health research indexed in Scopus rather than the entirety of qualitative research conducted in Peru. These limitations have been explicitly acknowledged in the revised manuscript.
Comment: 3/ p.7: How relevant do you think it would be to group studies by health disciplines? Researchers usually work in specific health disciplines and your findings would be more insightful if you provide them with discipline-based evidence.

Reply: Although we initially considered it, many of the included studies addressed interdisciplinary topics or involved overlapping populations and settings, which posed difficulties for a coherent and mutually exclusive classification into distinct health disciplines. Nevertheless, we recognize the potential value of this approach. In our study, we partially addressed this dimension by categorizing studies according to their main population and research setting, which may serve as proxies for disciplinary focus.

Comment: 4/ p.8: Authors used the highest tertile to define "adequate SRQR reporting". Is this threshold a standard in the field or just authors' choice? This should be clarified.

Reply: The use of the highest tertile to define "adequate SRQR reporting" was a methodological decision made by the authors rather than a standardized or universally established threshold in the field.
Currently, there is no cut-off point for categorizing reporting quality based on SRQR scores. In this context, we opted for a data-driven approach by classifying studies according to the distribution of scores within our sample. Defining "adequate reporting" as the highest tertile allowed us to identify studies with comparatively better adherence to SRQR items while maintaining sufficient variability for the analysis of associated factors. This approach has been used in previous methodological studies, where relative thresholds (e.g., tertiles or quartiles) are applied in the absence of validated cut-offs. However, we acknowledge that such categorization is somewhat arbitrary and should be interpreted cautiously.
We have added in Methods: "Given the absence of established or validated cut-off points for SRQR scores, this data-driven approach was used to identify studies with comparatively better reporting quality."

Comment: 5/ p.8-11: This SRQR-based study assessment remains somehow subjective since the appreciation may vary by assessor. I believe that if another research group had to reproduce this review, they may give different scores to each study for each item. Are authors aware of this subjectivity and variability concern? What strategy did they implement to mitigate this limitation?

Reply: We acknowledge that SRQR-based assessments may involve a degree of subjectivity, as judgments can vary across assessors. To mitigate this, we implemented several strategies to enhance consistency and reproducibility. First, our

data extraction matrix included two components for the SRQR score: one for the assigned score and another to document the rationale for that score, including verbatim excerpts from the reviewed articles to justify each rating. Second, each pair of investigators independently extracted study characteristics and SRQR items. They then met to compare each cell and reach consensus on the score assigned to each item, resulting in a final extraction. Discrepancies that could not be resolved were discussed during weekly team meetings until agreement was achieved. Additionally, prior to formal data extraction, the team conducted calibration exercises to ensure a shared understanding of SRQR criteria and consistent application across reviewers.

To clarify this, we have added in Methods section: “The data extraction matrix included two components for the SRQR score: one for the assigned score and another to document the rationale, including verbatim excerpts from the reviewed articles to justify each rating. Each pair of investigators independently extracted study characteristics and SRQR items, and then met to compare each cell and reach consensus, resulting in a final extraction. Discrepancies that could not be resolved were discussed during weekly team meetings until agreement was achieved.”

Additionally, we added the following statement to the limitations section of the discussion: "Fourth, although the SRQR provides a structured framework for evaluating reporting quality, the interpretation of some items may involve a degree of reviewer subjectivity."

Comment: 6/ It is widely recognized that study reporting quality depends on research expertise and vary both by health discipline (dentistry vs. public health) and by authors' affiliation type (clinical vs. research institution affiliation). Since the information on health discipline was available, why did authors not consider it as a descriptive characteristic and subsequently test its association with adequate reporting categories?

Reply: We thank the reviewer for this comment. While we agree that reporting quality may vary across health disciplines, we did not include this variable due to challenges in consistently classifying studies into mutually exclusive categories, particularly for multidisciplinary research. As we mentioned above, this could introduce misclassification and affect the interpretability of the findings.

Comment: Results 1/ Please format all tables according to the journal's guidelines.

Reply: All tables have been revised and reformatted to comply with the journal's guidelines.

Comment: 2/ Figure 1: authors stated that 287 studies were excluded by title and abstract. Can they briefly specify the reasons both in the figure and in the descriptive text?

Reply: The reasons for exclusion at the title and abstract screening stage have now been specified in Figure 1 and clearly described in the corresponding section of the text. Specifically, the following sentence was added to the Results section: “Records were excluded at this stage because they did not meet the eligibility criteria, including studies that were not qualitative, were not related to health sciences, did not involve populations residing in Peru, or corresponded to non-original publications.”

Comment: 3/ Figure 2: mandatory elements of a geographical map, such as direction (North) and gradient (scale), are missing. Please consider adding them.

Reply: Figure 2 has been revised to include the mandatory cartographic elements, including the North direction and scale, in accordance with standard conventions.

Comment: 4/ Adequate reporting is reported in Table 1 but not commented in the text. Please consider adding an in-text comment and a table footnote recalling how it was defined.

Reply: An in-text comment has been added to the Results section, and a footnote has been included in Table 1 to clarify the definition of adequate reporting. Specifically, the

Powered by Editorial Manager® and ProduXion Manager® from Aries Systems Corporation

	following sentence was incorporated into the text: “Adequate SRQR reporting was observed in 32.7% of the included studies.”<br><br>Comment: 5/ p.15: It seems counterintuitive to interpret an association by the reference group as authors did for Table 2 regarding non-Peruvian vs. Peruvian first author’s affiliation (PR = 0.40, 95% CI: 0.26–0.62). If authors wish to keep this interpretation, I suggest them to assign the reference group to Peruvian authors.<br><br>Reply: Thank you for this valuable comment. However, we deliberately selected non-Peruvian affiliation as the reference category to maintain consistency with our analytical framework and to emphasize comparisons with international authorship across both the Results and Discussion sections.<br><br>It is important to highlight that the interpretation of the results remains valid regardless of the reference group chosen, as it reflects the relative difference between categories. In this context, our findings indicate a lower prevalence of adequate reporting among qualitative studies with Peruvian first authors compared to those with non-Peruvian first authors. This interpretation has been applied consistently throughout the manuscript.<br><br>Comment: Discussion 1/ Considering you research rationale; how do authors interpret and explain a study with one Peruvian author (for instance at sixth position) and many foreigner co-authors? Is it treated as a Peru-affiliated study at the same level as the one exclusively authored by Peru-based researchers?<br><br>Reply: We anticipated this potential heterogeneity prior to data extraction; therefore, we included the variables “country of the first author’s primary affiliation” and “international collaboration”. These variables were incorporated into the analyses to better capture and examine the influence of different authorship and collaboration patterns.<br><br>Comment: 2/ p.17: Please, authors should remove proportions from the Main Results recall in the Discussion section. Instead, they could use descriptive terms such as half of studies, three-quarter, one-third, etc. which are more appealing as summary.<br><br>Reply: We have revised the Main Results recall in the Discussion section by replacing proportions with descriptive terms (e.g., most, almost half), as recommended, to improve readability and narrative flow.<br><br>Comment 3/ p.19-20: Authors compare their findings with the existing literature. They mainly focused on similar findings, while less is said about divergence between their results and the existing evidence. Moreover, less is said about the studies of comparison besides thematic areas. They should consider improving this.<br><br>Reply: We appreciate this insightful comment. We agree that a balanced discussion should address both convergent and divergent findings when contextualizing results within the existing literature. Although the number of available studies is limited, the existing evidence on reporting practices in qualitative research is largely consistent with our findings, indicating that our interpretation does not rely on a one-sided perspective.<br><br>Regarding thematic areas, we would like to emphasize that this aspect has been previously specified and discussed in the manuscript: "However, unlike those studies, which focused on specific populations or thematic areas, our analysis encompasses the qualitative scientific output of an entire region. This convergence suggests that there is a persistent tendency to report items that are commonly required in scientific manuscripts, irrespective of study design (e.g., abstract key aspects, objective, main findings), reflecting adherence to general reporting conventions rather than specific compliance with qualitative reporting guidelines. By contrast, several items that are fundamental to the rigor and transparency of qualitative research were among the least frequently reported."<br><br>Comment: 4/ p.20: Authors should consider revising the paragraph #1 of the subsection “Factors associated with…” First, delete “…which could partially explain the observed association” because it is repetitive with the second sentence starting: “This may be explained by the tendency…”. Second, another explanation to consider may be

Powered by Editorial Manager® and ProduXion Manager® from Aries Systems Corporation

	the fact that English studies are often co-authored by international researchers, requiring English as the collaborative language and bringing additional level of rigor and expertise, as acknowledged in the subsequent paragraph.<br><br>Reply: We have removed the phrase "…which could partially explain the observed association" as recommended, to avoid redundancy. In its place, we have incorporated the following sentence: "Additionally, studies published in English may more frequently involve international collaborations, which can contribute additional methodological expertise and promote stricter adherence to reporting standards." Furthermore, in the subsequent paragraph, we have reinforced this interpretation by adding: "These findings may reflect the potential contribution of international collaboration to improving reporting standards in qualitative research involving Peruvian populations."<br><br>Comment 5/ p.21-22: Two major limitations need to be considered. First, there is a rater subjectivity in using SRQR checklist that should not be dismissed, yet not discussed by authors. This can be observed in the Strengths paragraph where authors praise their "flexibility" in rating studies, which was even not mentioned in Methods. Second, regression analysis estimated crude associations that should be explicitly acknowledged in the discussion alongside their implications for both these findings and future research.<br><br>Reply: We agree that both aspects raised should be explicitly acknowledged as limitations. Accordingly, we have added the following statement to address potential reviewer subjectivity in the use of the SRQR checklist: "Fourth, although the SRQR provides a structured framework for evaluating reporting quality, the interpretation of some items may involve a degree of reviewer subjectivity." In addition, we have incorporated the following sentence to clarify the nature of the regression analysis: "Finally, the regression analysis estimated crude associations; therefore, the observed relationships should be interpreted cautiously and should not be interpreted as evidence of causal relationships."<br><br>Comment: Conclusion 1/ p.22: The conclusion reads more like results recall than overall summary and practical recommendations for qualitative health research stakeholders. Authors should consider complying with the PRISMA-ScR recommendation relative to conclusion: "Provide a general interpretation of the results with respect to the review questions and objectives, as well as potential implications and/or next steps."<br><br>Reply: We have fully revised the Conclusions paragraph in accordance with the PRISMA-ScR recommendations, incorporating a general interpretation of the findings along with their practical implications and potential next steps. The revised paragraph reads as follows: "This review identifies key areas for improvement in the reporting of qualitative health studies in the Peruvian population, including techniques to ensure trustworthiness and credibility, methodological approach, researcher reflexivity, title quality, and reporting of ethics approval. Editorial teams could encourage adherence to standardized reporting guidelines and make editorial policies more explicit regarding ethics committee approval and key elements of methodological transparency, thereby supporting improved reporting practices. Future research could investigate whether these challenges persist in other contexts with similar structural and geographical disparities."<br><br>* MINOR COMMENTS<br><br>Comment: 1/ Please, revise the referencing format throughout the text according to the journal's guidelines.<br><br>Reply: The referencing format has been revised throughout the manuscript to ensure full compliance with the journal's guidelines.<br><br>Comment: 2/ p.6: I suggest to reformulate concisely the following text: From "A detailed study protocol was developed prior to study initiation and made publicly available. The protocol of this review is available at https://osf.io/jes6v/files/3rn52." to "A detailed study protocol was developed prior to study initiation and made publicly available (https://osf.io/jes6v/files/3rn52)"

Reply: The text has been revised following your recommendation to improve conciseness and clarity.

Comment: 3/ p.7: Please, provide an internet link to Rayyan software to align with proper software referencing (city, company, website). Same for Stata software and revise as follows: "…Stata software, version 19.0." (p.11)

Reply: The manuscript has been revised accordingly to ensure proper software referencing. Institutional details and website links have been added for both tools. Specifically, we included: "Rayyan software (Qatar Computing Research Institute, Doha, Qatar; https://www.rayyan.ai/)" and "Stata software, version 19.0 (StataCorp, College Station, TX, USA; . https://www.stata.com/)."

Comment: 4/ p.7: Please revise: "…with the collaboration of other authors." or "…with the collaboration of another author."

Reply: The text has been corrected as indicated.

Comment: 5/ A lot of paragraphs are too short, with only 1 to 3 sentences within a subsection. Please, revise to make paragraphs more compact.

Reply: The manuscript has been revised by merging some paragraphs to improve cohesion and achieve a more compact presentation of the content.

Comment: 6/ p.11: Please, use appropriate terminology: replace "numeric variables" by "quantitative variables"

Reply: The text has been corrected as indicated.

Comment 7/ p.11: Please, add statistical threshold: "…crude prevalence ratios (PR) with 95% confidence intervals (CI) were estimated…"

Reply: The text has been corrected as indicated.

Comment 8/ Please, reformulate Table 2 title as follows: "Factors associated with adequate…"

Reply: The text has been corrected as indicated.

Comment 9/ I am just curious to know how long it took to check 147 studies against a 21-item checklist.

Reply: Thank you for your interest. The evaluation of the studies across its different stages (peer review of titles and abstracts, full-text assessment, and data extraction) was carried out over approximately eight weeks. We considered this timeframe appropriate to ensure a careful and thorough evaluation of each study, given the number of authors involved.

Journal Requirements:

Comment 1: About PLOS ONE's style requirements

Reply: We have carefully reviewed the manuscript and ensured that it now fully complies with PLOS ONE's style requirements, including those related to file naming. The corresponding corrections have been implemented following the provided formatting templates.

Comment 2: About funding section

Reply: We have removed all funding-related information from the manuscript to ensure that such details appear exclusively in the Funding Statement section, in accordance with PLOS ONE guidelines. We would like to update the Funding Statement as follows: "This study was self-funded by the authors. YVC's time was funded by the

	Neuropsychiatry and Behavioral Disorders in First Nations Multidisciplinary Training Program (NEUFIN) grant, NIMH D43MH108169." Please proceed with updating the online submission form accordingly.<br><br>Comment 3: About ethics statement<br>Reply: The ethics statement has been removed from all sections other than the Methods and is now presented exclusively there, in accordance with the journal's guidelines.<br><br>Comment 4: About Figure 2<br><br>Reply: We would like to clarify that Figure 2 was entirely generated by the authors using R (packages "sf" and "mapsPERU"). The map is based on vector-based administrative boundary data of Peru obtained from publicly available sources. No proprietary basemaps (such as Google Maps, Google Earth, or similar services) were used at any stage of the figure creation process. Therefore, the figure does not contain copyrighted map tiles or satellite imagery and is fully compatible with the Creative Commons CC BY 4.0 license required by PLOS ONE.<br><br>We have included in Methods: "Spatial data processing and visualization were performed in R (version 4.2.2; R Foundation for Statistical Computing, Vienna, Austria). Administrative boundary data for Peru were obtained from the "mapsPERU" package (version 2.0.0), which provides publicly available vector-based geospatial data. Spatial data were managed using the "sf" package (version 1.0.16). Data manipulation was conducted using "dplyr" package (version 1.1.4) and "ggplot2" package (version 3.5.1). No proprietary basemaps were used."<br><br>Comment 5: Regarding new references<br><br>Reply: In response to the reviewers' recommendation, we have added reference 15. This reference was included because, although previous Peruvian qualitative studies exist, they had not been assessed for reporting quality. Its inclusion provides important context, highlighting the presence of prior literature while underscoring the gap in evaluating reporting standards, as emphasized in the manuscript.<br><br>Comment 6: Regarding Reference List<br><br>Reply: We have reviewed the reference list and confirm that no changes were necessary, as our manuscript does not cite any retracted studies.
**Additional Information:**	
**Question**	**Response**
**Financial Disclosure**<br><br>Enter a financial disclosure statement that describes the sources of funding for the work included in this submission. Review the submission guidelines for detailed requirements. View published research articles from *PLOS ONE* for specific examples.<br><br>This statement is required for submission and **will appear in the published article** if the submission is accepted. Please make sure it is accurate.	Yes

**Funded studies**<br>Enter a statement with the following details:<br>• Initials of the authors who received each award<br>• Grant numbers awarded to each author<br>• The full name of each funder<br>• URL of each funder website<br>• Did the sponsors or funders play any role in the study design, data collection and analysis, decision to publish, or preparation of the manuscript?<br><br>Did you receive funding for this work?	
Please add funding details.<br>as follow-up to "**Financial Disclosure**<br><br>Enter a financial disclosure statement that describes the sources of funding for the work included in this submission. Review the submission guidelines for detailed requirements. View published research articles from *PLOS ONE* for specific examples.<br><br>This statement is required for submission and **will appear in the published article** if the submission is accepted. Please make sure it is accurate.<br><br>**Funded studies**<br>Enter a statement with the following details:<br>• Initials of the authors who received each award<br>• Grant numbers awarded to each author<br>• The full name of each funder<br>• URL of each funder website<br>• Did the sponsors or funders play any role in the study design, data collection and analysis, decision to publish, or preparation of the manuscript?<br><br>Did you receive funding for this work?"	This study was self-funded by the authors. YVC's time was funded by the Neuropsychiatry and Behavioral Disorders in First Nations Multidisciplinary Training Program (NEUFIN) grant, NIMH D43MH108169.
Please select the country of your main research funder (please select carefully as in some cases this is used in fee calculation).	

as follow-up to "**Financial Disclosure**<br><br>Enter a financial disclosure statement that describes the sources of funding for the work included in this submission. Review the submission guidelines for detailed requirements. View published research articles from *PLOS ONE* for specific examples.<br><br>This statement is required for submission and **will appear in the published article** if the submission is accepted. Please make sure it is accurate.<br><br>**Funded studies**<br>Enter a statement with the following details:<br>• Initials of the authors who received each award<br>• Grant numbers awarded to each author<br>• The full name of each funder<br>• URL of each funder website<br>• Did the sponsors or funders play any role in the study design, data collection and analysis, decision to publish, or preparation of the manuscript?<br><br>Did you receive funding for this work?"	
**Competing Interests**<br><br>Use the instructions below to enter a competing interest statement for this submission. On behalf of all authors, disclose any competing interests that could be perceived to bias this work—acknowledging all financial support and any other relevant financial or non-financial competing interests.<br><br>This statement is required for submission and **will appear in the published article** if the submission is accepted. Please make sure it is accurate and that any funding sources listed in your Funding Information later in the submission form are also declared in your Financial Disclosure statement.	The authors have declared that no competing interests exist.

View published research articles from *PLOS ONE* for specific examples.<br><br>**NO authors have competing interests**<br><br>Enter: *The authors have declared that no competing interests exist.*<br><br>**Authors with competing interests**<br><br>Enter competing interest details beginning with this statement:<br><br>*I have read the journal's policy and the authors of this manuscript have the following competing interests: [insert competing interests here]*<br><br>* typeset	
**Data Availability**<br><br>Authors are required to make all data underlying the findings described fully available, without restriction, and from the time of publication. PLOS allows rare exceptions to address legal and ethical concerns. See the PLOS Data Policy and FAQ for detailed information.<br><br>A Data Availability Statement describing where the data can be found is required at submission. Your answers to this question constitute the Data Availability Statement and **will be published in the article**, if accepted.<br><br>**Important:** Stating 'data available on request from the author' is not sufficient. If your data are only available upon request, select 'No' for the first question and explain your exceptional situation in the text box.<br><br>Do the authors confirm that all data underlying the findings described in their manuscript are fully available without restriction?	Yes - all data are fully available without restriction
Additional data availability information:	

**Ethics Statement**<br><br>Enter an ethics statement for this submission. This statement is required if the study involved:<br><br>• Human participants<br>• Human specimens or tissue<br>• Vertebrate animals or cephalopods<br>• Vertebrate embryos or tissues<br>• Field research<br><br>Write "N/A" if the submission does not require an ethics statement.<br><br>General guidance is provided below. Consult the submission guidelines for detailed instructions. **Make sure that all information entered here is included in the Methods section of the manuscript.**	N/A

Powered by Editorial Manager® and ProduXion Manager® from Aries Systems Corporation

**Format for specific study types**<br><br>**Human Subject Research (involving human participants and/or tissue)**<br>• Give the name of the institutional review board or ethics committee that approved the study<br>• Include the approval number and/or a statement indicating approval of this research<br>• Indicate the form of consent obtained (written/oral) or the reason that consent was not obtained (e.g. the data were analyzed anonymously)<br><br>**Animal Research (involving vertebrate animals, embryos or tissues)**<br>• Provide the name of the Institutional Animal Care and Use Committee (IACUC) or other relevant ethics board that reviewed the study protocol, and indicate whether they approved this research or granted a formal waiver of ethical approval<br>• Include an approval number if one was obtained<br>• If the study involved *non-human primates*, add *additional details* about animal welfare and steps taken to ameliorate suffering<br>• If anesthesia, euthanasia, or any kind of animal sacrifice is part of the study, include briefly which substances and/or methods were applied<br><br>**Field Research**<br><br>Include the following details if this study involves the collection of plant, animal, or other materials from a natural setting:<br>• Field permit number<br>• Name of the institution or relevant body that granted permission	
Data Availability<br><br>Before publication, Authors are required to make fully available and without restriction all data underlying their findings. Sharing the data underlying your findings increases the visibility, credibility, and impact of your work, while supporting transparency and future scientific discovery. Please see our PLOS Data Policy page for detailed information on this policy.</p><br>A Data Availability	All relevant data are within the manuscript and its Supporting Information files.

Statement, detailing where the data can be accessed, is required at first submission. Insert your Data Availability Statement in the box below.</p><br><br>Please see the data reporting of our submission guidelines for instructions on what you need to include in your Data Availability Statement. Examples of acceptable Data Availability Statements are included here for reference:</p><br> <br> - The minimal data set [and accompanying code, where generated] is available at [repository name] via [DOI hyperlink] <br> - No data was generated by this study. The following existing data sources were used: [dataset name] from [dataset location] available via [web address or DOI hyperlink]. <br> - For Study Protocols: No datasets were generated or analysed during the current study. All relevant data from this study will be made available upon study completion. <br> <br><br><i>PLOS allows rare exemptions to address legal and ethical concerns. If you have legal or ethical restrictions, please use the box below to detail these in full sentences for the Journal team to consider.</i></p><br><br>This statement will be typeset if the manuscript is accepted for publication.</p>	

COVER LETTER

Editor-in-Chief
Plos One

Dear Editor,

We are pleased to submit our manuscript entitled: **“Reporting quality of qualitative health studies published by Peruvian authors: a scoping review”** for consideration for publication in Plos One.

This manuscript is a scoping review that evaluates the reporting quality of qualitative health research conducted in the Peruvian population, using the Standards for Reporting Qualitative Research (SRQR) as an assessment framework. While prior studies have examined reporting quality in quantitative or mixed-methods research in Latin America, evidence focusing specifically on qualitative health research and its reporting practices remains scarce. Our study addresses this gap by providing a structured and comprehensive overview of current reporting practices among Peruvian qualitative studies.

Our findings indicate that although general methodological components are frequently reported, elements that are specific and essential to qualitative research—such as researcher reflexivity, analytic processes, and contextual positioning—are often underreported. Additionally, a substantial proportion of studies did not report approval by a research ethics committee. We further explored factors associated with reporting quality, including publication language, first author affiliation, number of authors, and international collaboration, thereby contributing novel insights into structural and contextual influences on reporting practices.

We believe that these findings contribute meaningfully to the existing literature by identifying recurring deficiencies in qualitative research reporting and offering evidence that may inform researchers, editors, and reviewers. Although the study focuses on Peru, the challenges identified may reflect similar issues in countries with comparable research environments.

We confirm that this manuscript is original, is not under consideration by any other journal, and that all authors have approved its submission. There has been no prior interaction with PLOS or this journal regarding this manuscript.

Additionally, at this stage of revisions, we would like to update our funding statement as follows:

"This study was self-funded by the authors. YVC's time was funded by the Neuropsychiatry and Behavioral Disorders in First Nations Multidisciplinary Training Program (NEUFIN) grant, NIMH D43MH108169."

We also request that the order of the last two authors be reversed, as presented in the revised manuscript, with Álvaro Taype-Rondan as the penultimate author and Rubí Paredes-Angeles as the last author.

Thank you for your time and consideration. We appreciate the opportunity to submit our work and look forward to your response.

Sincerely,

Rubí Paredes-Angeles
Corresponding author
Universidad Científica del Sur, Lima, Peru

Manuscrito Click here to access/download;Manuscript;Manuscript.docx

Reporting quality of qualitative health studies published by Peruvian authors: a scoping review

Cristhian Rojas-Miliano[1,2], Andrea Dámaso-Román[3], Guillermo Almeida-Huanca[4], Nikol Mayo-Puchoc[5], Yolanda Viguria-Chavez[6], Andy Rick Sánchez-Villena[7], Alvaro Taype-Rondan[8,9], Rubí Paredes-Angeles[10*]

[1]Universidad Nacional del Centro del Perú, Junín, Perú

[2]Centro de Salud Mental Comunitario Pangoa, Junín, Perú

[3]Universidad Nacional Mayor de San Marcos, Facultad de Psicología, Lima, Perú

[4]Grupo de Investigación en Psicología Social y Desarrollo Humano, Facultad de Psicología,Universidad Nacional Mayor de San Marcos, Lima, Perú

[5]Instituto Peruano de Orientación Psicológica, Lima, Perú

[6]Universidad Peruana Cayetano Heredia, School of Public Health, Lima, Perú

[7]Universidad Señor de Sipán, Vicerrectorado de Investigación, Chiclayo, Perú

[8]Unidad de Investigación para la Generación y Síntesis de Evidencias en Salud,Vicerrectorado de Investigación, Universidad San Ignacio de Loyola, Lima, Perú

[9]EviSalud – Evidencias en Salud, Lima, Perú

[10]Universidad Científica del Sur, Lima, Perú

*Corresponding author

Rubi Paredes-Angeles

E-mail: rubiparedesa@gmail.com (PAR)

Abstract

Qualitative research offers valuable insights into complex social and behavioral processes in the health sciences; however, its contribution depends on the quality and transparency of reporting. This scoping review aimed to describe the methodological characteristics and reporting quality of qualitative studies published by Peruvian authors between 2022 and 2025. Following PRISMA-ScR guidelines, a systematic search was conducted in SCOPUS, and reporting quality was assessed using the Standards for Reporting Qualitative Research (SRQR) tool. A total of 147 qualitative studies were included. Most were published in English (49.0%), had a median of five authors; 71.4% had a first author affiliated with a Peruvian institution, and 58.5% involved exclusively Peruvian authors. Studies were mainly conducted in Lima (50.7%). Ethics committee approval was not reported in 24.5% of studies, and 40.8% did not provide an ethics approval code. Adherence to SRQR items was highest for abstracts (95.9%), study purpose (95.2%), and presentation of findings (93.9%), whereas reporting of title elements (31.3%), qualitative approach (49.0%), and researcher reflexivity (37.4%) was limited. Publication in Spanish and Peruvian first authorship were associated with lower SRQR scores, whereas a higher number of authors and international collaboration were associated with higher scores. Overall, qualitative studies published by Peruvian authors showed variable reporting quality, with important methodological and ethical aspects frequently underreported, highlighting the need to strengthen reporting practices, particularly for core qualitative components.

Key words: Qualitative Research; Reporting quality; Standards for Reporting Qualitative Research; Scoping review; Peru.

Introduction

Qualitative research provides a deeper understanding of complex social and behavioral processes in health sciences [1]. Unlike the quantitative methods, qualitative studies seek to explore, narrate and explain experiences, perceptions, and contexts, offering a transformative perspective that enriches evidence-based decision-making [2,3]. Thus, qualitative research enhances the contextual understanding necessary for developing grounded health interventions [1,2].

However, the usefulness of qualitative findings can be affected by the quality of reporting [4]. Although the inherent heterogeneity of qualitative research reporting reflects its richness and diversity, this variability can also impede systematic evaluation of quality and comparison across studies [5]. Clear, complete, and transparent reporting enables readers to better assess the credibility, transferability, and methodological rigor of qualitative studies.

Some international guidelines have been developed to standardize reporting in qualitative research, including the Standards for Reporting Qualitative Research (SRQR) [6], the Consolidated Criteria for Reporting Qualitative Research (COREQ) [7], and the Enhancing Transparency in Reporting the synthesis of Qualitative research (ENTREQ) [8]. Unlike COREQ, which is tailored for interviews and focus groups, and ENTREQ, which is focused on qualitative synthesis studies, SRQR provides a comprehensive framework that authors can use across a wide range of qualitative methodologies [6,9]. Thus, previous research has employed the SRQR to evaluate reporting quality in qualitative studies conducted in diverse health domains, including urology [10], pediatrics [11], infectious diseases [12], and breast cancer [13].

In Peru, scientific research output has increased, with an estimated annual growth rate of 13.6%, with 70.2% corresponds to health sciences [14]. A previous study examined the scientific production of qualitative research in Peru found that the publication trend has increased since 2013; however, it did not assess the reporting quality of these studies [15]. The absence of such information limits understanding of the transparency and reporting standards within this research field in Peru. Assessing these aspects may help identify strengths, weaknesses, and opportunities for improvement among researchers, reviewers, and editors of scientific journals [16]. Therefore, this study aimed to describe the methodological characteristics and assess the reporting quality of qualitative studies conducted in populations residing in Peru and involving at least one author affiliated with a Peruvian institution.

Methods

This scoping review was carried out and reported following the guidelines of the Preferred Reporting Items for Systematic Reviews and Meta-Analyses Extension for Scoping Reviews (PRISMA-ScR) [17]. The completed PRISMA-ScR checklist is provided in S1 Table . A detailed study protocol was developed prior to study initiation and made publicly available (https://osf.io/jes6v/files/3rn52).

Search strategy

A systematic search was performed in Scopus on October 15, 2025, using the following search strategy to identify qualitative health studies authored by researchers affiliated with institutions in Peru and published between 2022 and 2025: AFFILCOUNTRY (peru) AND

TITLE-ABS (qualitative) AND ((SUBJAREA (MEDI OR NURS OR DENT OR HEAL) OR SUBJAREA (PSYC))) AND PUBYEAR>2021 AND PUBYEAR < 2026

Study eligibility

We included original articles authored by researchers affiliated with institutions in Peru, that were published in scientific journals between 2022 and 2025 and presented findings from qualitative studies. This timeframe was chosen to reflect the current state of the field. Only studies in which at least 25% of participants were based in Peru were considered to ensure meaningful representation of the Peruvian population while allowing the inclusion of international studies. Publications related to health disciplines, including medicine, nursing, dentistry, psychology, and other health professions, were included. Mixed-methods studies were excluded due to the need for specific assessment tools. No language restrictions were applied.

Studies selection

Records were uploaded to the "Rayyan" software (Qatar Computing Research Institute, Doha, Qatar; https://www.rayyan.ai/). Six authors in pairs independently screened the records applying the selection criteria. Disagreements between the reviewers were resolved through discussion among the reviewers or with the collaboration of other authors.

Data extraction

To ensure consistency, a pilot data extraction was conducted prior to the main process. Subsequently, six authors worked in pairs to independently extract data from each study, recording all information in a predesigned Excel spreadsheet. The research team consisted

of professionals in Medicine and Psychology with experience in conducting health research.

For each included study, we collected information on its general and methodological characteristics, including: language of the study (English, Spanish, or both English and Spanish), whether the study was published in a Peruvian journal (yes or no), number of authors (divided in tertiles), country of the first author's institutional affiliation, international collaboration, defined as the inclusion of at least one co-author with an institutional affiliation in a country other than Peru (yes or no), study population, sample size, study setting (community settings, health facilities or educational centers), Peruvian region where the study was conducted, multicountry study, defined as a study that included research sites in countries other than Peru (only Peru or Peru and others countries), ethics committee approval (whether the article reported approval from at least one ethics committee), and ethics approval code reported (whether the article, supplementary material, or appendix included the ethics approval code).

Additionally, we assessed the quality of reporting using the SRQR checklist. The data extraction matrix included two components for the SRQR score: one for the assigned score and another to document the rationale, including verbatim excerpts from the reviewed articles to justify each rating. Each pair of investigators independently extracted study characteristics and SRQR items, and then met to compare each cell and reach consensus, resulting in a final extraction. Discrepancies that could not be resolved were discussed during weekly team meetings until agreement was achieved.

Quality of reporting

We used the SRQR 21-item checklist [6], which provides guidance on transparency and completeness in qualitative research reporting. Each of the 21 items was assessed to

determine whether it was 'reported' (score = 2 points), 'partially reported' (score = 1 point), or 'not reported' (score = 0 points).

The total SRQR score ranged from 0 to 42 points. An item was considered 'reported' when the authors presented the required information in accordance with the SRQR guidelines, 'partially reported' when the authors provided some, but not all, of the required information, and 'not reported' when the authors failed to provide any of the required information according to the SRQR reporting standard [9]. Also, we created the variable “adequate SRQR reporting,” defined as belonging to the highest tertile (≥ 37 points) of the SRQR score. Given the absence of established or validated cut-off points for SRQR scores, this data-driven approach was used to identify studies with comparatively better reporting quality.

This SRQR checklist assessed the following items:

S1. Title. Concise description of the nature and topic of the study. Identifying the study as qualitative/mixed or indicating the approach (e.g., ethnography, grounded theory) or data collection methods (e.g., interview, focus group) is recommended.

S2. Abstract. Summary of key elements of the study using the abstract format of the intended publication; typically includes background, purpose, methods, results, and conclusions.

S3. Introduction. Description and significance of the problem/phenomenon studied; review of relevant theory and empirical work problem statement.

S4. Intro[illegible]tion. Purpose of the study and specific objectives or questions.

S5. Methods. Qualitative approach (e.g., ethnography, grounded theory, case study, phenomenology, narrative research) and guiding theory if appropriate; identifying the

research paradigm (e.g., postpositivist, constructivist/ interpretivist) is also recommended; rationale.

S6. Methods. Researchers' characteristics that may influence the research, including personal attributes, qualifications/experience, relationship with participants, assumptions, and/or presuppositions; potential or actual interaction between researchers' characteristics and the research questions, approach, methods, results, and/or transferability.

S7. Methods. Context. Setting/site and salient contextual factors; rationale.

S8. Methods. Sampling strategy. How and why research participants, documents, or events were selected; criteria for deciding when no further sampling was necessary (e.g., sampling saturation); rationale

S9. Methods. Documentation of approval by an appropriate ethics review board and participant consent, or explanation for lack thereof; other confidentiality and data security issues

S10. Methods. Types of data collected; details of data collection procedures including (as appropriate) start and stop dates of data collection and analysis, iterative process, triangulation of sources/methods, and modification of procedures in response to evolving study findings; rationale

S11. Methods. Description of instruments (e.g., interview guides, questionnaires) and devices (e.g., audio recorders) used for data collection; if/how the instrument(s) changed over the course of the study

S12. Methods (or results). Number and relevant characteristics of participants, documents, or events included in the study; level of participation (could be reported in results)

S13. Methods. Methods for processing data prior to and during analysis, including transcription, data entry, data management and security, verification of data integrity, data coding, and anonymization/deidentification of excerpts"

S14. Methods. Process by which inferences, themes, etc., were identified and developed, including the researchers involved in data analysis; usually references a specific paradigm or approach; rationale

S15. Methods. Techniques to enhance trustworthiness and credibility of data analysis (e.g., member checking, audit trail, triangulation); rationale

S16. Results. Main findings (e.g., interpretations, inferences, and themes); might include development of a theory or model, or integration with prior research or theory

S17. Results. Evidence (e.g., quotes, field notes, text excerpts, photographs) to substantiate analytic findings

S18. Discussion. Short summary of main findings; explanation of how findings and conclusions connect to, support, elaborate on, or challenge conclusions of earlier schola[illegible]p; discussion of scope of application/generalizability; identification of unique contribution(s) to scholarship in a discipline or field

S19. Discussion. Trustworthiness and limitations of findings

S20. Conflicts of interest. Potential sources of influence or perceived influence on study conduct and conclusions; how these were managed

S21. Funding. Sources of funding and other support; role of funders in data collection, interpretation, and reporting.

Data analysis

Data were entered into Microsoft Excel, and all statistical analyses were performed using Stata software, version 19.0 (StataCorp, College Station, TX, USA; https://www.stata.com/). Descriptive analyses included absolute and relative frequencies for categorical variables and medians with interquartile ranges for quantitative variables. Additionally, an exploratory analysis of factors associated with adequate SRQR reporting was conducted. For this purpose, crude prevalence ratios (PR) with 95% confidence intervals (CI) were estimated using Poisson regression with robust variance.

Spatial data processing and visualization were performed in R (version 4.2.2; R Foundation for Statistical Computing, Vienna, Austria). Administrative boundary data for Peru were obtained from the "mapsPERU" package (version 2.0.0), which provides publicly available vector-based geospatial data. Spatial data were managed using the "sf" package (version 1.0.16). Data manipulation was conducted using "dplyr" package (version 1.1.4) and "ggplot2" package (version 3.5.1). No proprietary basemaps were used.

Ethical statement:

Ethics committee approval was not required because this study involved only a review of previously published literature and did not include human participants.

Results

The search yielded 489 records, of which 202 were selected for full-text assessment after title and abstract screening. Records were excluded at this stage because they did not meet the eligibility criteria, including studies that were not qualitative, were not related to health sciences, did not involve populations residing in Peru, or corresponded to non-original publications. Of these, 55 reports were excluded after full-text assessment for the following reasons: mixed-methods design (n = 46), non–health-related topic (n = 4), publication

outside a peer-reviewed journal (n = 2), unspecified Peruvian population size (n = 1), inclusion of less than 25% Peruvian participants (n = 1), and systematic review design (n = 1). Consequently, a total of 147 studies were included in the final analysis (Fig 1). The characteristics of the included studies can be found in S2 Table , while the list of excluded reports is presented in S3 Table .

Figure 1. Flowchart of the article selection process.

Across the included studies, most were published in English (49.0%) and were not published in a Peruvian journal (88.4%). The median number of authors was five, and 37.4% were studies with four to seven authors. Regarding affiliation, the first author most frequently had their primary institutional affiliation in Peru (71.4%), and most studies involved only Peruvian authors (58.5%). The most frequent main population studied were health professionals (20.4%) and non-clinical stakeholders (20.4%). The majority of studies were conducted in community settings (42.2%). Most studies included only participants from Peru (91.8%). Ethical procedures were not reported in 24.5% of the studies, and 40.8% did not provide an ethics approval code. Adequate SRQR reporting was observed in 32.7% of the included studies (Table 1).

Table 1. Characteristics of included studies (n=147).

**Characteristics**	**n (%)**
Language	
English	72 (49.0)
Spanish	63 (42.8)
English and Spanish	12 (8.2)

Published in a Peruvian journal	
No	130 (88.4)
Yes	17 (11.6)
Number of authors*	5 [4 - 8]
1 to 4	53 (36.1)
4 to 7	55 (37.4)
8 to 36	39 (26.5)
Country of the first author's first affiliation	
Peru	105 (71.4)
United States	27 (18.4)
United Kingdom	4 (2.7)
Switzerland	3 (2.0)
Spain	2 (1.4)
Canada	2 (1.4)
Other countries	4 (2.7)
International collaboration	
Only authors from Peru	86 (58.5)
At least one international author	61 (41.5)
Main population of the study	
Health professionals (clinicians and care personnel)	30 (20.4)
Policy makers, administrators, program managers, community leaders, stakeholders, and other non-clinical populations	30 (20.4)

Patients, service users, and individuals living with health conditions	25 (17.0)
Mothers, fathers, caregivers, and families	22 (15.0)
Children, adolescents, and young people	21 (14.3)
Indigenous peoples, individuals from rural communities, and culturally specific groups	19 (12.9)
Sample size*	15 [12 - 30]
Study setting	
Community settings	62 (42.2)
Health facilities	46 (31.2)
Educational centers	20 (13.6)
Others	18 (12.3)
Not reported	1 (0.7)
Region where the study was conducted†	
Lima	75 (50.3)
Lambayeque	29 (19.5)
Loreto	10 (6.8)
Junin	9 (6.1)
La Libertad	6 (4.1)
Others	37 (25.0)
Not reported	13 (8.8)
Multicountry sample	
Peru only	135 (91.8)
Peru and other countries	12 (8.2)

Reported the ethics committee approval	
Yes	111 (75.5)
No	36 (24.5)
Reported the ethics approval code	
Yes	87 (59.2)
No	60 (40.8)
Total SRQR (in points)*	31 [34 - 38]
Adequate reporting‡	
No	99 (67.3)
Yes	48 (32.7)

SRQR: Standards for Reporting Qualitative Research.

*Median [p25 - p75]

†For this variable, the percentages add up to more than 100% because some studies were conducted in more than one Peruvian region

‡SRQR score (≥ 37 points)

The highest number of qualitative studies were identified in Lima and Lambayeque, with 75 (50.3%) and 29 (19.5%) studies, respectively, while no studies were identified in Apurímac, Huancavelica, and Tumbes (Fig 2).

Figure 2. Number of qualitative studies conducted by region.

The SRQR items with the highest level of adherence were the abstract (S2), reported by 95.9% of studies, the statement of study purpose (S4), reported by 95.2%, and the presentation of main findings (S16), reported by 93.9%. On the other hand, only 31.3% of

studies adequately reported the title elements (S1). Reporting of the qualitative approach or guiding paradigm (S5) was also limited, with 49.0% of studies meeting this criterion. Similarly, researchers' characteristics and reflexivity (S6) were reported in just 37.4% of studies (Fig 3).

Figure 3. Fulfillment of the SRQR items among Peruvian qualitative studies (n=147).

This is the Fig 3 legend

SRQR: Standards for Reporting Qualitative Research

S1. Quality of title

S2. Quality of abstract

S3. Introduction. Background and problem description

S4. Introduction. Study purpose and objectives

S5. Methods: Qualitative approach and research paradigm

S6. Methods: Researcher characteristics and reflexivity

S7. Methods: Study context and setting

S8. Methods: Sampling strategy

S9. Methods: Ethical approval and consent

S10. Methods: Data collection procedures

S11. Methods: Data collection instruments

S12. Methods: Participant characteristics

S13. Methods: Data processing and management

S14. Methods: Data analysis process

S15. Methods: Trustworthiness and credibility techniques

S16. Results: Main findings

S17. Results: Supporting evidence for findings

S18. Discussion: Interpretation and contribution to literature

S19. Discussion: Limitations and trustworthiness

S20. Conflicts of interest

S21. Funding sources

In the regression analysis, some study characteristics were associated with a higher prevalence of adequate SRQR reporting (score ≥ 37). Studies published in Spanish showed a lower frequence of adequate reporting compared with those published in English (PR = 0.12; 95% CI: 0.05–0.32). Studies with eight or more authors (PR = 3.47; 95% CI: 1.81–6.67), studies whose first author was affiliated with a non-Peruvian institution (PR = 0.40; 95% CI: 0.26–0.62), and studies involving international collaboration (PR = 2.35; 95% CI: 1.45–3.82), had a higher prevalence of adequate reporting. In contrast, neither the main population of interest nor publication in a Peruvian journal showed a statistically significant association (Table 2).

Table 2. Factors associated with adequate SRQR checklist reporting (n = 147).

Variables	Adequate SRQR reporting		RP (IC 95%)
	No (SRQR score < 37)	Yes (SRQR score ≥ 37)	
Language of the journal			
English	40 (47.6)	44 (52.4)	Ref.
Spanish	59 (93.7)	4 (6.3)	**0.12 (0.05 to 0.32)**

Published in a Peruvian journal			
No	87 (66.9)	43 (33.1)	Ref.
Yes	12 (70.6)	5 (29.4)	0.89 (0.41 to 1.94)
Number of authors			
1 to 4	44 (83.0)	9 (17.0)	Ref.
4 to 7	39 (70.9)	16 (29.1)	1.71 (0.83 to 3.54)
8 to 36	16 (41.0)	23 (59.0)	**3.47 (1.81 to 6.67)**
Country of the first author's first affiliation			
Non Peru	18 (42.9)	24 (57.1)	Ref
Peru	81 (77.1)	24 (22.9)	**0.40 (0.26 to 0.62)**
International collaboration			
Only authors from Peru	68 (79.1)	18 (20.9)	Ref.
At least one international author	31 (50.8)	30 (49.2)	**2.35 (1.45 to 3.82)**
Main population of the study			
Children, adolescents, and young people	16 (76.2)	5 (23.8)	Ref.
Mothers, fathers, caregivers, and families	25 (83.3)	5 (16.7)	0.70 (0.23 to 2.13)

Indigenous peoples, individuals from rural communities, and culturally specific groups	12 (57.1)	9 (42.9)	1.80 (0.72 to 4.49)
Patients, service users, and individuals living with health conditions	14 (56.0)	11 (44.0)	1.85 (0.76 to 4.49)
Health professionals (clinicians and care personnel)	11 (55.0)	9 (45.0)	1.89 (0.76 to 4.69)
Policy makers, administrators, program managers, community leaders, stakeholders, and other non-clinical populations	21 (70.0)	9 (30.0)	1.26 (0.49 to 3.24)

SRQR: Standards for Reporting Qualitative Research.

PR: Prevalence ratio. 95% CI: 95% confidence interval. Ref: reference.

Discussion

Main results

A total of 147 qualitative studies were included. Most were published in English, had a median of five authors, and were conducted primarily in the capital city and in community settings. First authors were most commonly affiliated with Peruvian institutions, and the majority of studies were conducted exclusively by Peruvian authors. Ethics committee approval was not reported in nearly one quarter of the studies, and almost half did not provide an ethics approval code. The SRQR items with the highest adherence were the

abstract (S2), the statement of study purpose (S4), and the presentation of main findings (S16), whereas title elements (S1), the qualitative approach or guiding paradigm (S5), and researchers' characteristics and reflexivity (S6) were less frequently reported. Adequate SRQR reporting was associated with publication in English, a higher number of authors, non-Peruvian first-author affiliation, and international collaboration, with no significant associations observed for the study population or publication in Peruvian journals.

Characteristics of the studies

Approximately half of the included studies were conducted in Lima, indicating a pronounced geographical concentration of qualitative research in Peru, consistent with prior evidence of higher scientific output in Lima, the capital of Peru [18,19]. This finding suggests that qualitative research follows the pattern of centralized scientific activity in Peru. Barriers to research production in other regions may be driven by structural inequities, such as limited access to research training and funding, as well as the concentration of institutions with greater research capacity in Lima [18]. Strengthening interregional collaboration, revising funding allocation mechanisms, and prioritizing equitable research capacity development may contribute to a more balanced national research landscape [20].

Nearly one quarter of the studies did not report ethical procedures, and over two fifths failed to include an ethics approval code. This finding may reflect two non-mutually exclusive explanations. On the one hand, some studies may not have been reviewed by an ethics committee, which is particularly concerning given that qualitative research typically involves close engagement with participants and may address sensitive topics, thereby requiring ethical oversight to safeguard participant welfare, informed consent, and confidentiality. On the other hand, these results may indicate shortcomings in reporting

practices rather than deficiencies in ethical conduct, whereby ethical approval was obtained but not explicitly documented in the published reports.

Well-established ethical and regulatory frameworks, including the Declaration of Helsinki and the International Ethical Guidelines for Health-related Research Involving Humans issued by CIOMS, as well as current Peruvian regulations [21], stipulate that all research involving human participants must undergo prior review and approval by a research ethics committee. These provisions aim to safeguard participants' rights, safety, and well-being, and to ensure adherence to fundamental ethical principles such as autonomy, confidentiality, and justice.

Although the first author's primary institutional affiliation was predominantly Peruvian (71.4%), a substantial proportion of the studies involved international collaboration (41.5%). This pattern suggests that, despite local leadership in qualitative research production, international collaboration could play a meaningful role in the development of these studies. Such collaborations may reflect efforts to strengthen methodological rigor, access complementary expertise, and enhance the visibility of research conducted in Peru.

Results of the SRQR checklist

The items most consistently reported included the presence of an abstract containing the key elements of the study (S2), the specification of the study objective in the introduction (S4), and the reporting of results, particularly the description of the main findings and the evidence supporting them (S16 and S17). In contrast, the least frequently addressed elements were the use of techniques to ensure the credibility of the findings (S15), the

qualitative approach and research paradigm (S5), the characteristics of the researchers (S6), and the formulation of a title that accurately reflects the nature and focus of the study (S1).

Our findings align with a previous study of qualitative research in pediatric urology, where the abstract and the study objective were also among the most consistently reported items [10]. Moreover, our results are consistent with a previous research on qualitative studies of malaria in Colombia, which identified credibility techniques and the description of the methodological paradigm as among the least reported elements [12]. Another study examining qualitative research in community pharmacies likewise documented poor reporting of the title, researcher characteristics, and the nature of the study [9]. However, unlike those studies, which focused on specific populations or thematic areas, our analysis encompasses the qualitative scientific output of an entire region. This convergence suggests that there is a persistent tendency to report items that are commonly required in scientific manuscripts, irrespective of study design (e.g., abstract key aspects, objective, main findings), reflecting adherence to general reporting conventions rather than specific compliance with qualitative reporting guidelines. By contrast, several items that are fundamental to the rigor and transparency of qualitative research were among the least frequently reported.

This pattern indicates that while general reporting standards are largely met, less emphasis is placed on elements that are distinctive to qualitative inquiry and essential for the appraisal of methodological rigor. In this context, strengthening training in qualitative research reporting, encouraging the adoption of reporting guidelines such as SRQR by journals, and ensuring specialized peer review processes are essential to improve reporting quality.

Factors associated with adequate SRQR reporting

Regarding associated factors, articles published in Spanish were associated with lower reporting quality scores. This may be explained by the tendency of non-English-speaking authors to prioritize publication in English due to its greater international visibility [22] and citation potential [23]. As a result, manuscripts with stronger methodological rigor and better adherence to reporting standards may be more likely to be submitted to, and accepted by, English-language journals. Additionally, studies published in English may more frequently involve international collaborations, which can contribute additional methodological expertise and promote stricter adherence to reporting standards.

Additionally, lower reporting quality was observed in studies whose first author was affiliated with a Peruvian institution, whereas higher reporting quality was found in studies with a larger number of authors and at least one internationally affiliated author. These findings may reflect the potential contribution of international collaboration to improving reporting standards in qualitative research involving Peruvian populations. Previous evidence suggests that international collaboration has a positive impact on research quality [24,25], particularly in less well-established research systems [26], such as Peru.

Limitations and strengths

This study has some limitations. First, mixed-methods studies were not included, as their methodological quality is assessed using specific appraisal tools, such as the Mixed Methods Appraisal Tool (MMAT), which fall outside the scope of the present evaluation [27]. Second, the search was restricted to Scopus, excluding regional databases such as SciELO or LILACS due to their limitation in filtering results by affiliation in these repositories. Although Scopus allows the identification of studies with affiliation with Peruvian institutions, this restriction may have omitted qualitative research published in journals with a local scope. Third, the analysis was limited to the 2022–2025 period, to

focus on recent studies and reduce potential bias related to the accelerated publication processes during the COVID-19 pandemic [28]. While this timeframe may have limited the inclusion of relevant scientific production, this scope allows for an evaluation of the current state of qualitative research in Peru. Fourth, although the SRQR provides a structured framework for evaluating reporting quality, the interpretation of some items may involve a degree of reviewer subjectivity. Also, because the SRQR evaluates reporting quality rather than intrinsic methodological rigor, some studies may have received higher or lower scores based on reporting completeness rather than the strength of their design. Finally, the regression analysis estimated crude associations; therefore, the observed relationships should be interpreted cautiously and should not be interpreted as evidence of causal relationships.

On the other hand, this study also has some strengths. First, flexibility was applied in the interpretation of certain SRQR items to avoid ratings being influenced by editorial decisions rather than the absence of relevant content. For instance, some articles did not present a structured abstract (assessed under item 2) yet contained all expected elements and were therefore awarded full points. Similarly, statements regarding reflexivity and ethical considerations located outside the methods section were accepted as valid. Second, study selection, data extraction, and analysis were conducted independently by pairs of reviewers, with discrepancies resolved by a third or even fourth evaluator, thereby reducing the risk of individual bias and strengthening the reliability of the assessment process. Third, for data extraction, each item was assigned a score, and this score was accompanied by a justification, which allowed for greater discussion among the authors and increased rigor in the assignment of the scores. Finally, the use of a widely recognized instrument such as the SRQR, whose structure allows for the assessment of items applicable across multiple methodological approaches within qualitative research [29].

Conclusion

This review identifies key areas for improvement in the reporting of qualitative health studies in the Peruvian population, including techniques to ensure trustworthiness and credibility, methodological approach, researcher reflexivity, title quality, and reporting of ethics approval. Editorial teams could encourage adherence to standardized reporting guidelines and make editorial policies more explicit regarding ethics committee approval and key elements of methodological transparency, thereby supporting improved reporting practices. Future research could investigate whether these challenges persist in other contexts with similar structural and geographical disparities.

Conflict of interest statement:

The authors declare no conflicts of interest related to this study.

Acknowledgments:

None.

Data availability statement:

The raw data underlying the findings are available in the S4 Table.

References

1. Kuper A, Reeves S, Levinson W. An introduction to reading and appraising qualitative research. *BMJ*. 2008;337:a288. doi:10.1136/bmj.a288
2. Huston P, Rowan M. Qualitative studies. Their role in medical research. *Can Fam Physician Med Fam Can*. 1998;44:2453-2458.

3. Renjith V, Yesodharan R, Noronha JA, Ladd E, George A. Qualitative Methods in Health Care Research. *Int J Prev Med*. 2021;12:20. doi:10.4103/ijpvm.IJPVM_321_19

4. Dunt D, McKenzie R. Improving the quality of qualitative studies: do reporting guidelines have a place? *Fam Pract*. 2012;29(4):367-369. doi:10.1093/fampra/cms041

5. Mays N, Pope C. Qualitative research in health care. Assessing quality in qualitative research. *BMJ*. 2000;320(7226):50-52. doi:10.1136/bmj.320.7226.50

6. O'Brien BC, Harris IB, Beckman TJ, Reed DA, Cook DA. Standards for reporting qualitative research: a synthesis of recommendations. *Acad Med J Assoc Am Med Coll*. 2014;89(9):1245-1251. doi:10.1097/ACM.0000000000000388

7. Tong A, Sainsbury P, Craig J. Consolidated criteria for reporting qualitative research (COREQ): a 32-item checklist for interviews and focus groups. *Int J Qual Health Care J Int Soc Qual Health Care*. 2007;19(6):349-357. doi:10.1093/intqhc/mzm042

8. Tong A, Flemming K, McInnes E, Oliver S, Craig J. Enhancing transparency in reporting the synthesis of qualitative research: ENTREQ. *BMC Med Res Methodol*. 2012;12:181. doi:10.1186/1471-2288-12-181

9. Aref HAT, Witry M, Olufemi-Yusuf D, Guirguis LM. Ensuring quality qualitative research reporting in community pharmacy: a systematic literature review. *Int J Pharm Pract*. 2021;29(5):416-427. doi:10.1093/ijpp/riab027

10. McCloskey K, Neuzil K, Basak R, Chan KH. Quality of reporting for qualitative studies in pediatric urology—A scoping review. *J Pediatr Urol*. 2023;19(5):643-651. doi:10.1016/j.jpurol.2023.04.027

11. Silva GS, Fernandes D de RF, Alves CRL. Avaliação da assistência à saúde da criança na Atenção Primária no Brasil: revisão sistemática de métodos e resultados. *Ciênc Saúde Coletiva*. 2020;25:3185-3200. doi:https://doi.org/10.1590/1413-81232020258.27512018

12. Cardona-Arias JA, Salas-Zapata W, Carmona-Fonseca J. Systematic review of qualitative studies about malaria in Colombia. *Heliyon*. 2020;6(5). doi:10.1016/j.heliyon.2020.e03964

13. Angarita FA, Zhang Y, Elmi M, Hong NJL. Older women's experience with breast cancer treatment: A systematic review of qualitative literature. *The Breast*. 2020;54:293-302. doi:10.1016/j.breast.2020.11.009

14. Mendoza-Chuctaya G, Chachaima-Mar JE, Mejia CR, et al. Análisis de producción, impacto y redes de colaboración en investigaciones científicas en Scopus en Perú de 2000 a 2019. *Medwave*. 2021;21(2). doi:10.5867/medwave.2021.02.8121

15. Carcausto-Calla WH, Morales-Quispe J. Investigaciones cualitativas en salud publicadas en revistas biomédicas peruanas. *An Fac Med*. 2018;79(2):144-148. doi:10.15381/anales.v79i2.14941

16. Watson A, Jackson D. The Importance of Analytic Integrity and Reporting Guidelines in Reporting Qualitative Research. *J Adv Nurs*. 2025;81(7):3425-3427. doi:10.1111/jan.16617

17. Tricco AC, Lillie E, Zarin W, et al. PRISMA Extension for Scoping Reviews (PRISMA-ScR): Checklist and Explanation. *Ann Intern Med*. 2018;169(7):467-473. doi:10.7326/M18-0850

18. Arcila-Diaz J, Delgado-Caramutti J, Millones-Gómez PA, Figueroa-Quiñones J, Valencia-Arias A. Research trends in Peruvian universities: proposal for a research agenda with a bibliometric approach. *Scientometrics*. 2025;130(1):489-514. doi:10.1007/s11192-024-05211-z

19. Vásquez-Uriarte K, Roque-Henriquez JC, Angulo-Bazán Y, Ninatanta Ortiz JA. Análisis bibliométrico de la producción científica peruana sobre la COVID-19. *Rev Peru Med Exp Salud Pública*. 2021;38:224-231. doi:https://doi.org/10.17843/rpmesp.2021.382.7470

20. Choquez-Millan MF, Lechtape CL, Löhr K, Schröter B, Graef F. Uncovering power asymmetries in North-South research collaborations – An example from sustainability research in Tanzania. *Futures*. 2024;156:103316. doi:10.1016/j.futures.2023.103316

21. Resolución Ministerial N.° 233-2020-MINSA. Accessed January 8, 2026. https://www.gob.pe/institucion/minsa/normas-legales/541139-233-2020-minsa

22. Stockemer D, Wigginton MJ. Publishing in English or another language: An inclusive study of scholar's language publication preferences in the natural, social and interdisciplinary sciences. *Scientometrics*. 2019;118(2):645-652. doi:10.1007/s11192-018-2987-0

23. Di Bitetti MS, Ferreras JA. Publish (in English) or perish: The effect on citation rate of using languages other than English in scientific publications. *Ambio*. 2017;46(1):121-127. doi:10.1007/s13280-016-0820-7

24. Kohus Z, Demeter M, Szigeti GP, Kun L, Lukács E, Czakó K. The Influence of International Collaboration on the Scientific Impact in V4 Countries. *Publications*. 2022;10(4):1-13. doi: 10.3390/publications10040035

25. Wang J, Frietsch R, Neuhäusler P, Hooi R. International collaboration leading to high citations: Global impact or home country effect? *J Informetr*. 2024;18(4):101565. doi:10.1016/j.joi.2024.101565

26. Farias Borges LF, Fischer B, Mazoni A, Bascur JP. The complex interplay between international collaboration and scientific impact: evidence from the Scopus database. *Cogent Educ*. 2025;12(1):2563708. doi:10.1080/2331186X.2025.2563708

27. Hong QN, Fàbregues S, Bartlett G, et al. The Mixed Methods Appraisal Tool (MMAT) version 2018 for information professionals and researchers. *Educ Inf*. 2018;34(4):285-291. doi:10.3233/EFI-180221

28. McDermott KT, Perry M, Linden W, Croft R, Wolff R, Kleijnen J. The quality of COVID-19 systematic reviews during the coronavirus 2019 pandemic: an exploratory comparison. *Syst Rev*. 2024;13(1):126. doi:10.1186/s13643-024-02552-x

29. Dossett LA, Kaji AH, Cochran A. SRQR and COREQ Reporting Guidelines for Qualitative Studies. *JAMA Surg*. 2021;156(9):875-876. doi:10.1001/jamasurg.2021.0525

Supporting information

S1 Table. Preferred Reporting Items for Systematic reviews and Meta-Analyses extension for Scoping Reviews (PRISMA-ScR) checklist.

S2 Table. Characteristics of the included studies (n=147)

S3 Table. Table of excluded studies (n=55)

S4 Table. Extracted dataset

Click here to access/download;Figure;Fig1.tif.tif

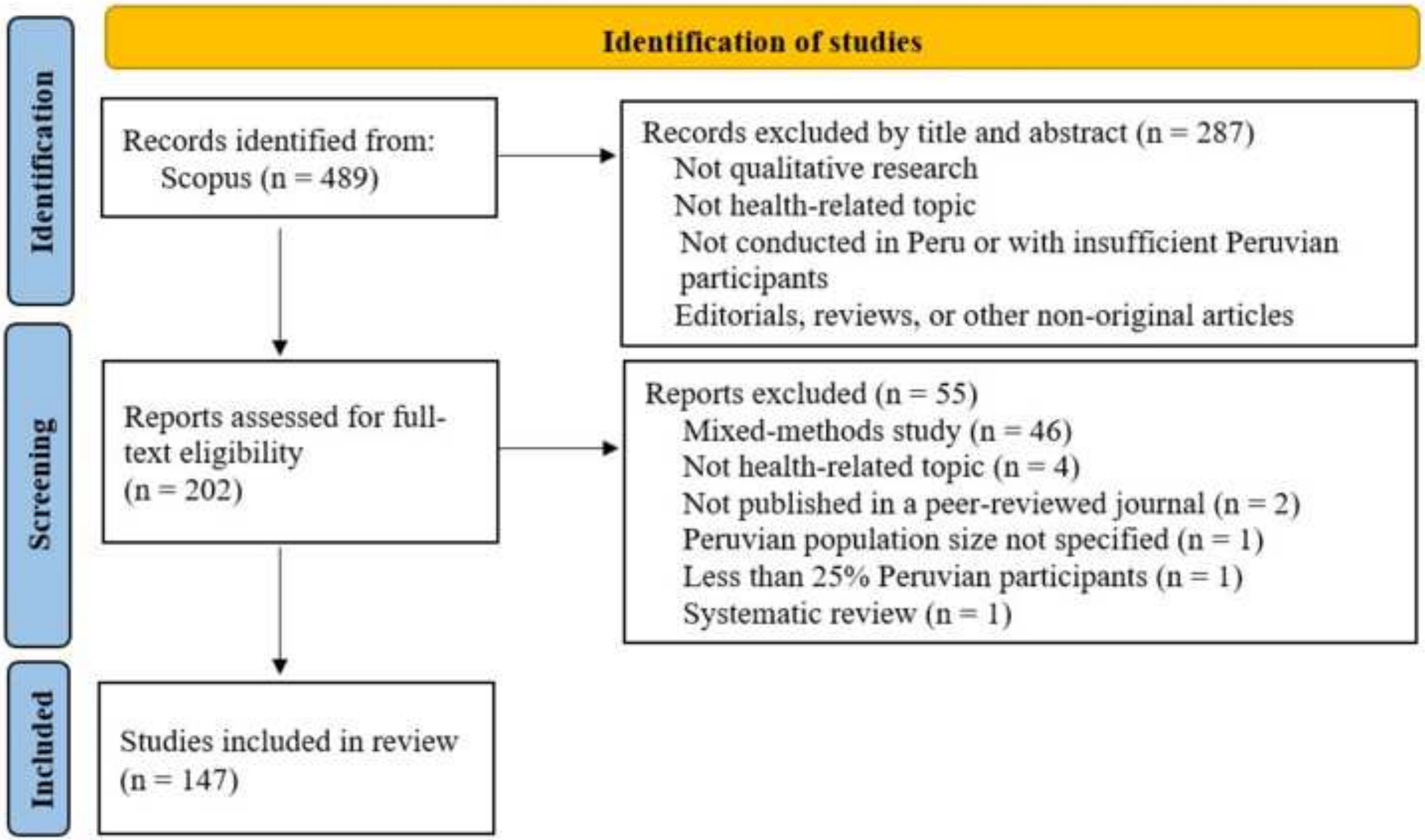

Figure 2

Click here to access/download;Figure;Fig2.tif.tif

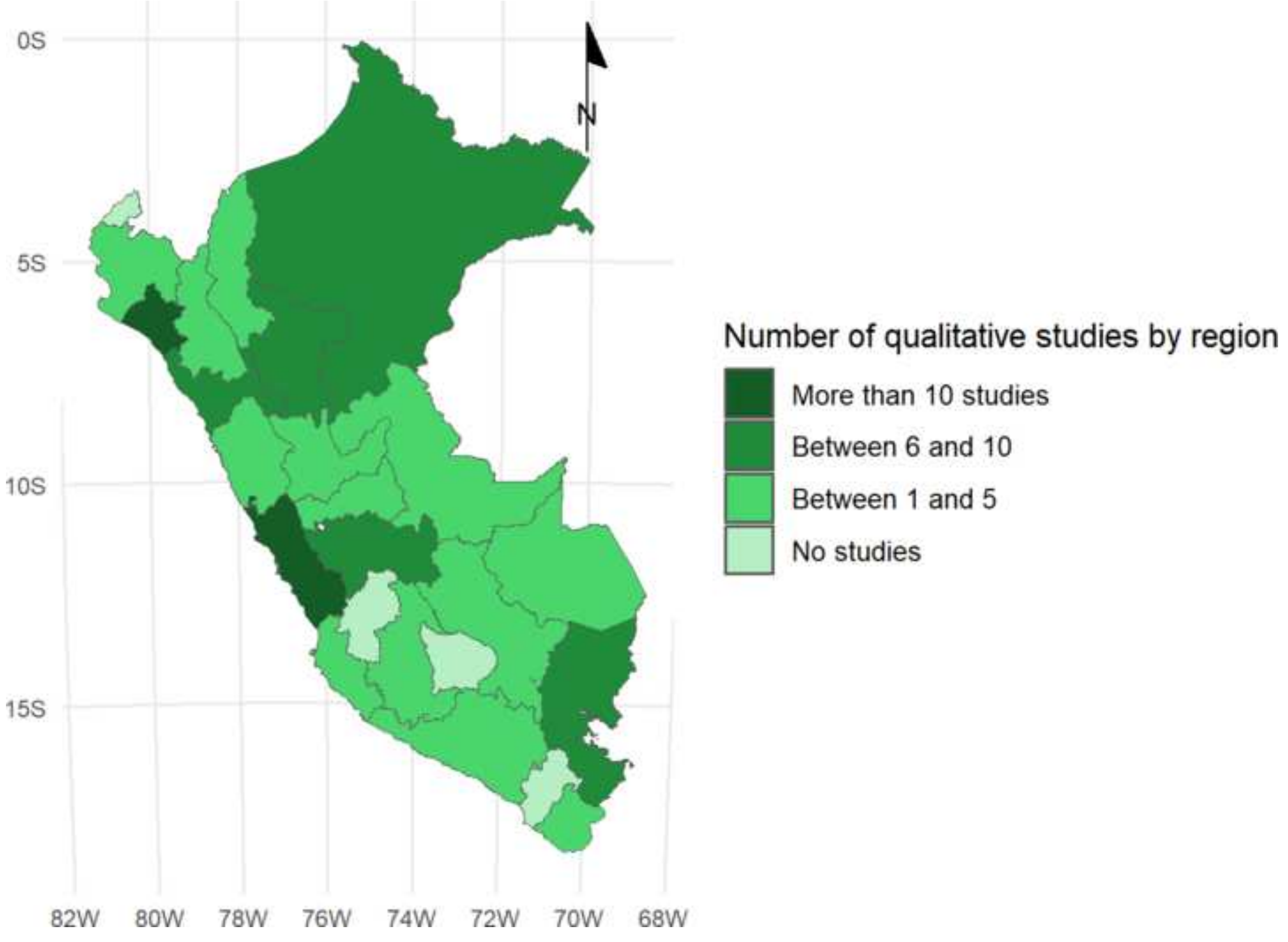

Figure 3

Click here to access/download;Figure;Fig3.tif.tif

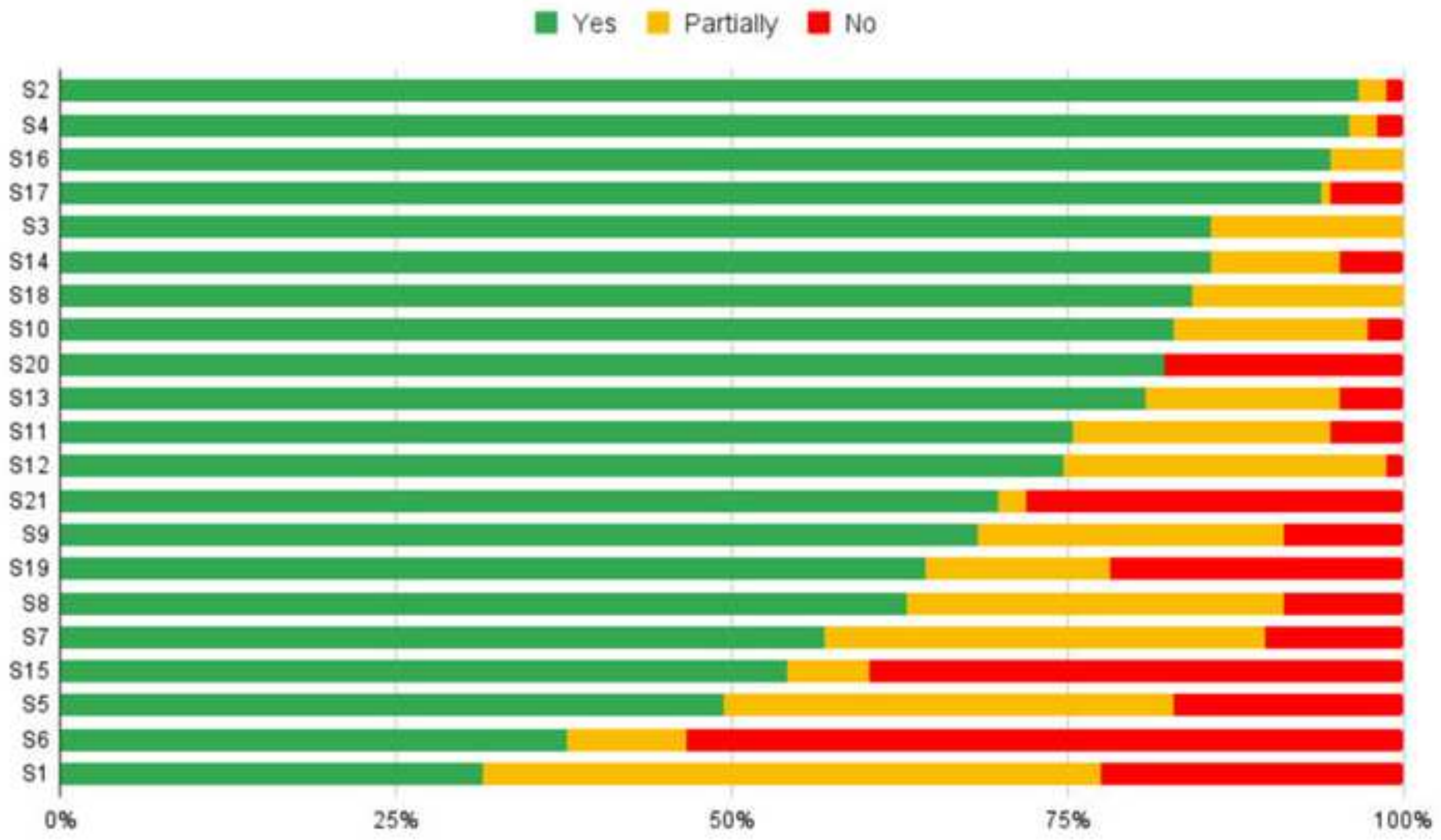

Supporting Information 1

Click here to access/download
Supporting Information
S1_Table.doc.docx

Supporting Information 2

Click here to access/download
Supporting Information
S2_Table.doc.docx

Click here to access/download
Supporting Information
S3_Table.doc.docx

Click here to access/download
Supporting Information
S4_File.xls.xlsx

Reporting quality of qualitative health studies published by Peruvian authors: a scoping review

Authors

Cristhian Rojas-Miliano[1,2], Andrea Dámaso-Román[3], Guillermo Almeida-Huanca[4], Nikol Mayo-Puchoc[5], Yolanda Viguria-Chavez[6], Andy Rick Sánchez-Villena[7], Alvaro Taype-Rondan[8,9], Rubí Paredes-Angeles[10*]

* (1,2) Andrea Dámaso-Román (3)Guillermo Almeida-Huanca (4)

alexrojasmiliano@gmail.com https://orcid.org/0000-0002-3364-4940

Andrea Dámaso-Román (3)[1]

andrea.damaso@unmsm.edu.pe https://orcid.org/0000-0002-8150-5777

Guillermo Almeida-Huanca (4)

guillermoalmeida1604@gmail.com https://orcid.org/0000-0003-0283-3586

Nikol Mayo-Puchoc (5)

nikol.mayo@upch.pe https://orcid.org/0000-0002-6182-7605

Yolanda Viguria-Chavez (6)

yolanda.viguria.c@upch.pe https://orcid.org/0000-0002-3339-112X

Andy Rick Sánchez-Villena (7)

andysavi92@gmail.com https://orcid.org/0000-0001-7828-5884

Alvaro Taype-Rondan (8,9)

alvaro.taype.r@gmail.com https://orcid.org/0000-0001-8758-0463

Rubí Paredes-Angeles (10)

rubiparedesa@gmail.com https://orcid.org/0000-0003-3669-4932

Formatted
Formatted: Font: Italic
Formatted: Font: 14 pt
Formatted: Heading 1, Justified
Formatted: Font: 14 pt, Not Bold
Formatted: Spanish (Peru)
Formatted: Indent: Left: -0.1"
Formatted: Superscript
Formatted: Spanish (Peru)
Formatted: Spanish (Peru)
Formatted: Indent: Left: 0"
Formatted: Indent: Left: -0.1"
Formatted: Spanish (Peru)
Formatted: Spanish (Peru)
Formatted: Spanish (Peru)
Formatted: Spanish (Peru)
Formatted: Spanish (Peru)
Formatted: Spanish (Peru)
Formatted: Spanish (Peru)
Formatted: Spanish (Peru)
Formatted: Spanish (Peru)
Formatted: Spanish (Peru)
Formatted: Spanish (Peru)
Formatted: Spanish (Peru)
Formatted: Spanish (Peru)
Formatted: Spanish (Peru)

Affiliations

1) Universidad Nacional del Centro del Perú, Junín, Perú

2) [2]Centro de Salud Mental Comunitario Pangoa, Junín, Perú

3) [3]Universidad Nacional Mayor de San Marcos, Facultad de Psicología, Lima, Perú

4) [4]Grupo de Investigación en Psicología Social y Desarrollo Humano, Facultad de Psicología,-Universidad Nacional Mayor de San Marcos, Lima, Perú

5) [5]Instituto Peruano de Orientación Psicológica, Lima, Perú

6) [6]Universidad Peruana Cayetano Heredia, School of Public Health, Lima, Perú

7) [7]Universidad Señor de Sipán, Vicerrectorado de Investigación, Chiclayo, Perú

8) [8]Unidad de Investigación para la Generación y Síntesis de Evidencias en Salud, Vicerrectorado de Investigación, Universidad San Ignacio de Loyola, Lima, Perú

9) [9]EviSalud – Evidencias en Salud, Lima, Perú

10) [10]Universidad Científica del Sur, Lima, Perú

*Corresponding author:

Rubi Paredes-Angeles

Phone number: +51 964772360

E-mMail: rubiparedesa@gmail.com (PAR)

Universidad Científica del Sur, Lima, Peru

Author contributions:

Conceptualization: Rubi Paredes-Angeles, Alvaro Taype-Rondan

Data Curation: Cristhian Rojas-Miliano, Andrea Dámaso-Román, Guillermo Almeida-Huanca, Nikol Mayo-Puchoc, Yolanda Viguria-Chavez, Andy Rick Sánchez-Villena

Formatted: Indent: Left: -0.1", Space Before: 0 pt, After: 0 pt

Formatted: Indent: Left: -0.1", No bullets or numbering

Formatted: English (United States)

Formatted: Font: Not Bold

Formatted: Indent: Left: -0.1"

Field Code Changed

Formatted: English (United States)

Formatted: English (United States)

Formatted: Indent: Left: 0"

~~**Formal Analysis**: Rubi Paredes-Angeles~~

~~**Investigation:** Cristhian Rojas-Miliano, Andrea Dámaso-Román, Guillermo Almeida-Huanca, Nikol Mayo-Puchoc, Yolanda Viguria-Chavez, Andy Rick Sánchez-Villena~~

~~**Methodology:** Rubi Paredes-Angeles Alvaro Taype-Rondan~~

~~**Writing – Original Draft Preparation:** Cristhian Rojas-Miliano, Andrea Dámaso-Román, Guillermo Almeida-Huanca, Nikol Mayo-Puchoc, Yolanda Viguria-Chavez, Andy Rick Sánchez-Villena, Rubi Paredes-Angeles Alvaro Taype-Rondan~~

~~**Writing – Review & Editing:** Cristhian Rojas-Miliano, Andrea Dámaso-Román, Guillermo Almeida-Huanca, Nikol Mayo-Puchoc, Yolanda Viguria-Chavez, Andy Rick Sánchez-Villena, Rubi Paredes-Angeles Alvaro Taype-Rondan~~

Abstract

Formatted: Font: 18 pt

Formatted: Indent: Left: -0.1"

Qualitative research offers valuable insights into complex social and behavioral processes in the health sciences; however, its contribution depends on the quality and transparency of reporting. This scoping review aimed to describe the methodological characteristics and reporting quality of qualitative studies published by Peruvian authors between 2022 and 2025. Following PRISMA-ScR guidelines, a systematic search was conducted in SCOPUS, and reporting quality was assessed using the Standards for Reporting Qualitative Research (SRQR) tool. A total of 147 qualitative studies were included. Most were published in English (49.0%), had a median of five authors; 71.4% had a first author affiliated with a Peruvian institution, and 58.5% involved exclusively Peruvian authors. Studies were mainly conducted in Lima (50.7%). Ethics committee approval was not reported in 24.5% of studies, and 40.8% did not provide an ethics approval code. Adherence to SRQR items was highest for abstracts (95.9%), study purpose (95.2%), and presentation of findings (93.9%), whereas reporting of title elements (31.3%), qualitative approach (49.0%), and researcher reflexivity (37.4%) was limited. Publication in Spanish and Peruvian first authorship were associated with lower SRQR scores, whereas a higher number of authors and international collaboration were associated with higher scores. Overall, qualitative studies published by Peruvian authors showed variable reporting quality, with important methodological and ethical aspects frequently underreported, highlighting the need to strengthen reporting practices, particularly for core qualitative components.

Key words: Qualitative Research; Reporting quality; Standards for Reporting Qualitative Research; Scoping review; Peru.

Introduction

Formatted: Indent: Left: -0.1"

Qualitative research provides a deeper understanding of complex social and behavioral processes in health sciences [1][1]. Unlike the quantitative methods, qualitative studies seek to explore, narrate and explain experiences, perceptions, and contexts, offering a transformative perspective that enriches evidence-based decision-making [2,3][2,3]. Thus, qualitative research enhances the contextual understanding necessary for developing grounded health interventions [1,2][1,2].

However, the usefulness of qualitative findings can be affected by the quality of reporting [4][4]. Although the inherent heterogeneity of qualitative research reporting reflects its richness and diversity, this variability can also impede systematic evaluation of quality and comparison across studies [5][5]. Clear, complete, and transparent reporting enables readers to better assess the credibility, transferability, and methodological rigor of qualitative studies.

Some international guidelines have been developed to standardize reporting in qualitative research, including the Standards for Reporting Qualitative Research (SRQR) [6][6], the Consolidated Criteria for Reporting Qualitative Research (COREQ) –[7][7], and the Enhancing Transparency in Reporting the synthesis of Qualitative research (ENTREQ) [8][8]. Unlike COREQ, which is tailored for interviews and focus groups, and ENTREQ, which is focused on qualitative synthesis studies, SRQR provides a comprehensive framework that authors can use across a wide range of qualitative methodologies [6,9][6,9]. Thus, previous research has employed the SRQR to evaluate reporting quality in qualitative studies conducted in diverse health domains, including urology [10][10], pediatrics [11][11], infectious diseases [12][12], and breast cancer [13][13].

In Peru, hscientific research output has increased, with an estimated annual growth rate of 13.6%, with 70.2% corresponds to health sciences [14]ealth research publications have increased [14]. A previous study examined the scientific production of qualitative research in Peru found that the publication trend has increased since 2013; however, it did not assess the reporting quality of these studies [15][15]. ; however, to date, no studies have specifically examined qualitative health research or assessed the quality of its reporting. The absence of such information limits understanding of the transparency and reporting standards within this research field in Peru. Assessing these aspects may help identify strengths, weaknesses, and opportunities for improvement among researchers, reviewers, and editors of scientific journals [16][16]. Therefore, this study aimed to describe the methodological characteristics and assess the reporting quality of qualitative studies conducted in populations residing in Peru and involving at least one author affiliated with a published by Peruvian institutionauthors.

Formatted: English (United States)

Formatted: English (United States)

Methods

This scoping review was carried out and reported following the guidelines of the Preferred Reporting Items for Systematic Reviews and Meta-Analyses Extension for Scoping Reviews (PRISMA-ScR) [17][17]. The completed PRISMA-ScR checklist is provided in S1 Table **Supplementary Material 1**. A detailed study protocol was developed prior to study initiation and made publicly available. (The protocol of this review is available at https://osf.io/jes6v/files/3rn52).

Formatted: Font color: Hyperlink

Field Code Changed

Search strategy

A systematic search was performed in Scopus on October 15, 2025, using the following search strategy to identify qualitative health studies authored by researchers affiliated with institutions in Peru and published between 2022 and 2025:

AFFILCOUNTRY (peru) AND TITLE-ABS (qualitative) AND ((SUBJAREA (MEDI OR NURS OR DENT OR HEAL) OR SUBJAREA (PSYC))) AND PUBYEAR>2021 AND PUBYEAR < 2026

Study eligibility

We included original articles authored by researchers affiliated with institutions in Peru, that were published in scientific journals between 2022 and 2025 and presented findings from qualitative studies. This timeframe was chosen to reflect the current state of the field.

Only studies in which at least 25% of participants were based in Perubased in Peru were considered to ensure meaningful representation of the Peruvian population while allowing the inclusion of international studies.. Publications relatedStudies related to health disciplines, including medicine, nursing, dentistry, psychology, and other health professions, were included. Mixed-methods studies were excluded due to the need for specific assessment tools. No language restrictions were applied.

Studies selection

Formatted: Font: 12 pt, Not Bold

Records were uploaded to the "Rayyan" software (Qatar Computing Research Institute, Doha, Qatar; -https://www.rayyan.ai/). Six authors in pairs independently screened the records applying the selection criteria. Disagreements between the reviewers were resolved through discussion among the reviewers or with the collaboration of another authors.

Formatted: Font: 12 pt, English (United States), Reflection

Data extraction

To ensure consistency, a pilot data extraction was conducted prior to the main process. Subsequently, six authors worked in pairs to independently extract data from each study, recording all information in a predesigned Excel spreadsheet. Any discrepancies were reviewed and resolved with input from a fifth author. The research team consisted of professionals in Medicine and Psychology with experience in conducting health research.

For each included study, we collected information on its general and methodological characteristics, including: language of the study (English, Spanish, or both English and Spanish), whether the study was published in a Peruvian journal (yes or no), number of authors (divided in tertiles), country of the first author's institutional affiliation, international collaboration, defined as the inclusion of at least one co-author with an institutional affiliation in a country other than Peru (yes or no), study population, sample size, study setting (community settings, health facilities or educational centers), Peruvian region where the study was conducted, multicountry study, defined as a study that included research sites in countries other than Peru (only Peru or Peru and others countries), ethics committee approval (whether the article reported approval from at least one ethics committee), and ethics approval code reported (whether the article, supplementary material, or appendix included the ethics approval code).

Additionally, we assessed the quality of reporting using the SRQR checklist. The data extraction matrix included two components for the SRQR score: one for the assigned score and another to document the rationale, including verbatim excerpts from the reviewed articles to justify each rating. Each pair of investigators independently extracted study characteristics and SRQR items, and then met to compare each cell and reach consensus, resulting in a final extraction. Discrepancies that could not be resolved were discussed during weekly team meetings until agreement was achieved.

Formatted: Indent: Left: -0.1"

Quality of reporting

We used the SRQR 21-item checklist- [6][6], which provides guidance on transparency and completeness in qualitative research reporting. Each of the 21 items was assessed to determine whether it was 'reported' (score = 2 points), 'partially reported' (score = 1 point), or 'not reported' (score = 0 points).

The total SRQR score ranged from 0 to 42 points. An item was considered 'reported' when the authors presented the required information in accordance with the SRQR guidelines, 'partially reported' when the authors provided some, but not all, of the required information, and 'not reported' when the authors failed to provide any of the required information according to the SRQR reporting standard- [9][9]. Also, we created the variable "adequate SRQR reporting," defined as belonging to the highest tertile (≥ 37 points) of the SRQR score. Given the absence of established or validated cut-off points for SRQR scores, this data-driven approach was used to identify studies with comparatively better reporting quality.

This SRQR checklist assessed the following items:

S1. Title. Concise description of the nature and topic of the study. Identifying the study as qualitative/mixed or indicating the approach (e.g., ethnography, grounded theory) or data collection methods (e.g., interview, focus group) is recommended.

S2. Abstract. Summary of key elements of the study using the abstract format of the intended publication; typically includes background, purpose, methods, results, and conclusions.

S3. Introduction. Description and significance of the problem/phenomenon studied; review of relevant theory and empirical work problem statement.

S4. Introduction. Purpose of the study and specific objectives or questions.

S5. Methods. Qualitative approach (e.g., ethnography, grounded theory, case study, phenomenology, narrative research) and guiding theory if appropriate; identifying the research paradigm (e.g., postpositivist, constructivist/ interpretivist) is also recommended; rationale.

S6. Methods. Researchers' characteristics that may influence the research, including personal attributes, qualifications/experience, relationship with participants, assumptions, and/or presuppositions; potential or actual interaction between researchers' characteristics and the research questions, approach, methods, results, and/or transferability.

S7. Methods. Context. Setting/site and salient contextual factors; rationale.

S8. Methods. Sampling strategy. How and why research participants, documents, or events were selected; criteria for deciding when no further sampling was necessary (e.g., sampling saturation); rationale

S9. Methods. Documentation of approval by an appropriate ethics review board and participant consent, or explanation for lack thereof; other confidentiality and data security issues

S10. Methods. Types of data collected; details of data collection procedures including (as appropriate) start and stop dates of data collection and analysis, iterative process, triangulation of sources/methods, and modification of procedures in response to evolving study findings; rationale

S11. Methods. Description of instruments (e.g., interview guides, questionnaires) and devices (e.g., audio recorders) used for data collection; if/how the instrument(s) changed over the course of the study

S12. Methods (or results). Number and relevant characteristics of participants, documents, or events included in the study; level of participation (could be reported in results)

S13. Methods. Methods for processing data prior to and during analysis, including transcription, data entry, data management and security, verification of data integrity, data coding, and anonymization/deidentification of excerpts"

S14. Methods. Process by which inferences, themes, etc., were identified and developed, including the researchers involved in data analysis; usually references a specific paradigm or approach; rationale

S15. Methods. Techniques to enhance trustworthiness and credibility of data analysis (e.g., member checking, audit trail, triangulation); rationale

S16. Results. Main findings (e.g., interpretations, inferences, and themes); might include development of a theory or model, or integration with prior research or theory

S17. Results. Evidence (e.g., quotes, field notes, text excerpts, photographs) to substantiate analytic findings

S18. Discussion. Short summary of main findings; explanation of how findings and conclusions connect to, support, elaborate on, or challenge conclusions of earlier scholarship; discussion of scope of application/generalizability; identification of unique contribution(s) to scholarship in a discipline or field

S19. Discussion. Trustworthiness and limitations of findings

S20. Conflicts of interest. Potential sources of influence or perceived influence on study conduct and conclusions; how these were managed

S21. Funding. Sources of funding and other support; role of funders in data collection, interpretation, and reporting.

Data analysis

Data were entered into Microsoft Excel, and all statistical analyses were performed using Stata v19.0 software, version 19.0 (StataCorp, College Station, TX, USA; . https://www.stata.com/). Descriptive analyses included absolute and relative frequencies for categorical variables and medians with interquartile ranges for quantitativenumeric variables. Additionally, an exploratory analysis of factors associated with adequate SRQR reporting was conducted. For this purpose, crude prevalence ratios (PR) with 95% confidence intervals (CI) were estimated using Poisson regression with robust variance.

Spatial data processing and visualization were performed in R (version 4.2.2; R Foundation for Statistical Computing, Vienna, Austria). Administrative boundary data for Peru were obtained from the "mapsPERU" package (version 2.0.0), which provides publicly available vector-based geospatial data. Spatial data were managed using the "sf" package (version 1.0.16). Data manipulation was conducted using "dplyr" package (version 1.1.4) and "ggplot2" package (version 3.5.1). No proprietary basemaps were used.

Formatted: Indent: Left: -0.1"

Ethical statement:

Ethics committee approval was not required because this study involved only a review of previously published literature and did not include human participants.

Results

The search yielded 489 records, of which 202 were selected for full-text assessment after duplicate removal and title and abstract screening. Records were excluded at this stage

because they did not meet the eligibility criteria, including studies that were not qualitative, were not related to health sciences, did not involve populations residing in Peru, or corresponded to non-original publications. Of these, 55 reports were excluded after full-text assessment for the following reasons: mixed-methods design (n = 46), non–health-related topic (n = 4), publication outside a peer-reviewed journal (n = 2), unspecified Peruvian population size (n = 1), inclusion of less than 25% Peruvian participants (n = 1), and systematic review design (n = 1). Consequently, a total of 147 studies were included in the final analysis (Figgure 1). The characteristics of the included studies can be found in S2 Table Supplementary Material 2, while the list of excluded reports is presented in S3 Table Supplementary Material 3.

Figure 1. Flowchart of the article selection process.

Across the included studies, most were published in English (49.0%) and were not published in a Peruvian journal (88.4%). The median number of authors was five, and 37.4% were studies with four to seven authors. Regarding affiliation, the first author most frequently had their primary institutional affiliation in Peru (71.4%), and most studies involved only Peruvian authors (58.5%). The most frequent main population studied were health professionals (20.4%) and non-clinical stakeholders (20.4%). The majority of studies were conducted in community settings (42.2%). Most studies included only participants from Peru (91.8%). Ethical procedures were not reported in 24.5% of the studies, and 40.8% did not provide an ethics approval code. Adequate SRQR reporting was observed in 32.7% of the included studies (Table 1).

Table 1. Characteristics of included studies (n=147).

Formatted: Font: Not Bold

Formatted: Font: Not Bold

Formatted: Font: Not Bold

Formatted: Font: Not Bold

Formatted: Indent: Left: -0.1", Line spacing: Double

Formatted: Indent: Left: -0.1"

Formatted: Font: Not Bold

Formatted: Indent: Left: -0.1", Line spacing: Double

**Characteristics**	**n (%)**
Language	
_English	72 (49.0)
_Spanish	63 (42.8)
_English and Spanish	12 (8.2)
Published in a Peruvian journal	
_No	130 (88.4)
_Yes	17 (11.6)
Number of authors*	5 [4 - 8]
_1 to 4	53 (36.1)
_4 to 7	55 (37.4)
_8 to 36	39 (26.5)
Country of the first author's first affiliation	
_Peru	105 (71.4)
_United States	27 (18.4)
_United Kingdom	4 (2.7)
_Switzerland	3 (2.0)
_Spain	2 (1.4)
_Canada	2 (1.4)
_Other countries	4 (2.7)
International collaboration	
_Only authors from Peru	86 (58.5)
_At least one international author	61 (41.5)
Main population of the study	

Health professionals (clinicians and care personnel)Children, adolescents, and young people	30 (20.4)21 (14.3)
Policy makers, administrators, program managers, community leaders, stakeholders, and other non-clinical populations	30 (20.4)
Patients, service users, and individuals living with health conditions	25 (17.0)
_Mothers, fathers, caregivers, and families	22 (15.0)
Children, adolescents, and young people	21 (14.3)
_Indigenous peoples, individuals from rural communities, and culturally specific groups	19 (12.9)
Patients, service users, and individuals living with health conditions	25 (17.0)
Health professionals (clinicians and care personnel)	30 (20.4)
Policy makers, administrators, program managers, community leaders, stakeholders, and other non-clinical populations	30 (20.4)
Sample size*	15 [12 - 30]
Study setting	
Not reported	1 (0.7)
_Community settings	62 (42.2)
_Health facilities	46 (31.2)
_Educational centers	20 (13.6)
_Others	18 (12.3)

Formatted: Indent: Left: 0.01", Line spacing: Double
Formatted: Line spacing: Double
Formatted: English (United States)
Formatted: Indent: Left: 0.01", Line spacing: Double
Formatted: Line spacing: Double
Formatted: Indent: Left: 0.01", Line spacing: Double
Formatted: Line spacing: Double
Formatted: Indent: Left: 0.01", Line spacing: Double
Formatted: Line spacing: Double
Formatted: Indent: Left: 0.01", Line spacing: Double
Formatted: Line spacing: Double
Formatted: Indent: Left: 0", Line spacing: Double
Formatted: Line spacing: Double
Formatted: Indent: Left: 0", Line spacing: Double
Formatted: Line spacing: Double
Formatted: Indent: Left: 0", Line spacing: Double
Formatted: Line spacing: Double
Formatted: Indent: Left: 0", Line spacing: Double
Formatted: Line spacing: Double
Formatted: Indent: Left: 0", Line spacing: Double
Formatted: Line spacing: Double
Formatted: Indent: Left: 0", Line spacing: Double
Formatted: Line spacing: Double
Formatted: Indent: Left: 0", Line spacing: Double
Formatted: Line spacing: Double
Formatted: Indent: Left: 0", Line spacing: Double
Formatted: Line spacing: Double

Not reported	1 (0.7)
Region where the study was conducted†	
LimaNot reported	75 (50.3)13 (8.8)
Lima	75 (50.3)
_Lambayeque	29 (19.5)
_Loreto	10 (6.8)
_Junin	9 (6.1)
_La Libertad	6 (4.1)
_Others	37 (25.0)
Not reported	13 (8.8)
Multicountry sample	
_Peru only	135 (91.8)
_Peru and other countries	12 (8.2)
Reported the ethics committee approval	
No	36 (24.5)
_Yes	111 (75.5)
No	36 (24.5)
Reported the ethics approval code	
No	60 (40.8)
_Yes	87 (59.2)
No	60 (40.8)
Total SRQR (in points)*	31 [34 - 38]
Adequate reporting‡	
_No	99 (67.3)

Yes	48 (32.7)

SRQR: Standards for Reporting Qualitative Research.

*Median [p25 - p75]

†For this variable, the percentages add up to more than 100% because some studies were conducted in more than one Peruvian region

‡SRQR score (≥ 37 points)

The highest number of qualitative studies were identified in Lima and Lambayeque, with 75 (50.3%) and 29 (19.5%) studies, respectively, while no studies were identified in Apurímac, Huancavelica, and Tumbes (Fig~~ure~~ 2).

Figure 2. Number of qualitative studies conducted by region.

The SRQR items with the highest level of adherence were the abstract (S2), reported by 95.9% of studies, the statement of study purpose (S4), reported by 95.2%, and the presentation of main findings (S16), reported by 93.9%. On the other hand, only 31.3% of studies adequately reported the title elements (S1). Reporting of the qualitative approach or guiding paradigm (S5) was also limited, with 49.0% of studies meeting this criterion. Similarly, researchers' characteristics and reflexivity (S6) were reported in just 37.4% of studies (Fig~~ure~~ 3).

Figure 3. Fulfillment of the SRQR items among Peruvian qualitative studies (n=147).

This is the Fig~~ure~~ 3~~1~~ legend

Formatted: Indent: Left: 0.02", Line spacing: Double

Formatted: Line spacing: Double

Formatted: Indent: Left: -0.1", Line spacing: Double

Formatted: Indent: Left: -0.1", Line spacing: Double

Formatted: Left, Indent: Left: -0.1", Space After: 0 pt, Line spacing: Double, Pattern: Clear (White)

Formatted: Font: 12 pt, English (United States), Reflection

Formatted: Indent: Left: -0.1", Line spacing: Double

Formatted: Indent: Left: -0.1"

Formatted: Font: Not Bold

Formatted: Indent: Left: -0.1", Line spacing: Double

Formatted: Indent: Left: -0.1"

Formatted: Font: Not Bold

Formatted: Indent: Left: -0.1", Line spacing: Double

SRQR: Standards for Reporting Qualitative Research

S1. Quality of title

S2. Quality of abstract

S3. Introduction. Background and problem description

S4. Introduction. Study purpose and objectives

S5. Methods: Qualitative approach and research paradigm

S6. Methods: Researcher characteristics and reflexivity

S7. Methods: Study context and setting

S8. Methods: Sampling strategy

S9. Methods: Ethical approval and consent

S10. Methods: Data collection procedures

S11. Methods: Data collection instruments

S12. Methods: Participant characteristics

S13. Methods: Data processing and management

S14. Methods: Data analysis process

S15. Methods: Trustworthiness and credibility techniques

S16. Results: Main findings

S17. Results: Supporting evidence for findings

S18. Discussion: Interpretation and contribution to literature

S19. Discussion: Limitations and trustworthiness

S20. Conflicts of interest

S21. Funding sources

Formatted: Indent: Left: -0.1"

In the regression analysis, some study characteristics were associated with a higher prevalence of adequate SRQR reporting (score ≥ 37). Studies published in Spanish showed

a lower frequence of adequate reporting compared with those published in English (PR = 0.12; 95% CI: 0.05–0.32). Studies with eight or more authors (PR = 3.47; 95% CI: 1.81–6.67), studies whose first author was affiliated with a non-Peruvian institution (PR = 0.40; 95% CI: 0.26–0.62), and studies involving international collaboration (PR = 2.35; 95% CI: 1.45–3.82), had a higher prevalence of adequate reporting. In contrast, neither the main population of interest nor publication in a Peruvian journal showed a statistically significant association (Table 2).

Table 2. F~~Associated f~~actors associated with adequate SRQR checklist reporting (n = 147).

Variables	Adequate SRQR reporting		RP (IC 95%)
	No (SRQR score < 37)	Yes (SRQR score ≥ 37)	
Language of the journal			
English	40 (47.6)	44 (52.4)	Ref.
Spanish	59 (93.7)	4 (6.3)	**0.12 (0.05 to 0.32**
Published in a Peruvian journal			
No	87 (66.9)	43 (33.1)	Ref.
Yes	12 (70.6)	5 (29.4)	0.89 (0.41 to 1.94)
Number of authors			
1 to 4	44 (83.0)	9 (17.0)	Ref.

Formatted: Font: Not Bold
Formatted: Indent: Left: -0.1", Line spacing: Double
Formatted: Line spacing: Double
Formatted: Indent: Left: 0.02", Line spacing: Double
Formatted: Indent: Left: 0.02", Line spacing: Double
Formatted: Indent: Left: 0.02", Line spacing: Double
Formatted: Indent: Left: 0.02", Line spacing: Double
Formatted: English (United States)
Formatted: Indent: Left: 0.02", Line spacing: Double
Formatted: Line spacing: Double
Formatted: Indent: Left: 0.02", Line spacing: Double
Formatted: Line spacing: Double
Formatted: Indent: Left: 0.02", Line spacing: Double
Formatted: Indent: Left: 0.02", Line spacing: Double
Formatted: Line spacing: Double

__4 to 7	39 (70.9)	16 (29.1)	1.71 (0.83 to 3.54)
__8 to 36	16 (41.0)	23 (59.0)	**3.47 (1.81 to 6.67)**
Country of the first author's first affiliation			
__Non Peru	18 (42.9)	24 (57.1)	Ref
__Peru	81 (77.1)	24 (22.9)	**0.40 (0.26 to 0.62)**
International collaboration			
__Only authors from Peru	68 (79.1)	18 (20.9)	Ref.
__At least one international author	31 (50.8)	30 (49.2)	**2.35 (1.45 to 3.82)**
Main population of the study			
__Children, adolescents, and young people	16 (76.2)	5 (23.8)	Ref.
__Mothers, fathers, caregivers, and families	25 (83.3)	5 (16.7)	0.70 (0.23 to 2.13)
__Indigenous peoples, individuals from rural communities, and culturally specific groups	12 (57.1)	9 (42.9)	1.80 (0.72 to 4.49)
__Patients, service users, and individuals living with health conditions	14 (56.0)	11 (44.0)	1.85 (0.76 to 4.49)

__Health professionals (clinicians and care personnel)	11 (55.0)	9 (45.0)	1.89 (0.76 to 4.69)
__Policy makers, administrators, program managers, community leaders, stakeholders, and other non-clinical populations	21 (70.0)	9 (30.0)	1.26 (0.49 to 3.24)

SRQR: Standards for Reporting Qualitative Research.

PR: Prevalence ratio. 95% CI: 95% confidence interval. Ref: reference.

Discussion

Main results

A total of 147 qualitative studies were included. , Mmost were of which were published in English, had a median of five authors, and were conducted primarilyprimarily conducted in the capital cityLima and in community settings. First authors were most commonly affiliated with Peruvian institutions, as their primary affiliation (71.4%), and the majority of studies were conducted exclusively by Peruvian authors (58.5%). Moreover, Eethics committee approval was not reported in nearly one quarter24.5% of the studies, and almost half 40.8% did not provide an ethics approval code. The SRQR items with the highest adherence were the abstract (S2), the statement of study purpose (S4), and the presentation of main findings (S16), whereas title elements (S1), the qualitative approach or guiding paradigm (S5), and researchers' characteristics and reflexivity (S6) were less frequently reported. Adequate SRQR reporting was associated with publication in English, a higher number of authors, non-Peruvian first-author affiliation, and international collaboration,

Formatted: Indent: Left: 0.11", Line spacing: Double

Formatted: Line spacing: Double

Formatted: Indent: Left: 0.11", Line spacing: Double

Formatted: Line spacing: Double

Formatted: Indent: Left: -0.1", Line spacing: Double

Formatted: Indent: Left: -0.1"

with no significant associations observed for the study population or publication in Peruvian journals.

Characteristics of the studies

Approximately half of the included studies were conducted in Lima, indicating a pronounced geographical concentration of qualitative research in Peru, consistent with prior evidence of higher scientific output in Lima, the capital of Peru [18,19][18,19]. This finding suggests that qualitative research follows the pattern of centralized scientific activity in Peru. Barriers to research production in other regions may be driven by structural inequities, such as limited access to research training and funding, as well as the concentration of institutions with greater research capacity in Lima [18][18]. Strengthening interregional collaboration, revising funding allocation mechanisms, and prioritizing equitable research capacity development may contribute to a more balanced national research landscape [20][20].

Formatted: English (United States)

Formatted: English (United States)

Formatted: English (United States)

Nearly one quarter of the studies did not report ethical procedures, and over two fifths failed to include an ethics approval code. This finding may reflect two non-mutually exclusive explanations. On the one hand, some studies may not have been reviewed by an ethics committee, which is particularly concerning given that qualitative research typically involves close engagement with participants and may address sensitive topics, thereby requiring ethical oversight to safeguard participant welfare, informed consent, and confidentiality. On the other hand, these results may indicate shortcomings in reporting practices rather than deficiencies in ethical conduct, whereby ethical approval was obtained but not explicitly documented in the published reports.

Well-established ethical and regulatory frameworks, including the Declaration of Helsinki and the International Ethical Guidelines for Health-related Research Involving Humans issued by CIOMS, as well as current Peruvian regulations [21][21], stipulate that all research

Formatted: English (United States)

involving human participants must undergo prior review and approval by a research ethics committee. These provisions aim to safeguard participants' rights, safety, and well-being, and to ensure adherence to fundamental ethical principles such as autonomy, confidentiality, and justice.

Although the first author's primary institutional affiliation was predominantly Peruvian (71.4%), a substantial proportion of the studies involved international collaboration (41.5%). This pattern suggests that, despite local leadership in qualitative research production, international collaboration could play a meaningful role in the development of these studies. Such collaborations may reflect efforts to strengthen methodological rigor, access complementary expertise, and enhance the visibility of research conducted in Peru.

Results of the SRQR checklist

The items most consistently reported included the presence of an abstract containing the key elements of the study (S2), the specification of the study objective in the introduction (S4), and the reporting of results, particularly the description of the main findings and the evidence supporting them (S16 and S17). In contrast, the least frequently addressed elements were the use of techniques to ensure the credibility of the findings (S15), the qualitative approach and research paradigm (S5), the characteristics of the researchers (S6), and the formulation of a title that accurately reflects the nature and focus of the study (S1).

Our findings align with a previous study of qualitative research in pediatric urology, where the abstract and the study objective were also among the most consistently reported items [10]~~[10]~~. Moreover, our results are consistent with a previous research on qualitative studies of malaria in Colombia, which identified credibility techniques and the description of the

methodological paradigm as among the least reported elements– [12][12]. Another study examining qualitative research in community pharmacies likewise documented poor reporting of the title, researcher characteristics, and the nature of the study- [9][9]. However, unlike those studies, which focused on specific populations or thematic areas, our analysis encompasses the qualitative scientific output of an entire region. This convergence suggests that there is a persistent tendency to report items that are commonly required in scientific manuscripts, irrespective of study design (e.g., abstract key aspects, objective, main findings), reflecting adherence to general reporting conventions rather than specific compliance with qualitative reporting guidelines. By contrast, several items that are fundamental to the rigor and transparency of qualitative research were among the least frequently reported.

This pattern indicates that while general reporting standards are largely met, less emphasis is placed on elements that are distinctive to qualitative inquiry and essential for the appraisal of methodological rigor. In this context, strengthening training in qualitative research reporting, encouraging the adoption of reporting guidelines such as SRQR by journals, and ensuring specialized peer review processes are essential to improve reporting quality.

Factors associated with adequate SRQR reporting

Regarding associated factors, articles published in Spanish were associated with lower reporting quality scores. This may be explained by the tendency of non-English-speaking authors to prioritize publication in English due to its greater international visibility [22][22] and citation potential- [23][23]. As a result, manuscripts with stronger methodological rigor and better adherence to reporting standards may be more likely to be submitted to, and accepted by, English-language journals, which could partially explain the observed

Formatted: English (United States)

Formatted: English (United States)

~~association~~. Additionally, studies published in English may more frequently involve international collaborations, which can contribute additional methodological expertise and promote stricter adherence to reporting standards.

Additionally, lower reporting quality was observed in studies whose first author was affiliated with a Peruvian institution, whereas higher reporting quality was found in studies with a larger number of authors and at least one internationally affiliated author. These findings may reflect the potential contribution of i~~ can be jointly interpreted within the context of i~~nternational collaboration to improving reporting standards in qualitative research involving Peruvian populations~~and may reflect the relevance of international collaboration in achieving higher reporting standards in Peruvian qualitative research~~. Previous evidence suggests that international collaboration has a positive impact on research quality~~-~~ [24,25]~~[24,25]~~, particularly in less well-established research systems~~-~~ [26]~~[26]~~, such as Peru.

Formatted: English (United States)

Formatted: English (United States)

Formatted: Not Highlight

Limitations and strengths

This study has some limitations. First, mixed-methods studies were not included, as their methodological quality is assessed using specific appraisal tools, such as the Mixed Methods Appraisal Tool (MMAT), which fall outside the scope of the present evaluation [27]~~[27]~~. Second, the search was restricted to Scopus, excluding regional databases such as SciELO or LILACS due to their limitation in filtering results by affiliation in these repositories. Although Scopus allows the identification of studies with affiliation with Peruvian institutions, this restriction may have omitted qualitative research published in journals with a local scope. Third, the analysis was limited to the 2022–2025 period, to focus on recent studies and reduce potential bias related to the accelerated publication processes during the COVID-19 pandemic~~-~~ [28]~~[28]~~. While this timeframe may have limited

Formatted: English (United States)

Formatted: English (United States)

the inclusion of relevant scientific production, this scope allows for an evaluation of the current state of qualitative research in Peru. Fourth, although the SRQR provides a structured framework for evaluating reporting quality, the interpretation of some items may involve a degree of reviewer subjectivity. AlsoFinally, because the SRQR evaluates reporting quality rather than intrinsic methodological rigor, some studies may have received higher or lower scores based on reporting completeness rather than the strength of their design. Finally, the regression analysis estimated crude associations; therefore, the observed relationships should be interpreted cautiously and should not be interpreted as evidence of causal relationships.

On the other hand, this study also has some strengths. First, flexibility was applied in the interpretation of certain SRQR items to avoid ratings being influenced by editorial decisions rather than the absence of relevant content. For instance, some articles did not present a structured abstract (assessed under item 2) yet contained all expected elements and were therefore awarded full points. Similarly, statements regarding reflexivity and ethical considerations located outside the methods section were accepted as valid. Second, study selection, data extraction, and analysis were conducted independently by pairs of reviewers, with discrepancies resolved by a third or even fourth evaluator, thereby reducing the risk of individual bias and strengthening the reliability of the assessment process. Third, for data extraction, each item was assigned a score, and this score was accompanied by a justification, which allowed for greater discussion among the authors and increased rigor in the assignment of the scores. Finally, the use of a widely recognized instrument such as the SRQR, whose structure allows for the assessment of items applicable across multiple methodological approaches within qualitative research [29][29].

Formatted: English (United States)

Conclusion

The This review identifies key areas for improvement in the reporting of qualitative health studies in the Peruvian population, including techniques to ensure trustworthiness and credibility, methodological approach, researcher reflexivity, title quality, and reporting of ethics approval. Editorial teams could encourage adherence to standardized reporting guidelines and make editorial policies more explicit regarding ethics committee approval and key elements of methodological transparency, thereby supporting improved reporting practices. Future research could investigate whether these challenges persist in other contexts with similar structural and geographical disparities.study included 147 qualitative studies, most of which were published in English, conducted in Lima and community settings, and developed exclusively by Peruvian authors, with first authors primarily affiliated with Peruvian institutions. Nearly one quarter did not report ethics committee approval, and the least frequently reported SRQR items were title elements, the qualitative approach, and researchers' characteristics and reflexivity. Adequate SRQR reporting was associated with publication in English, a higher number of authors, non-Peruvian first-author affiliation, and international collaboration.

Funding statement:

Formatted: Font: 18 pt

This study was self-funded by the authors. YVC's time was funded by the Neuropsychiatry and Behavioral Disorders in First Nations Multidisciplinary Training Program (NEUFIN) grant, NIMH D43MH108169.

Ethical statement:

Formatted: Font: 18 pt

Ethics committee approval was not required because this study involved only a review of previously published literature and did not include human participants.

Conflict -of interest statement:

Formatted: Font: 18 pt

Formatted: Font: 18 pt

The authors declare no conflicts of interest related to this study.

Acknowledgments:

Formatted: Font: 18 pt

None.

Data availability statement:

Formatted: Font: 18 pt

~~The datasets generated and/or analyzed during the present study are available from the corresponding author upon reasonable request.~~The raw data underlying the findings are available in the S4 Table.

References

1. Kuper A, Reeves S, Levinson W. An introduction to reading and appraising qualitative research. *BMJ*. 2008;337:a288. doi:10.1136/bmj.a288

Formatted: Font: 12 pt

Formatted: Justified

2. Huston P, Rowan M. Qualitative studies. Their role in medical research. *Can Fam Physician Med Fam Can*. 1998;44:2453-2458.
3. Renjith V, Yesodharan R, Noronha JA, Ladd E, George A. Qualitative Methods in Health Care Research. *Int J Prev Med*. 2021;12:20. doi:10.4103/ijpvm.IJPVM_321_19
4. Dunt D, McKenzie R. Improving the quality of qualitative studies: do reporting guidelines have a place? *Fam Pract*. 2012;29(4):367-369. doi:10.1093/fampra/cms041
5. Mays N, Pope C. Qualitative research in health care. Assessing quality in qualitative research. *BMJ*. 2000;320(7226):50-52. doi:10.1136/bmj.320.7226.50
6. O’Brien BC, Harris IB, Beckman TJ, Reed DA, Cook DA. Standards for reporting qualitative research: a synthesis of recommendations. *Acad Med J Assoc Am Med Coll*. 2014;89(9):1245-1251. doi:10.1097/ACM.0000000000000388

7. Tong A, Sainsbury P, Craig J. Consolidated criteria for reporting qualitative research (COREQ): a 32-item checklist for interviews and focus groups. *Int J Qual Health Care J Int Soc Qual Health Care*. 2007;19(6):349-357. doi:10.1093/intqhc/mzm042

8. Tong A, Flemming K, McInnes E, Oliver S, Craig J. Enhancing transparency in reporting the synthesis of qualitative research: ENTREQ. *BMC Med Res Methodol*. 2012;12:181. doi:10.1186/1471-2288-12-181

9. Aref HAT, Witry M, Olufemi-Yusuf D, Guirguis LM. Ensuring quality qualitative research reporting in community pharmacy: a systematic literature review. *Int J Pharm Pract*. 2021;29(5):416-427. doi:10.1093/ijpp/riab027

10. McCloskey K, Neuzil K, Basak R, Chan KH. Quality of reporting for qualitative studies in pediatric urology—A scoping review. *J Pediatr Urol*. 2023;19(5):643-651. doi:10.1016/j.jpurol.2023.04.027

11. Silva GS, Fernandes D de RF, Alves CRL. Avaliação da assistência à saúde da criança na Atenção Primária no Brasil: revisão sistemática de métodos e resultados. *Ciênc Saúde Coletiva*. 2020;25:3185-3200. doi:https://doi.org/10.1590/1413-81232020258.27512018

12. Cardona-Arias JA, Salas-Zapata W, Carmona-Fonseca J. Systematic review of qualitative studies about malaria in Colombia. *Heliyon*. 2020;6(5). doi:10.1016/j.heliyon.2020.e03964

13. Angarita FA, Zhang Y, Elmi M, Hong NJL. Older women's experience with breast cancer treatment: A systematic review of qualitative literature. *The Breast*. 2020;54:293-302. doi:10.1016/j.breast.2020.11.009

14. Mendoza-Chuctaya G, Chachaima-Mar JE, Mejia CR, et al. Análisis de producción, impacto y redes de colaboración en investigaciones científicas en Scopus en Perú de 2000 a 2019. *Medwave*. 2021;21(2). doi:10.5867/medwave.2021.02.8121

15. Carcausto-Calla WH, Morales-Quispe J. Investigaciones cualitativas en salud publicadas en revistas biomédicas peruanas. *An Fac Med*. 2018;79(2):144-148. doi:10.15381/anales.v79i2.14941

16. Watson A, Jackson D. The Importance of Analytic Integrity and Reporting Guidelines in Reporting Qualitative Research. *J Adv Nurs*. 2025;81(7):3425-3427. doi:10.1111/jan.16617

17. Tricco AC, Lillie E, Zarin W, et al. PRISMA Extension for Scoping Reviews (PRISMA-ScR): Checklist and Explanation. *Ann Intern Med*. 2018;169(7):467-473. doi:10.7326/M18-0850

18. Arcila-Diaz J, Delgado-Caramutti J, Millones-Gómez PA, Figueroa-Quiñones J, Valencia-Arias A. Research trends in Peruvian universities: proposal for a research agenda with a bibliometric approach. *Scientometrics*. 2025;130(1):489-514. doi:10.1007/s11192-024-05211-z

19. Vásquez-Uriarte K, Roque-Henriquez JC, Angulo-Bazán Y, Ninatanta Ortiz JA. Análisis bibliométrico de la producción científica peruana sobre la COVID-19. *Rev*

Peru Med Exp Salud Pública. 2021;38:224-231. doi:https://doi.org/10.17843/rpmesp.2021.382.7470

20. Choquez-Millan MF, Lechtape CL, Löhr K, Schröter B, Graef F. Uncovering power asymmetries in North-South research collaborations – An example from sustainability research in Tanzania. *Futures*. 2024;156:103316. doi:10.1016/j.futures.2023.103316

21. Resolución Ministerial N.° 233-2020-MINSA. Accessed January 8, 2026. https://www.gob.pe/institucion/minsa/normas-legales/541139-233-2020-minsa

22. Stockemer D, Wigginton MJ. Publishing in English or another language: An inclusive study of scholar's language publication preferences in the natural, social and interdisciplinary sciences. *Scientometrics*. 2019;118(2):645-652. doi:10.1007/s11192-018-2987-0

23. Di Bitetti MS, Ferreras JA. Publish (in English) or perish: The effect on citation rate of using languages other than English in scientific publications. *Ambio*. 2017;46(1):121-127. doi:10.1007/s13280-016-0820-7

24. Kohus Z, Demeter M, Szigeti GP, Kun L, Lukács E, Czakó K. The Influence of International Collaboration on the Scientific Impact in V4 Countries. *Publications*. 2022;10(4):1-13. doi: 10.3390/publications10040035

25. Wang J, Frietsch R, Neuhäusler P, Hooi R. International collaboration leading to high citations: Global impact or home country effect? *J Informetr*. 2024;18(4):101565. doi:10.1016/j.joi.2024.101565

26. Farias Borges LF, Fischer B, Mazoni A, Bascur JP. The complex interplay between international collaboration and scientific impact: evidence from the Scopus database. *Cogent Educ*. 2025;12(1):2563708. doi:10.1080/2331186X.2025.2563708

27. Hong QN, Fàbregues S, Bartlett G, et al. The Mixed Methods Appraisal Tool (MMAT) version 2018 for information professionals and researchers. *Educ Inf*. 2018;34(4):285-291. doi:10.3233/EFI-180221

28. McDermott KT, Perry M, Linden W, Croft R, Wolff R, Kleijnen J. The quality of COVID-19 systematic reviews during the coronavirus 2019 pandemic: an exploratory comparison. *Syst Rev*. 2024;13(1):126. doi:10.1186/s13643-024-02552-x

29. Dossett LA, Kaji AH, Cochran A. SRQR and COREQ Reporting Guidelines for Qualitative Studies. *JAMA Surg*. 2021;156(9):875-876. doi:10.1001/jamasurg.2021.0525

Supporting information

S1 Table. Preferred Reporting Items for Systematic reviews and Meta-Analyses extension for Scoping Reviews (PRISMA-ScR) checklist.

Formatted: Indent: Left: -0.1"

S2 Table. Characteristics of the included studies (n=147)

S3 Table. Table of excluded studies (n=55)

S4 Table. Extracted dataset

Formatted: Normal, Indent: Left: -0.1", Line spacing: Double

Formatted: Normal, Indent: Left: -0.1"

Formatted: Indent: Left: -0.1", Line spacing: Double

Dear editor,
Many thanks for your response to the submission of our paper

We sincerely thank you and the reviewers for their thoughtful and constructive comments regarding the submission of our manuscript. We have carefully revised the manuscript in response to all reviewers' feedback and have addressed each suggestion in detail. We trust that the revisions undertaken have enhanced the clarity and overall quality of the manuscript. Our point-by-point responses to each comment are provided below.

Rubí Paredes-Angeles
rubiparedesa@gmail.com
Corresponding author

Please respond to the following query regarding the map contained in Figure 2. Where did the authors obtain the maps, basemaps, shapefiles, map data, etc in this Figure? Please can you provide us with a direct link to the data used and a link to its data license. What software was used to make these maps?

a. The map in Figure 2 was created using publicly available geographic data accessed through the mapsPeru package in R software, which is free and open-source software (https://www.nihlibrary.nih.gov/resources/tools/r-and-rstudio). The mapsPERU package (https://github.com/cran/mapsPeru) is distributed under the GNU General Public License (GPL-3): https://www.gnu.org/licenses/gpl-3.0.html. No proprietary basemaps or restricted data sources were used in the creation of the figure.

   Regarding licensing, the data are distributed as open-access geographic data for public use. Additionally, the package repository can be accessed here:
   https://github.com/cran/mapsPeru

   Previous studies have also used this package:
   https://doi.org/10.1186/s12960-023-00805-9
   https://doi.org/10.1186/s13690-026-01872-9

b. Software used to make the maps:
   The map was created using the R statistical software, specifically with the mapsPeru package. In the revised version, we have clearly specified this information in the Methods section, as indicated in the reviewers' and editor's comments regarding the map.

Review Comments to the Author

Reviewer #1
Comment: 1. The manuscript presents a technically sound piece of scientific research. The study's data support the conclusion. However, the following points need to be considered:
Introduction section, paragraph 4, page 6
The authors mentioned that health research publications have increased in Peru. They cited the source of that information. Although it is okay, they could have spiced up the argument by stating the magnitude of the increase.

Reply: In response, we have strengthened the argument in this section by specifying the magnitude of the increase in health research in Peru. The following sentence was added: "In Peru, scientific research output has increased, with an estimated annual growth rate of 13.6%, with 70.2% corresponds to health sciences [14]"

Comment: The authors have claimed that "to date, no studies have specifically examined qualitative health research or assessed the quality of its reporting." The argument lacks clarity and evidence. Can they provide evidence that their study is of the first kind in Peru?
Study eligibility, paragraph 2, page 7

Reply: We have revised the statement to avoid an overgeneralized claim and improve clarity. The paragraph now reads: "A previous study examined the scientific production of qualitative research in Peru; however, it did not assess the reporting quality of these studies [15]." This correction acknowledges prior evidence, incorporates the relevant reference [15], and recognizes the limitation regarding the absence of reporting quality assessment.

Comment: "Only studies in which at least 25% of participants were based in Peru were considered." Why? Can this statement be supported with the reason for including studies in which at least 25% of participants were based in Peru?

Reply: We added the following sentence in the second paragraph of the "Study eligibility" section: "Only studies in which at least 25% of participants were based in Peru were considered to ensure meaningful representation of the Peruvian population while allowing the inclusion of international studies."

Comment: Conclusion, pages 22 and 23
Conclusion lacks recommendations from the study's findings.

Reply: The conclusion has been revised as follows: "This review identifies key areas for improvement in the reporting of qualitative health studies in the Peruvian population, including techniques to ensure trustworthiness and credibility, methodological approach, researcher reflexivity, title quality, and reporting of ethics approval. Research remains heavily concentrated in the capital, and international collaboration appears to enhance reporting

quality. To strengthen transparency and methodological rigor, journals and reviewers should adopt standardized reporting tools such as SRQR or COREQ, and interregional as well as international collaboration should be actively encouraged. Future research could investigate whether these challenges persist in other contexts with similar structural and geographical disparities."

Comment: 2. The authors have performed statistical analysis appropriately and rigorously. They have used simple statistical analyses by reporting prevalence ratios and frequencies. However, they should consider revising the presentation of the frequencies in Table 1. It is more appearing to arrange the frequencies of the variables in a table, either from highest to lowest percentages or vice versa. Table 1 is on pages 13 and 14.

Reply: The order of variables in Table 1 has been revised to present them in descending order according to their percentages.

Comment: 3. The authors did not make the data underlying the findings in their manuscript fully available. However, they state that the data will be available upon reasonable request from the corresponding author.

Reply:

Comment: 4. The manuscript is presented in an intelligible fashion and written in standard English. However, in the discussion section (main results, page 17), the paragraph is redundant to what has already been presented in the results section. Again, on pages 7 and 8, the Spanish was mentioned twice. Additionally, using the word "to date" (page 6) will be vague if the paper is read in 2 or more years from now. Can authors replace "to date" with other word(s)?

Reply: The "Main Results" paragraph in the Discussion section has been revised to avoid redundancy with the Results section. Specifically, numerical values were removed and replaced with approximate descriptive expressions (e.g., "nearly one quarter" instead of "24.5%") to improve readability and reduce repetition. Regarding the language variable (pages 7–8), the wording was clarified to avoid repetition. It has been revised as follows: "language of the study (English, Spanish, or both English and Spanish)". Finally, the expression "to date" has been removed and replaced with more precise wording.

Reviewer #2: Overall comment:
The review rationale is relevant as it aims to contribute to improving future qualitative research in health. However, several points are to be considered to enhance the manuscript's rigor, transparency and relevance. They are detailed below in major and minor comments.

Comment: Introduction. 1/ p.6: The study aim is stated as follows: "this study aimed to describe the methodological characteristics and assess the reporting quality of qualitative studies published by Peruvian authors." (p.6). In the eligibility criteria,

authors stated: "Only studies in which at least 25% of participants were based in Peru were considered." (p.11).
This is a bit confusing. Authors should specify clearly if it's about qualitative studies published by Peruvian authors or qualitative studies conducted in Peru.

Reply: To clarify this point, we revised the final sentence of the Introduction (study aim) as follows: "this study aimed to describe the methodological characteristics and assess the reporting quality of qualitative studies conducted in populations residing in Peru and involving at least one author affiliated with a Peruvian institution." This revision clarifies that the study includes both research conducted in Peruvian populations and studies involving at least one author with a Peruvian institutional affiliation. This criterion is also consistently specified in the "Study eligibility" section.

Comment: Methods. 1/ p.6: Why focusing especially on 2022-2025 period to get recent studies? Authors have provided an explanation in Discussion. However, are the studies published prior 2022-2025 no more relevant or used to inform health practice and policy?

Reply: Our decision to restrict the analysis to studies published between 2022 and 2025 was not based on the assumption that earlier studies are no longer relevant. Rather, it was a deliberate methodological choice aligned with the objective of this scoping review, which was to describe the current state of reporting practices in qualitative health research conducted by Peruvian authors.

Focusing on recent years allows for a more accurate assessment of contemporary reporting standards, particularly in the context of the increasing dissemination and adoption of reporting guidelines such as SRQR. In addition, this timeframe reduces potential distortions associated with atypical publication patterns observed during the COVID-19 pandemic period.

Comment: 2/ p.6: Authors claim to have conducted a systematic search. Yet they only searched Scopus without a justification. Why did they search only one database and how inclusive/comprehensive do they think their review is? Did they consider other sources such as references lists of identified articles?

Reply: The decision to use Scopus as the sole database was based on its broad coverage and its capacity to filter results by authors' institutional affiliation (i.e., affiliation with Peru), which was a key eligibility criterion in our review. To our knowledge, other commonly used databases such as PubMed, SciELO, or LILACS do not allow for such precise and reproducible filtering by affiliation, which limited their suitability for our specific objective. Regarding additional search strategies, we did not perform manual searches of reference lists. Given the scope of this review and the structured search strategy applied, we prioritized a reproducible and database-driven approach.

Therefore, our findings should be interpreted as representative of the qualitative health research indexed in Scopus rather than the entirety of qualitative research conducted in Peru. These limitations have been explicitly acknowledged in the revised manuscript.

Comment: 3/ p.7: How relevant do you think it would be to group studies by health disciplines? Researchers usually work in specific health disciplines and your findings would be more insightful if you provide them with discipline-based evidence.

Reply: Although we initially considered it, many of the included studies addressed interdisciplinary topics or involved overlapping populations and settings, which posed difficulties for a coherent and mutually exclusive classification into distinct health disciplines. Nevertheless, we recognize the potential value of this approach. In our study, we partially addressed this dimension by categorizing studies according to their main population and research setting, which may serve as proxies for disciplinary focus.

Comment: 4/ p.8: Authors used the highest tertile to define "adequate SRQR reporting". Is this threshold a standard in the field or just authors' choice? This should be clarified.

Reply: The use of the highest tertile to define "adequate SRQR reporting" was a methodological decision made by the authors rather than a standardized or universally established threshold in the field.

Currently, there is no cut-off point for categorizing reporting quality based on SRQR scores. In this context, we opted for a data-driven approach by classifying studies according to the distribution of scores within our sample. Defining "adequate reporting" as the highest tertile allowed us to identify studies with comparatively better adherence to SRQR items while maintaining sufficient variability for the analysis of associated factors. This approach has been used in previous methodological studies, where relative thresholds (e.g., tertiles or quartiles) are applied in the absence of validated cut-offs. However, we acknowledge that such categorization is somewhat arbitrary and should be interpreted cautiously.

We have added in Methods: "Given the absence of established or validated cut-off points for SRQR scores, this data-driven approach was used to identify studies with comparatively better reporting quality."

We have added in Discussion: "Fourth, "adequate SRQR reporting" was defined using the highest tertile of the score distribution due to the absence of standardized thresholds. This data-driven approach should be interpreted with caution."

Comment: 5/ p.8-11: This SRQR-based study assessment remains somehow subjective since the appreciation may vary by assessor. I believe that if another research group had to reproduce this review, they may give different scores to each study for each item. Are

authors aware of this subjectivity and variability concern? What strategy did they implement to mitigate this limitation?

Reply: We acknowledge that SRQR-based assessments may involve a degree of subjectivity, as judgments can vary across assessors. To mitigate this, we implemented several strategies to enhance consistency and reproducibility. First, our data extraction matrix included two components for the SRQR score: one for the assigned score and another to document the rationale for that score, including verbatim excerpts from the reviewed articles to justify each rating. Second, each pair of investigators independently extracted study characteristics and SRQR items. They then met to compare each cell and reach consensus on the score assigned to each item, resulting in a final extraction. Discrepancies that could not be resolved were discussed during weekly team meetings until agreement was achieved. Additionally, prior to formal data extraction, the team conducted calibration exercises to ensure a shared understanding of SRQR criteria and consistent application across reviewers.

To clarify this, we have added in Methods section: "The data extraction matrix included two components for the SRQR score: one for the assigned score and another to document the rationale, including verbatim excerpts from the reviewed articles to justify each rating. Each pair of investigators independently extracted study characteristics and SRQR items, and then met to compare each cell and reach consensus, resulting in a final extraction. Discrepancies that could not be resolved were discussed during weekly team meetings until agreement was achieved."

Comment: 6/ It is widely recognized that study reporting quality depends on research expertise and vary both by health discipline (dentistry vs. public health) and by authors' affiliation type (clinical vs. research institution affiliation). Since the information on health discipline was available, why did authors not consider it as a descriptive characteristic and subsequently test its association with adequate reporting categories?

Reply: We thank the reviewer for this comment. While we agree that reporting quality may vary across health disciplines, we did not include this variable due to challenges in consistently classifying studies into mutually exclusive categories, particularly for multidisciplinary research. As we mentioned above, this could introduce misclassification and affect the interpretability of the findings.

Results
1/ Please format all tables according to the journal's guidelines.

Reply: All tables have been revised and reformatted to comply with the journal's guidelines.

2/ Figure 1: authors stated that 287 studies were excluded by title and abstract. Can they briefly specify the reasons both in the figure and in the descriptive text?

Reply: The reasons for exclusion at the title and abstract screening stage have now been specified in Figure 1 and clearly described in the corresponding section of the text. Specifically, the following sentence was added to the Results section: "Records were excluded at this stage because they did not meet the eligibility criteria, including studies that were not qualitative, were not related to health sciences, did not involve populations residing in Peru, or corresponded to non-original publications."

3/ Figure 2: mandatory elements of a geographical map, such as direction (North) and gradient (scale), are missing. Please consider adding them.

Reply: Figure 2 has been revised to include the mandatory cartographic elements, including the North direction and scale, in accordance with standard conventions**.**

4/ Adequate reporting is reported in Table 1 but not commented in the text. Please consider adding an in-text comment and a table footnote recalling how it was defined.

Reply: An in-text comment has been added to the Results section, and a footnote has been included in Table 1 to clarify the definition of adequate reporting. Specifically, the following sentence was incorporated into the text: "Adequate SRQR reporting was observed in 32.7% of the included studies."

5/ p.15: It seems counterintuitive to interpret an association by the reference group as authors did for Table 2 regarding non-Peruvian vs. Peruvian first author's affiliation (PR = 0.40, 95% CI: 0.26–0.62). If authors wish to keep this interpretation, I suggest them to assign the reference group to Peruvian authors.

Reply:

Discussion

1/ Considering you research rationale; how do authors interpret and explain a study with one Peruvian author (for instance at sixth position) and many foreigner co-authors? Is it treated as a Peru-affiliated study at the same level as the one exclusively authored by Peru-based researchers?

Reply:

2/ p.17: Please, authors should remove proportions from the Main Results recall in the Discussion section. Instead, they could use descriptive terms such as half of studies, three-quarter, one-third, etc. which are more appealing as summary.

Reply:

3/ p.19-20: Authors compare their findings with the existing literature. They mainly focused on similar findings, while less is said about divergence between their results and the existing

evidence. Moreover, less is said about the studies of comparison besides thematic areas. They should consider improving this.

Reply:

4/ p.20: Authors should consider revising the paragraph #1 of the subsection “Factors associated with…” First, delete “…which could partially explain the observed association” because it is repetitive with the second sentence starting: “This may be explained by the tendency…”. Second, another explanation to consider may be the fact that English studies are often co-authored by international researchers, requiring English as the collaborative language and bringing additional level of rigor and expertise, as acknowledged in the subsequent paragraph.

Reply:

5/ p.21-22: Two major limitations need to be considered. First, there is a rater subjectivity in using SRQR checklist that should not be dismissed, yet not discussed by authors. This can be observed in the Strengths paragraph where authors praise their “flexibility” in rating studies, which was even not mentioned in Methods. Second, regression analysis estimated crude associations that should be explicitly acknowledged in the discussion alongside their implications for both these findings and future research.

Reply:

Conclusion
1/ p.22: The conclusion reads more like results recall than overall summary and practical recommendations for qualitative health research stakeholders. Authors should consider complying with the PRISMA-ScR recommendation relative to conclusion: “Provide a general interpretation of the results with respect to the review questions and objectives, as well as potential implications and/or next steps.”

Reply:

* MINOR COMMENTS

1/ Please, revise the referencing format throughout the text according to the journal’s guidelines.
2/ p.6: I suggest to reformulate concisely the following text: From “A detailed study protocol was developed prior to study initiation and made publicly available. The protocol of this review is available at https://osf.io/jes6v/files/3rn52.” to “A detailed study protocol was developed prior to study initiation and made publicly available (https://osf.io/jes6v/files/3rn52)”

Reply:

3/ p.7: Please, provide an internet link to Rayyan software to align with proper software referencing (city, company, website). Same for Stata software and revise as follows: "…Stata software, version 19.0." (p.11)

Reply:

4/ p.7: Please revise: "…with the collaboration of other authors." or "…with the collaboration of another author."

Reply:

5/ A lot of paragraphs are too short, with only 1 to 3 sentences within a subsection. Please, revise to make paragraphs more compact.

Reply:

6/ p.11: Please, use appropriate terminology: replace "numeric variables" by "quantitative variables"

Reply:

7/ p.11: Please, add statistical threshold: "…crude prevalence ratios (PR) with 95% confidence intervals (CI) were estimated…"

Reply:

8/ Please, reformulate Table 2 title as follows: "Factors associated with adequate…"

Reply:

9/ I am just curious to know how long it took to check 147 studies against a 21-item checklist.

Reply:

Journal Requirements:

When submitting your revision, we need you to address these additional requirements.

1. Please ensure that your manuscript meets PLOS ONE's style requirements, including those for file naming. The PLOS ONE style templates can be found at

https://journals.plos.org/plosone/s/file?id=wjVg/PLOSOne_formatting_sample_main_body.pdf and

https://journals.plos.org/plosone/s/file?id=ba62/PLOSOne_formatting_sample_title_authors_affiliations.pdf

2. Thank you for stating the following in the Funding Section of your manuscript:

"This study was self-funded by the authors. YVC's time was funded by the Neuropsychiatry and Behavioral Disorders in First Nations Multidisciplinary Training Program (NEUFIN) grant, NIMH D43MH108169."

We note that you have provided funding information that is not currently declared in your Funding Statement. However, funding information should not appear in the Acknowledgments section or other areas of your manuscript. We will only publish funding information present in the Funding Statement section of the online submission form.

Please remove any funding-related text from the manuscript and let us know how you would like to update your Funding Statement. Currently, your Funding Statement reads as follows:

"The author(s) received no specific funding for this work."

Please include your amended statements within your cover letter; we will change the online submission form on your behalf.

3. Your ethics statement should only appear in the Methods section of your manuscript. If your ethics statement is written in any section besides the Methods, please move it to the Methods section and delete it from any other section. Please ensure that your ethics statement is included in your manuscript, as the ethics statement entered into the online submission form will not be published alongside your manuscript.

4. We note that Figure 2 in your submission contain map images which may be copyrighted. All PLOS content is published under the Creative Commons Attribution License (CC BY 4.0), which means that the manuscript, images, and Supporting Information files will be freely available online, and any third party is permitted to access, download, copy, distribute, and use these materials in any way, even commercially, with proper attribution. For these reasons, we cannot publish previously copyrighted maps or satellite images created using proprietary data, such as Google software (Google Maps, Street View, and Earth). For more information, see our copyright guidelines: http://journals.plos.org/plosone/s/licenses-and-copyright.

We require you to either (1) present written permission from the copyright holder to publish these figures specifically under the CC BY 4.0 license, or (2) remove the figures from your submission:

1. You may seek permission from the original copyright holder of Figure 2 to publish the content specifically under the CC BY 4.0 license. We recommend that you contact the original copyright holder with the Content Permission Form (http://journals.plos.org/plosone/s/file?id=7c09/content-permission-form.pdf) and the following text: “I request permission for the open-access journal PLOS ONE to publish XXX under the Creative Commons Attribution License (CCAL) CC BY 4.0 (http://creativecommons.org/licenses/by/4.0/). Please be aware that this license allows unrestricted use and distribution, even commercially, by third parties. Please reply and provide explicit written permission to publish XXX under a CC BY license and complete the attached form.” Please upload the completed Content Permission Form or other proof of granted permissions as an "Other" file with your submission. In the figure caption of the copyrighted figure, please include the following text: “Reprinted from [ref] under a CC BY license, with permission from [name of publisher], original copyright [original copyright year].”

2. If you are unable to obtain permission from the original copyright holder to publish these figures under the CC BY 4.0 license or if the copyright holder’s requirements are incompatible with the CC BY 4.0 license, please either i) remove the figure or ii) supply a replacement figure that complies with the CC BY 4.0 license. Please check copyright information on all replacement figures and update the figure caption with source information. If applicable, please specify in the figure caption text when a figure is similar but not identical to the original image and is therefore for illustrative purposes only.

Reply: We would like to clarify that Figure 2 was entirely generated by the authors using R (packages “sf” and “mapsPERU”). The map is based on vector-based administrative boundary data of Peru obtained from publicly available sources. No proprietary basemaps (such as Google Maps, Google Earth, or similar services) were used at any stage of the figure creation process. Therefore, the figure does not contain copyrighted map tiles or satellite imagery and is fully compatible with the Creative Commons CC BY 4.0 license required by PLOS ONE.

We have included in Methods: “Spatial data processing and visualization were performed in R (version 4.2.2; R Foundation for Statistical Computing, Vienna, Austria). Administrative boundary data for Peru were obtained from the “mapsPERU” package (version 2.0.0), which provides publicly available vector-based geospatial data. Spatial data were managed using the “sf” package (version 1.0.16). Data manipulation was conducted using “dplyr” package (version 1.1.4) and “ggplot2” package (version 3.5.1). No proprietary basemaps were used.”

5. If the reviewer comments include a recommendation to cite specific previously published works, please review and evaluate these publications to determine whether they are relevant and should be cited. There is no requirement to cite these works unless the editor has indicated otherwise.

6. Please review your reference list to ensure that it is complete and correct. If you have cited papers that have been retracted, please include the rationale for doing so in the manuscript text, or remove these references and replace them with relevant current references. Any changes to the reference list should be mentioned in the rebuttal letter that accompanies your revised manuscript. If you need to cite a retracted article, indicate the article's retracted status in the References list and also include a citation and full reference for the retraction notice.
