# Supplementary material for: Reporting quality of qualitative health studies published by Peruvian authors: A scoping review
Source: PLoS One. 2026 Jun 23;21(6):e0351494. doi: 10.1371/journal.pone.0351494 (PMC13289893; doi:10.1371/journal.pone.0351494)
Supplement: Peer review history

# Peer review: Decision Letter 0

**DOI:** 10.1371/journal.pone.0351494.r001

**Authors:** Alfredo Fort

26 Feb 2026

PONE-D-26-05648

Reporting quality of qualitative health studies published by Peruvian authors: a scoping review

PLOS One

Dear Dr. Paredes-Angeles,

Thank you for submitting your manuscript to PLOS ONE. After careful consideration, we feel that it has merit but does not fully meet PLOS ONE’s publication criteria as it currently stands. Therefore, we invite you to submit a revised version of the manuscript that addresses the points raised during the review process.

Please submit your revised manuscript by Apr 12 2026 11:59PM. If you will need more time than this to complete your revisions, please reply to this message or contact the journal office at plosone@plos.org. When you're ready to submit your revision, log on to https://www.editorialmanager.com/pone/ and select the 'Submissions Needing Revision' folder to locate your manuscript file.

Please include the following items when submitting your revised manuscript:

- A letter that responds to each point raised by the academic editor and reviewer(s). You should upload this letter as a separate file labeled 'Response to Reviewers'.
- A marked-up copy of your manuscript that highlights changes made to the original version. You should upload this as a separate file labeled 'Revised Manuscript with Track Changes'.
- An unmarked version of your revised paper without tracked changes. You should upload this as a separate file labeled 'Manuscript'.

-->If you would like to make changes to your financial disclosure, please include your updated statement in your cover letter. Guidelines for resubmitting your figure files are available below the reviewer comments at the end of this letter.

If applicable, we recommend that you deposit your laboratory protocols in protocols.io to enhance the reproducibility of your results. Protocols.io assigns your protocol its own identifier (DOI) so that it can be cited independently in the future. For instructions see: https://journals.plos.org/plosone/s/submission-guidelines#loc-laboratory-protocols. Additionally, PLOS ONE offers an option for publishing peer-reviewed Lab Protocol articles, which describe protocols hosted on protocols.io. Read more information on sharing protocols at https://plos.org/protocols?utm_medium=editorial-email&utm_source=authorletters&utm_campaign=protocols.

We look forward to receiving your revised manuscript.

Kind regards,

Alfredo Luis Fort, M.D., M.Sc., Ph.D.

Academic Editor

PLOS One

**Journal Requirements:**

When submitting your revision, we need you to address these additional requirements.

1. Please ensure that your manuscript meets PLOS ONE's style requirements, including those for file naming. The PLOS ONE style templates can be found at

https://journals.plos.org/plosone/s/file?id=wjVg/PLOSOne_formatting_sample_main_body.pdf and

https://journals.plos.org/plosone/s/file?id=ba62/PLOSOne_formatting_sample_title_authors_affiliations.pdf

2. Thank you for stating the following in the Funding Section of your manuscript:

“This study was self-funded by the authors. YVC's time was funded by the Neuropsychiatry and Behavioral Disorders in First Nations Multidisciplinary Training Program (NEUFIN) grant, NIMH D43MH108169.”

We note that you have provided funding information that is not currently declared in your Funding Statement. However, funding information should not appear in the Acknowledgments section or other areas of your manuscript. We will only publish funding information present in the Funding Statement section of the online submission form.

Please remove any funding-related text from the manuscript and let us know how you would like to update your Funding Statement. Currently, your Funding Statement reads as follows:

“The author(s) received no specific funding for this work.”

Please include your amended statements within your cover letter; we will change the online submission form on your behalf.

3. Your ethics statement should only appear in the Methods section of your manuscript. If your ethics statement is written in any section besides the Methods, please move it to the Methods section and delete it from any other section. Please ensure that your ethics statement is included in your manuscript, as the ethics statement entered into the online submission form will not be published alongside your manuscript.

4. We note that Figure 2 in your submission contain map images which may be copyrighted. All PLOS content is published under the Creative Commons Attribution License (CC BY 4.0), which means that the manuscript, images, and Supporting Information files will be freely available online, and any third party is permitted to access, download, copy, distribute, and use these materials in any way, even commercially, with proper attribution. For these reasons, we cannot publish previously copyrighted maps or satellite images created using proprietary data, such as Google software (Google Maps, Street View, and Earth). For more information, see our copyright guidelines: http://journals.plos.org/plosone/s/licenses-and-copyright.

We require you to either (1) present written permission from the copyright holder to publish these figures specifically under the CC BY 4.0 license, or (2) remove the figures from your submission:

1. You may seek permission from the original copyright holder of Figure 2 to publish the content specifically under the CC BY 4.0 license.

We recommend that you contact the original copyright holder with the Content Permission Form (http://journals.plos.org/plosone/s/file?id=7c09/content-permission-form.pdf) and the following text:

“I request permission for the open-access journal PLOS ONE to publish XXX under the Creative Commons Attribution License (CCAL) CC BY 4.0 (http://creativecommons.org/licenses/by/4.0/). Please be aware that this license allows unrestricted use and distribution, even commercially, by third parties. Please reply and provide explicit written permission to publish XXX under a CC BY license and complete the attached form.”

Please upload the completed Content Permission Form or other proof of granted permissions as an "Other" file with your submission.

In the figure caption of the copyrighted figure, please include the following text: “Reprinted from [ref] under a CC BY license, with permission from [name of publisher], original copyright [original copyright year].”

2. If you are unable to obtain permission from the original copyright holder to publish these figures under the CC BY 4.0 license or if the copyright holder’s requirements are incompatible with the CC BY 4.0 license, please either i) remove the figure or ii) supply a replacement figure that complies with the CC BY 4.0 license. Please check copyright information on all replacement figures and update the figure caption with source information. If applicable, please specify in the figure caption text when a figure is similar but not identical to the original image and is therefore for illustrative purposes only.

The following resources for replacing copyrighted map figures may be helpful:

USGS National Map Viewer (public domain): http://viewer.nationalmap.gov/viewer/

The Gateway to Astronaut Photography of Earth (public domain): http://eol.jsc.nasa.gov/sseop/clickmap/

Maps at the CIA (public domain): https://www.cia.gov/library/publications/the-world-factbook/index.html and https://www.cia.gov/library/publications/cia-maps-publications/index.html

NASA Earth Observatory (public domain): http://earthobservatory.nasa.gov/

Landsat: http://landsat.visibleearth.nasa.gov/

USGS EROS (Earth Resources Observatory and Science (EROS) Center) (public domain): http://eros.usgs.gov/#

Natural Earth (public domain): http://www.naturalearthdata.com/

5. If the reviewer comments include a recommendation to cite specific previously published works, please review and evaluate these publications to determine whether they are relevant and should be cited. There is no requirement to cite these works unless the editor has indicated otherwise.

6. Please review your reference list to ensure that it is complete and correct. If you have cited papers that have been retracted, please include the rationale for doing so in the manuscript text, or remove these references and replace them with relevant current references. Any changes to the reference list should be mentioned in the rebuttal letter that accompanies your revised manuscript. If you need to cite a retracted article, indicate the article’s retracted status in the References list and also include a citation and full reference for the retraction notice.

**Additional Editor Comments:**

The authors have designed and presented an important study to the research field. In general, they have used appropriate methodology and description of results. However, there are a few areas in the manuscript that required better explanations and corrections of a few errors. Please find the suggestions by the reviewers, plus a few additional comments I have made in the attached file. Please proceed to improve your article to get it ready for publication. Thank you.

[Note: HTML markup is below. Please do not edit.]

Reviewers' comments:

Reviewer's Responses to Questions

**Comments to the Author**

1. Is the manuscript technically sound, and do the data support the conclusions?

The manuscript must describe a technically sound piece of scientific research with data that supports the conclusions. Experiments must have been conducted rigorously, with appropriate controls, replication, and sample sizes. The conclusions must be drawn appropriately based on the data presented.

Reviewer #1: Yes

Reviewer #2: Yes

**********

2. Has the statistical analysis been performed appropriately and rigorously?

Reviewer #1: Yes

Reviewer #2: Yes

**********

3. Have the authors made all data underlying the findings in their manuscript fully available?

The PLOS Data policy requires authors to make all data underlying the findings described in their manuscript fully available without restriction, with rare exception (please refer to the Data Availability Statement in the manuscript PDF file). The data should be provided as part of the manuscript or its supporting information, or deposited to a public repository. For example, in addition to summary statistics, the data points behind means, medians and variance measures should be available. If there are restrictions on publicly sharing data—e.g. participant privacy or use of data from a third party—those must be specified.

Reviewer #1: No

Reviewer #2: Yes

**********

4. Is the manuscript presented in an intelligible fashion and written in standard English?

PLOS ONE does not copyedit accepted manuscripts, so the language in submitted articles must be clear, correct, and unambiguous. Any typographical or grammatical errors should be corrected at revision, so please note any specific errors here.

Reviewer #1: Yes

Reviewer #2: Yes

**********

5. Review Comments to the Author

Please use the space provided to explain your answers to the questions above. You may also include additional comments for the author, including concerns about dual publication, research ethics, or publication ethics. (Please upload your review as an attachment if it exceeds 20,000 characters)

Reviewer #1: 1. The manuscript presents a technically sound piece of scientific research. The study’s data support the conclusion. However, the following points need to be considered:

Introduction section, paragraph 4, page 6

• The authors mentioned that health research publications have increased in Peru. They cited the source of that information. Although it is okay, they could have spiced up the argument by stating the magnitude of the increase.

• The authors have claimed that “to date, no studies have specifically examined qualitative health research or assessed the quality of its reporting.” The argument lacks clarity and evidence. Can they provide evidence that their study is of the first kind in Peru?

Study eligibility, paragraph 2, page 7

• “Only studies in which at least 25% of participants were based in Peru were considered.” Why? Can this statement be supported with the reason for including studies in which at least 25% of participants were based in Peru?

Conclusion, pages 22 and 23

• Conclusion lacks recommendations from the study’s findings.

2. The authors have performed statistical analysis appropriately and rigorously. They have used simple statistical analyses by reporting prevalence ratios and frequencies. However, they should consider revising the presentation of the frequencies in Table 1. It is more appearing to arrange the frequencies of the variables in a table, either from highest to lowest percentages or vice versa. Table 1 is on pages 13 and 14.

3. The authors did not make the data underlying the findings in their manuscript fully available. However, they state that the data will be available upon reasonable request from the corresponding author.

4. The manuscript is presented in an intelligible fashion and written in standard English. However, in the discussion section (main results, page 17), the paragraph is redundant to what has already been presented in the results section. Again, on pages 7 and 8, the Spanish was mentioned twice. Additionally, using the word “to date” (page 6) will be vague if the paper is read in 2 or more years from now. Can authors replace “to date” with other word(s)?

Reviewer #2: Overall comment:

The review rationale is relevant as it aims to contribute to improving future qualitative research in health. However, several points are to be considered to enhance the manuscript’s rigor, transparency and relevance. They are detailed below in major and minor comments.

*MAJOR COMMENTS

Introduction

1/ p.6: The study aim is stated as follows: “this study aimed to describe the methodological characteristics and assess the reporting quality of qualitative studies published by Peruvian authors.” (p.6). In the eligibility criteria, authors stated: “Only studies in which at least 25% of participants were based in Peru were considered.” (p.11).

This is a bit confusing. Authors should specify clearly if it’s about qualitative studies published by Peruvian authors or qualitative studies conducted in Peru.

Methods

1/ p.6: Why focusing especially on 2022-2025 period to get recent studies? Authors have provided an explanation in Discussion. However, are the studies published prior 2022-2025 no more relevant or used to inform health practice and policy?

2/ p.6: Authors claim to have conducted a systematic search. Yet they only searched Scopus without a justification. Why did they search only one database and how inclusive/comprehensive do they think their review is? Did they consider other sources such as references lists of identified articles?

3/ p.7: How relevant do you think it would be to group studies by health disciplines? Researchers usually work in specific health disciplines and your findings would be more insightful if you provide them with discipline-based evidence.

4/ p.8: Authors used the highest tertile to define “adequate SRQR reporting”. Is this threshold a standard in the field or just authors’ choice? This should be clarified.

5/ p.8-11: This SRQR-based study assessment remains somehow subjective since the appreciation may vary by assessor. I believe that if another research group had to reproduce this review, they may give different scores to each study for each item. Are authors aware of this subjectivity and variability concern? What strategy did they implement to mitigate this limitation?

6/ It is widely recognized that study reporting quality depends on research expertise and vary both by health discipline (dentistry vs. public health) and by authors’ affiliation type (clinical vs. research institution affiliation). Since the information on health discipline was available, why did authors not consider it as a descriptive characteristic and subsequently test its association with adequate reporting categories?

Results

1/ Please format all tables according to the journal’s guidelines.

2/ Figure 1: authors stated that 287 studies were excluded by title and abstract. Can they briefly specify the reasons both in the figure and in the descriptive text?

3/ Figure 2: mandatory elements of a geographical map, such as direction (North) and gradient (scale), are missing. Please consider adding them.

4/ Adequate reporting is reported in Table 1 but not commented in the text. Please consider adding an in-text comment and a table footnote recalling how it was defined.

5/ p.15: It seems counterintuitive to interpret an association by the reference group as authors did for Table 2 regarding non-Peruvian vs. Peruvian first author’s affiliation (PR = 0.40, 95% CI: 0.26–0.62). If authors wish to keep this interpretation, I suggest them to assign the reference group to Peruvian authors.

Discussion

1/ Considering you research rationale; how do authors interpret and explain a study with one Peruvian author (for instance at sixth position) and many foreigner co-authors? Is it treated as a Peru-affiliated study at the same level as the one exclusively authored by Peru-based researchers?

2/ p.17: Please, authors should remove proportions from the Main Results recall in the Discussion section. Instead, they could use descriptive terms such as half of studies, three-quarter, one-third, etc. which are more appealing as summary.

3/ p.19-20: Authors compare their findings with the existing literature. They mainly focused on similar findings, while less is said about divergence between their results and the existing evidence. Moreover, less is said about the studies of comparison besides thematic areas. They should consider improving this.

4/ p.20: Authors should consider revising the paragraph #1 of the subsection “Factors associated with…” First, delete “…which could partially explain the observed association” because it is repetitive with the second sentence starting: “This may be explained by the tendency…”. Second, another explanation to consider may be the fact that English studies are often co-authored by international researchers, requiring English as the collaborative language and bringing additional level of rigor and expertise, as acknowledged in the subsequent paragraph.

5/ p.21-22: Two major limitations need to be considered. First, there is a rater subjectivity in using SRQR checklist that should not be dismissed, yet not discussed by authors. This can be observed in the Strengths paragraph where authors praise their “flexibility” in rating studies, which was even not mentioned in Methods. Second, regression analysis estimated crude associations that should be explicitly acknowledged in the discussion alongside their implications for both these findings and future research.

Conclusion

1/ p.22: The conclusion reads more like results recall than overall summary and practical recommendations for qualitative health research stakeholders. Authors should consider complying with the PRISMA-ScR recommendation relative to conclusion: “Provide a general interpretation of the results with respect to the review questions and objectives, as well as potential implications and/or next steps.”

* MINOR COMMENTS

1/ Please, revise the referencing format throughout the text according to the journal’s guidelines.

2/ p.6: I suggest to reformulate concisely the following text: From “A detailed study protocol was developed prior to study initiation and made publicly available. The protocol of this review is available at https://osf.io/jes6v/files/3rn52.” to “A detailed study protocol was developed prior to study initiation and made publicly available (https://osf.io/jes6v/files/3rn52)”

3/ p.7: Please, provide an internet link to Rayyan software to align with proper software referencing (city, company, website). Same for Stata software and revise as follows: “…Stata software, version 19.0.” (p.11)

4/ p.7: Please revise: “…with the collaboration of other authors.” or “…with the collaboration of another author.”

5/ A lot of paragraphs are too short, with only 1 to 3 sentences within a subsection. Please, revise to make paragraphs more compact.

6/ p.11: Please, use appropriate terminology: replace “numeric variables” by “quantitative variables”

7/ p.11: Please, add statistical threshold: “…crude prevalence ratios (PR) with 95% confidence intervals (CI) were estimated…”

8/ Please, reformulate Table 2 title as follows: “Factors associated with adequate…”

9/ I am just curious to know how long it took to check 147 studies against a 21-item checklist.

**********

6. PLOS authors have the option to publish the peer review history of their article (what does this mean?). If published, this will include your full peer review and any attached files.

If you choose “no”, your identity will remain anonymous but your review may still be made public.

**Do you want your identity to be public for this peer review?**   For information about this choice, including consent withdrawal, please see our Privacy Policy.

Reviewer #1: No

Reviewer #2: No

**********

[NOTE: If reviewer comments were submitted as an attachment file, they will be attached to this email and accessible via the submission site. Please log into your account, locate the manuscript record, and check for the action link "View Attachments". If this link does not appear, there are no attachment files.]

To ensure your figures meet our technical requirements, please review our figure guidelines: https://journals.plos.org/plosone/s/figures

You may also use PLOS’s free figure tool, NAAS, to help you prepare publication quality figures: https://journals.plos.org/plosone/s/figures#loc-tools-for-figure-preparation.

NAAS will assess whether your figures meet our technical requirements by comparing each figure against our figure specifications.

Attachment Submitted filename: PONE-D-26-05648-AF.pdf

# Author comment: Author response to Decision Letter 1

**DOI:** 10.1371/journal.pone.0351494.r002

30 Apr 2026

Dear editor,

Many thanks for your response to the submission of our paper

We sincerely thank you and the reviewers for their thoughtful and constructive comments regarding the submission of our manuscript. We have carefully revised the manuscript in response to all reviewers’ feedback and have addressed each suggestion in detail. We trust that the revisions undertaken have enhanced the clarity and overall quality of the manuscript. Our point-by-point responses to each comment are provided below.

Rubí Paredes-Angeles

rubiparedesa@gmail.com

Corresponding author

___________________________________________________________________________

Review Comments to the Author

Reviewer #1

Comment:

Introduction section, paragraph 4, page 6

The authors mentioned that health research publications have increased in Peru. They cited the source of that information. Although it is okay, they could have spiced up the argument by stating the magnitude of the increase.

Reply: In response, we have specified the magnitude of the increase in health research in Peru. The following sentence was added in Introduction section: “In Peru, scientific research output has increased, with an estimated annual growth rate of 13.6%, with 70.2% corresponds to health sciences [14]”

Comment: The authors have claimed that “to date, no studies have specifically examined qualitative health research or assessed the quality of its reporting.” The argument lacks clarity and evidence. Can they provide evidence that their study is of the first kind in Peru?

Study eligibility, paragraph 2, page 7

Reply: We have revised the statement to avoid an overgeneralized claim and improve clarity. The paragraph now reads in Introduction Section: “A previous study examined the scientific production of qualitative research in Peru found that the publication trend has increased since 2013; however, it did not assess the reporting quality of these studies [15].” This correction acknowledges prior evidence, incorporates the relevant reference [15], and recognizes the limitation regarding the absence of reporting quality assessment.

Comment: “Only studies in which at least 25% of participants were based in Peru were considered.” Why? Can this statement be supported with the reason for including studies in which at least 25% of participants were based in Peru?

Reply: We added the following sentence in the second paragraph of the “Study eligibility” section: “Only studies in which at least 25% of participants were based in Peru were considered to ensure meaningful representation of the Peruvian population while allowing the inclusion of international studies.”

Comment: Conclusion, pages 22 and 23

Conclusion lacks recommendations from the study’s findings.

Reply: The conclusion has been revised as follows: “This review identifies key areas for improvement in the reporting of qualitative health studies in the Peruvian population, including techniques to ensure trustworthiness and credibility, methodological approach, researcher reflexivity, title quality, and reporting of ethics approval. Editorial teams could encourage adherence to standardized reporting guidelines and make editorial policies more explicit regarding ethics committee approval and key elements of methodological transparency, thereby supporting improved reporting practices. Future research could investigate whether these challenges persist in other contexts with similar structural and geographical disparities.”

Comment: The authors have performed statistical analysis appropriately and rigorously. They have used simple statistical analyses by reporting prevalence ratios and frequencies. However, they should consider revising the presentation of the frequencies in Table 1. It is more appearing to arrange the frequencies of the variables in a table, either from highest to lowest percentages or vice versa. Table 1 is on pages 13 and 14.

Reply: The order of variables in Table 1 has been revised to present them in descending order according to their percentages.

Comment: The authors did not make the data underlying the findings in their manuscript fully available. However, they state that the data will be available upon reasonable request from the corresponding author.

Reply: We have revised the Data Availability Statement to improve transparency. It now states: “The raw data underlying the findings are available in the S4 Table.” Therefore, the complete data can be accessed in this supplementary material.

Comment: The manuscript is presented in an intelligible fashion and written in standard English. However, in the discussion section (main results, page 17), the paragraph is redundant to what has already been presented in the results section. Again, on pages 7 and 8, the Spanish was mentioned twice. Additionally, using the word “to date” (page 6) will be vague if the paper is read in 2 or more years from now. Can authors replace “to date” with other word(s)?

Reply: The “Main Results” paragraph in the Discussion section has been revised to avoid redundancy with the Results section. Specifically, numerical values were removed and replaced with approximate descriptive expressions (e.g., “nearly one quarter” instead of “24.5%”) to improve readability and reduce repetition. Regarding the language variable (pages 7–8), the wording was clarified to avoid repetition. It has been revised as follows: “language of the study (English, Spanish, or both English and Spanish)”. Finally, the expression “to date” has been removed and replaced with more precise wording, as mentioned before.

Reviewer #2:

Comment: Introduction. 1/ p.6: The study aim is stated as follows: “this study aimed to describe the methodological characteristics and assess the reporting quality of qualitative studies published by Peruvian authors.” (p.6). In the eligibility criteria, authors stated: “Only studies in which at least 25% of participants were based in Peru were considered.” (p.11).

This is a bit confusing. Authors should specify clearly if it’s about qualitative studies published by Peruvian authors or qualitative studies conducted in Peru.

Reply: To clarify this point, we revised the final sentence of the Introduction (study aim) as follows: “this study aimed to describe the methodological characteristics and assess the reporting quality of qualitative studies conducted in populations residing in Peru and involving at least one author affiliated with a Peruvian institution.” This revision clarifies that the study includes both research conducted in Peruvian populations and studies involving at least one author with a Peruvian institutional affiliation. This criterion is also consistently specified in the “Study eligibility” section.

Comment: Methods. 1/ p.6: Why focusing especially on 2022-2025 period to get recent studies? Authors have provided an explanation in Discussion. However, are the studies published prior 2022-2025 no more relevant or used to inform health practice and policy?

Reply: Our decision to restrict the analysis to studies published between 2022 and 2025 was not based on the assumption that earlier studies are no longer relevant. Rather, it was a deliberate methodological choice aligned with the objective of this scoping review, which was to describe the current state of reporting practices in qualitative health research conducted by Peruvian authors.

Focusing on recent years allows for a more accurate assessment of contemporary reporting standards, particularly in the context of the increasing dissemination and adoption of reporting guidelines such as SRQR. In addition, this timeframe reduces potential distortions associated with atypical publication patterns observed during the COVID-19 pandemic period.

Comment: 2/ p.6: Authors claim to have conducted a systematic search. Yet they only searched Scopus without a justification. Why did they search only one database and how inclusive/comprehensive do they think their review is? Did they consider other sources such as references lists of identified articles?

Reply: The decision to use Scopus as the sole database was based on its broad coverage and its capacity to filter results by authors’ institutional affiliation (i.e., affiliation with Peru), which was a key eligibility criterion in our review. To our knowledge, other commonly used databases such as PubMed, SciELO, or LILACS do not allow for such precise and reproducible filtering by affiliation, which limited their suitability for our specific objective. Regarding additional search strategies, we did not perform manual searches of reference lists. Given the scope of this review and the structured search strategy applied, we prioritized a reproducible and database-driven approach.

Therefore, our findings should be interpreted as representative of the qualitative health research indexed in Scopus rather than the entirety of qualitative research conducted in Peru. These limitations have been explicitly acknowledged in the revised manuscript.

Comment: 3/ p.7: How relevant do you think it would be to group studies by health disciplines? Researchers usually work in specific health disciplines and your findings would be more insightful if you provide them with discipline-based evidence.

Reply: Although we initially considered it, many of the included studies addressed interdisciplinary topics or involved overlapping populations and settings, which posed difficulties for a coherent and mutually exclusive classification into distinct health disciplines. Nevertheless, we recognize the potential value of this approach. In our study, we partially addressed this dimension by categorizing studies according to their main population and research setting, which may serve as proxies for disciplinary focus.

Comment: 4/ p.8: Authors used the highest tertile to define “adequate SRQR reporting”. Is this threshold a standard in the field or just authors’ choice? This should be clarified.

Reply: The use of the highest tertile to define “adequate SRQR reporting” was a methodological decision made by the authors rather than a standardized or universally established threshold in the field.

Currently, there is no cut-off point for categorizing reporting quality based on SRQR scores. In this context, we opted for a data-driven approach by classifying studies according to the distribution of scores within our sample. Defining “adequate reporting” as the highest tertile allowed us to identify studies with comparatively better adherence to SRQR items while maintaining sufficient variability for the analysis of associated factors. This approach has been used in previous methodological studies, where relative thresholds (e.g., tertiles or quartiles) are applied in the absence of validated cut-offs. However, we acknowledge that such categorization is somewhat arbitrary and should be interpreted cautiously.

We have added in Methods: “Given the absence of established or validated cut-off points for SRQR scores, this data-driven approach was used to identify studies with comparatively better reporting quality.”

Comment: 5/ p.8-11: This SRQR-based study assessment remains somehow subjective since the appreciation may vary by assessor. I believe that if another research group had to reproduce this review, they may give different scores to each study for each item. Are authors aware of this subjectivity and variability concern? What strategy did they implement to mitigate this limitation?

Reply: We acknowledge that SRQR-based assessments may involve a degree of subjectivity, as judgments can vary across assessors. To mitigate this, we implemented several strategies to enhance consistency and reproducibility. First, our data extraction matrix included two components for the SRQR score: one for the assigned score and another to document the rationale for that score, including verbatim excerpts from the reviewed articles to justify each rating. Second, each pair of investigators independently extracted study characteristics and SRQR items. They then met to compare each cell and reach consensus on the score assigned to each item, resulting in a final extraction. Discrepancies that could not be resolved were discussed during weekly team meetings until agreement was achieved. Additionally, prior to formal data extraction, the team conducted calibration exercises to ensure a shared understanding of SRQR criteria and consistent application across reviewers.

To clarify this, we have added in Methods section: “The data extraction matrix included two components for the SRQR score: one for the assigned score and another to document the rationale, including verbatim excerpts from the reviewed articles to justify each rating. Each pair of investigators independently extracted study characteristics and SRQR items, and then met to compare each cell and reach consensus, resulting in a final extraction. Discrepancies that could not be resolved were discussed during weekly team meetings until agreement was achieved.”

Additionally, we added the following statement to the limitations section of the discussion: "Fourth, although the SRQR provides a structured framework for evaluating reporting quality, the interpretation of some items may involve a degree of reviewer subjectivity."

Comment: 6/ It is widely recognized that study reporting quality depends on research expertise and vary both by health discipline (dentistry vs. public health) and by authors’ affiliation type (clinical vs. research institution affiliation). Since the information on health discipline was available, why did authors not consider it as a descriptive characteristic and subsequently test its association with adequate reporting categories?

Reply: We thank the reviewer for this comment. While we agree that reporting quality may vary across health disciplines, we did not include this variable due to challenges in consistently classifying studies into mutually exclusive categories, particularly for multidisciplinary research. As we mentioned above, this could introduce misclassification and affect the interpretability of the findings.

Comment: Results 1/ Please format all tables according to the journal’s guidelines.

Reply: All tables have been revised and reformatted to comply with the journal’s guidelines.

Comment: 2/ Figure 1: authors stated that 287 studies were excluded by title and abstract. Can they briefly specify the reasons both in the figure and in the descriptive text?

Reply: The reasons for exclusion at the title and abstract screening stage have now been specified in Figure 1 and clearly described in the corresponding section of the text. Specifically, the following sentence was added to the Results section: “Records were excluded at this stage because they did not meet the eligibility criteria, including studies that were not qualitative, were not related to health sciences, did not involve populations residing in Peru, or corresponded to non-original publications.”

Comment: 3/ Figure 2: mandatory elements of a geographical map, such as direction (North) and gradient (scale), are missing. Please consider adding them.

Reply: Figure 2 has been revised to include the mandatory cartographic elements, including the North direction and scale, in accordance with standard conventions.

Comment: 4/ Adequate reporting is reported in Table 1 but not commented in the text. Please consider adding an in-text comment and a table footnote recalling how it was defined.

Reply: An in-text comment has been added to the Results section, and a footnote has been included in Table 1 to clarify the definition of adequate reporting. Specifically, the following sentence was incorporated into the text: “Adequate SRQR reporting was observed in 32.7% of the included studies.”

Comment: 5/ p.15: It seems counterintuitive to interpret an association by the reference group as authors did for Table 2 regarding non-Peruvian vs. Peruvian first author’s affiliation (PR = 0.40, 95% CI: 0.26–0.62). If authors wish to keep this interpretation, I suggest them to assign the reference group to Peruvian authors.

Reply: Thank you for this valuable comment. However, we deliberately selected non-Peruvian affiliation as the reference category to maintain consistency

Attachment Submitted filename: Response to Reviewers .pdf

# Editor report: Decision Letter 1

**DOI:** 10.1371/journal.pone.0351494.r003

**Authors:** Alfredo Fort

29 May 2026

Calidad de la información en estudios cualitativos de salud publicados por autores peruanos: una revisión exploratoria

PONE-D-26-05648R1

Dear Dr. Paredes-Angeles,

We’re pleased to inform you that your manuscript has been judged scientifically suitable for publication and will be formally accepted for publication once it meets all outstanding technical requirements.

Within one week, you’ll receive an e-mail detailing the required amendments. When these have been addressed, you’ll receive a formal acceptance letter and your manuscript will be scheduled for publication.

An invoice will be generated when your article is formally accepted. Please note, if your institution has a publishing partnership with PLOS and your article meets the relevant criteria, all or part of your publication costs will be covered. Please make sure your user information is up-to-date by logging into Editorial Manager at Editorial Manager® and clicking the ‘Update My Information' link at the top of the page. For questions related to billing, please contact billing support.

If your institution or institutions have a press office, please notify them about your upcoming paper to help maximize its impact. If they’ll be preparing press materials, please inform our press team as soon as possible -- no later than 48 hours after receiving the formal acceptance. Your manuscript will remain under strict press embargo until 2 pm Eastern Time on the date of publication. For more information, please contact onepress@plos.org.

Kind regards,

Alfredo Luis Fort, M.D., M.Sc., Ph.D.

Academic Editor

PLOS One

Additional Editor Comments (optional):

Although the manuscript is judged to be accepted for publication, I have found a few small areas that can be improved to ensure the reader is able to see and understand better what is being described (see in attached file).

Attachment Submitted filename: PONE-D-26-05648_R1-AF.pdf

# Editor report: Acceptance letter

**DOI:** 10.1371/journal.pone.0351494.r004

**Authors:** Alfredo Fort

PONE-D-26-05648R1

PLOS One

Dear Dr. Paredes-Angeles,

I'm pleased to inform you that your manuscript has been deemed suitable for publication in PLOS One. Congratulations! Your manuscript is now being handed over to our production team.

At this stage, our production department will prepare your paper for publication. This includes ensuring the following:

* All references, tables, and figures are properly cited

* All relevant supporting information is included in the manuscript submission,

* There are no issues that prevent the paper from being properly typeset

You will receive further instructions from the production team, including instructions on how to review your proof when it is ready. Please keep in mind that we are working through a large volume of accepted articles, so please give us a few days to review your paper and let you know the next and final steps.

Lastly, if your institution or institutions have a press office, please let them know about your upcoming paper now to help maximize its impact. If they'll be preparing press materials, please inform our press team within the next 48 hours. Your manuscript will remain under strict press embargo until 2 pm Eastern Time on the date of publication. For more information, please contact onepress@plos.org.

You will receive an invoice from PLOS for your publication fee after your manuscript has reached the completed accept phase. If you receive an email requesting payment before acceptance or for any other service, this may be a phishing scheme. Learn how to identify phishing emails and protect your accounts at https://explore.plos.org/phishing.

If we can help with anything else, please email us at customercare@plos.org.

Thank you for submitting your work to PLOS ONE and supporting open access.

Kind regards,

PLOS ONE Editorial Office Staff

on behalf of

Dr. Alfredo Luis Fort

Academic Editor

PLOS One
